# Ablation of CDK6 and STAT5B rewires T cell developmental dynamics and prevents ALK^+^ lymphoma formation

**DOI:** 10.1038/s41467-026-77466-4

**Published:** 2026-09-15

**Authors:** Jonatan Kendler, Maximilian Sexl, Alessia Schirripa, Elisabeth Gamper, Hager El Kersh, Barbara Maurer, Reinhard Grausenburger, Klavdija Krause, Dua Shahid, Alexander Pichler, Giorgia Damonte, Tania Brandstoetter, Michaela Prchal-Murphy, Stefania Massari, Sabine Lagger, Lukas Kenner, Richard Moriggl, Philipp B. Staber, Veronika Sexl, Sebastian Kollmann, Karoline Kollmann, Thorsten Klampfl

**Affiliations:** 1 Department of Biological Sciences and Pathobiology, Pharmacology and Toxicology, University of Veterinary Medicine, Vienna, Austria; 2 Department of Internal Medicine I (Oncology, Hematology, Clinical Immunology, and Rheumatology), Saarland University Medical School, Homburg, Germany; 3 Department of Medicine I, Division of Hematology and Hemostaseology, Medical University of Vienna, Vienna, Austria; 4 Department of Experimental Medicine, University of Genoa, Genoa, Italy; 5 Department of Biological Sciences and Pathobiology, Unit of Pathology, University of Veterinary Medicine, Vienna, Austria; 6 Department of Pathology, Medical University of Vienna, Vienna, Austria; 7 Christian Doppler Laboratory for Applied Metabolomics, Medical University of Vienna, Vienna, Austria; 8 Center for Biomarker Research in Medicine, CBmed GmbH, Graz, Austria; 9 Comprehensive Cancer Center, Medical University of Vienna, Vienna, Austria; 10 Department of Molecular Biology, Umeå University, Umeå, Sweden; 11 Department of Biosciences and Medical Biology, Paris Lodron University of Salzburg, Salzburg, Austria; 12 University of Innsbruck, Innsbruck, Austria

**Keywords:** T-cell lymphoma, Cancer genetics

## Abstract

CDK6 and STAT5B are well-established oncogenic drivers, but their interplay during transformation remains poorly understood. Here, we demonstrate that simultaneous genetic deletion of *Cdk6* and *Stat5b* in an *NPM-ALK*^*Tg/+*^ mouse model completely prevents tumor formation. Immature DN2/3 thymocytes are identified as the cellular origin of ALK-driven malignancy. Single-cell transcriptomics reveals a tumor-specific differentiation trajectory that is fully disrupted by combined *Cdk6/Stat5b* loss, coinciding with a developmental shift. We uncover shared transcriptional targets of CDK6 and STAT5B involved in cell-cycle regulation and apoptosis at the tumor initiation stage. In human ALCL cells, dual deletion of CDK6 and STAT5B induces context-dependent synthetic lethality, exposing a potential therapeutic vulnerability. A transcriptional signature derived from CDK6/STAT5B-dependent tumor initiation genes identifies a high-risk subgroup of ALCL patients with worse survival. Together, these findings establish a molecular link between thymocyte development, the crosstalk of CDK6 and STAT5B, oncogenic signaling, and clinical outcome in ALCL.

## Abstract (web summary)

Although CDK6 and STAT5B are established oncogenic drivers, how they cooperate during lymphoma initiation remains poorly understood. The authors show that combined Cdk6 and Stat5b deletion completely prevents ALK-driven anaplastic large cell lymphoma (ALCL) tumorigenesis, and suggest a prognostic signature linked to poor outcome in ALCL.

## Introduction

ALK-positive anaplastic large cell lymphoma (ALK^+^ALCL) is an aggressive peripheral T cell non-Hodgkin lymphoma primarily affecting children and adolescents^1,2^. In approximately 90% of pediatric and adolescent patients, a t(2;5)(p23;35) translocation generates the oncogenic fusion protein Nucleophosmin-Anaplastic Lymphoma Kinase (NPM-ALK)^3–5^. ALK becomes constitutively active when dimerized via NPM, driving STAT3/5 (Signal transducer and activator of transcription 3/5) oncoprotein activation^6–9^. Current therapies, including CHOEP-based chemotherapy (cyclophosphamide, doxorubicin, vincristine, etoposide and prednisone) or ALK inhibitor regimens, are often effective, but associated with toxicity, and chemotherapy is not curative for all patients^10–14^. A subset of patients, particularly those with resistance to chemotherapy or ALK inhibition continue to present clinical challenges^15,16^. Given resistance mechanisms and potential long-term side effects, a better understanding of oncogenic and inter-patient heterogeneity is critical^17^.

T cell development within the thymic cortex begins with double-negative (DN) CD4^-^CD8^-^ thymocytes. DN1 (CD44^+^CD25^-^), DN2a and DN2b (CD44^+^CD25^+^) cells represent the most immature developmental stages. At the DN2b/3a stage, T cell receptor (TCR) gene rearrangement is initiated by VDJ recombination. Thymocytes at this stage bifurcate into two distinct lineages, as they either activate a γδ developmental program^18^ or they rearrange the TCRβ locus, committing to the αβ T cell lineage^19^. In the latter case, cells further differentiate via the DN3a and DN3b (CD44^-^CD25^+^) stages. After pre-TCRα formation, DN4 (CD44^-^CD25^-^) cells progress via an immature single positive stage (ISP; CD8^+^(mouse) or CD4^+^(human)) to double positive (DP) thymocytes, which mature into CD4/CD8 T cells in the medulla upon positive selection^20^.

Although ALK^+ ^ALCL is firmly established as a T cell neoplasm, the identity of its cell of origin within T cell development is still unresolved^21–23^. Emerging evidence, however, suggests that ALK^+^ALCL arises from early intra-thymic progenitors^24–26^. Most ALK^+^ALCL tumor cells express at least one T cell marker^27^ but typically lack cell-surface TCR expression. TCR rearrangements are detectable in around 75% of cases^24^. However, the precise developmental window of transformation and the cooperating oncogenic pathways are still incompletely defined^23^.

Oncogenic ALK signaling activates JAK-STAT, RAS-RAF-MAPK and PI3K-AKT-mTOR pathways^22^. In this context, STAT3 has long been considered the central effector of ALK signaling, regulating survival, proliferation, immune evasion and repression of T cell identity genes^6^. Furthermore, NPM-ALK circumvents physiological TCR signaling by directly activating downstream effectors^22^. Thus, ALK not only bypasses but rewires canonical TCR signaling, transforming a normally tightly regulated pathway into an oncogenic driver. Recently, CDK6 (Cyclin dependent kinase 6) and STAT5B (Signal transducer and activator of transcription 5B) emerged as key ALCL drivers^7,28^. STAT5B, a direct target of NPM-ALK^9^, is vital for proliferation and survival^7^, while CDK6 is highly expressed. It not only promotes cell-cycle progression but also angiogenesis, with its function as a transcriptional regulator^28^. In healthy thymic development, CDK6 and STAT5B regulate cell-cycle entry, proliferation and differentiation during early developmental stages^29–33^.

Using transgenic NPM-ALK mice^34^, we demonstrate that tumor initiation and progression critically depend on CDK6 and STAT5B. STAT5B and CDK6 cooperate to reprogram T cell fate, orchestrating transformation. Single-cell RNA sequencing (scRNA-seq) and flow cytometry analysis identify DN2/3 thymocytes as the cell of origin. Loss of CDK6/STAT5B impairs transformation and uncovers a transcriptional signature predictive of improved patient survival, establishing them as key oncogenic drivers and clinical determinants in ALCL.

## Results

### Absence of NPM-ALK-driven tumor formation upon genetic *Cdk6* and *Stat5b* loss

**Fig. 1:**
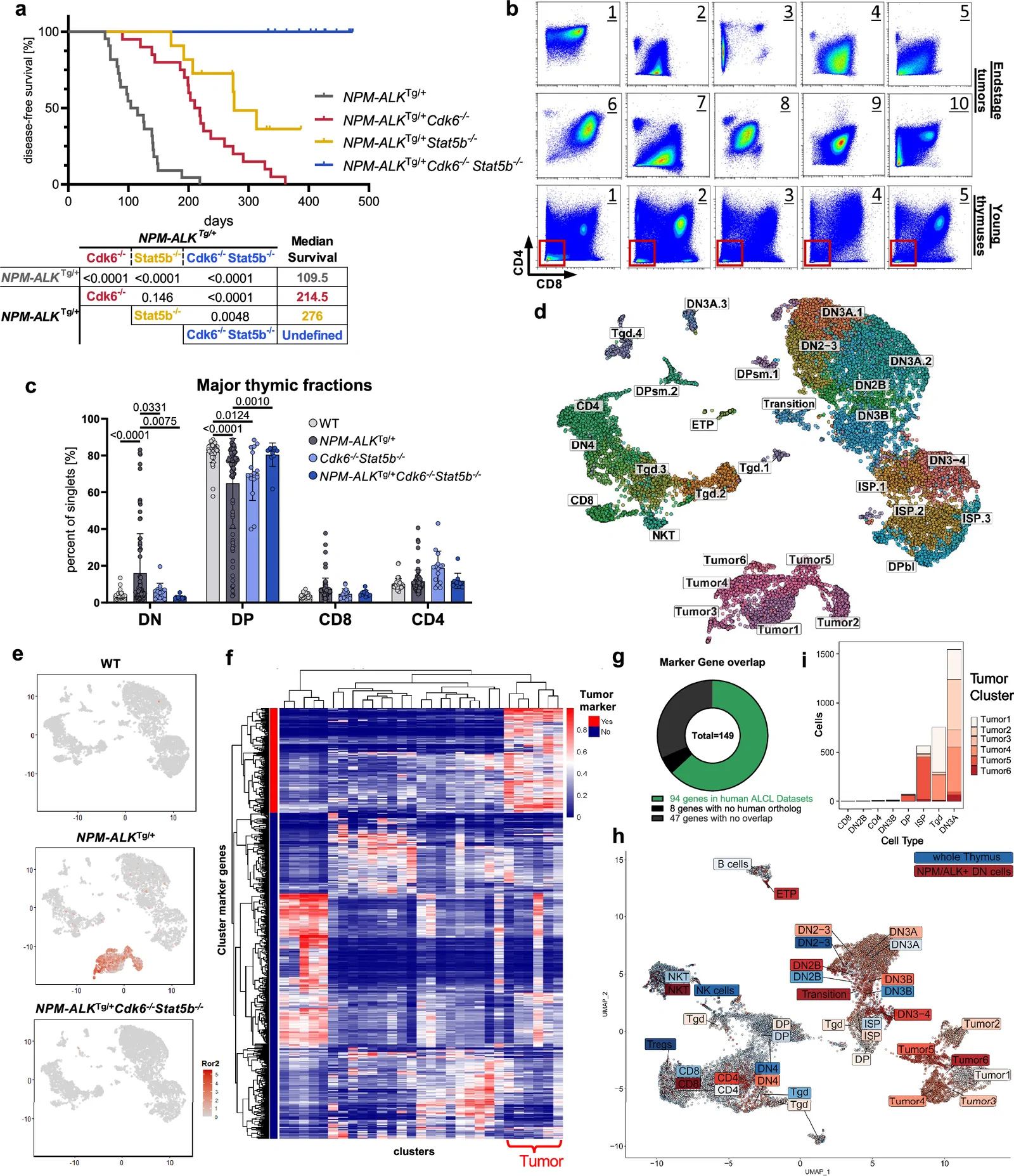
Genetic loss of *Cdk6* and *Stat5b* prevents NPM-ALK-driven tumor formation. **a** Kaplan-Meier plot analysis of *NPM-ALK*^Tg/+^ mice. *NPM-ALK*^Tg/+^ (*n* = 22), *NPM-ALK*^Tg/+^*Cdk6*^−/−^ (*n* = 20), *NPM-ALK*^Tg/+^*Stat5b*^−/−^ (*n* = 11), *NPM-ALK*^Tg/+^C*dk6*^−/−^Stat5b^−/−^ (*n* = 13); *p* values determined by log rank test with Bonferroni correction for multiple comparisons. **b** Representative flow cytometry analysis plots of the major T cell fractions of 10 thymuses with progressed tumors (1st and 2nd row) and of 5 thymuses from mice at ~10 weeks of age (3rd row; red rectangles highlight the DN fraction). **c** Analysis of major T cell fractions in the thymus of WT (*n* = 45), *NPM-ALK*^Tg/+^ (*n* = 79), *Cdk6*^−/−^*Stat5b*^−/−^ (*n* = 15) and *NPM-ALK*^Tg/+^*Cdk6*^−/−^*Stat5b*^−/−^ (*n* = 11) mice (Lin^-^Thy1.2^+^CD4^+/-^CD8^+/-^; age from 7-12.5 weeks). Significances between genotypes within each fraction were determined using two-way ANOVA with Tukey’s multiple comparisons test. Bars represent the mean; error bars indicate standard deviation. **d** Uniform Manifold Approximation and Projection (UMAP) plot of scRNA-seq data from FACS sorted thymic DN T cells from WT, *NPM-ALK*^Tg/+^ and *NPM-ALK*^Tg/+^*Cdk6*^−/−^*Stat5b*^−/−^ mice (20620 cells from *n* = 3 mice: all female, age 10 weeks). **e** UMAP plots from scRNA-seq of WT, *NPM-ALK*^Tg/+^ and *NPM-ALK*^Tg/+^*Cdk6*^−/−^*Stat5b*^−/−^ DN T cells with *Ror2* expression indicated (log_2_ normalized counts). **f** Unsupervised hierarchical clustering of all cluster markers (median AUC > 0.65). The six tumor clusters and the 149 tumor marker genes are highlighted. **g** Overlap of 149 tumor marker genes with markers from human ALCL patients. **h** Integration of whole thymus scRNA-seq data (shades of blue), with data from *NPM-ALK*^Tg/+^ DN cells (shades of red). **i** Results of a label transfer from whole thymus scRNA-seq clusters onto the six tumor clusters.

To study the effects of a combined deficiency of STAT5B with CDK6 on NPM-ALK-driven tumorigenesis, we established single and double knock-out *NPM-ALK*^*Tg/+*^ mice (Supplementary Fig. 1a). While overall survival was prolonged in mice lacking *Cdk6* or *Stat5b*, we observed a complete absence of tumor development in *NPM-ALK*^Tg/+^*Cdk6*^*−/−*^*Stat5b*^*−/−*^ mice (Fig. 1a). To test whether the absence of tumors in *NPM-ALK*^Tg/+^*Cdk6*^*−/−*^*Stat5b*^*−/−*^ mice is due to a cell-intrinsic effect, we performed cytokine-free colony formation assays using bone marrow cells transduced with NPM-ALK. Both, the number of colonies and the total cell output were significantly reduced in the combined absence of *Cdk6* and *Stat5b* (Supplementary Fig. 1b-d). As observed in human patients^24,35^, we detected a high CD4/CD8 surface marker heterogeneity in our end-stage tumors. However, 7-12.5 week old *NPM-ALK*^*Tg/+*^ mice showed a consistent expansion of the DN compartment (Fig. 1b), which was missing in age-matched *NPM-ALK*^*Tg/+*^*Cdk6*^*−/−*^*Stat5b*^*−/−*^ mice (Fig. 1c and Supplementary Fig. 1e, f). To uncover altered molecular mechanisms in the DN fraction between WT, *NPM-ALK*^*Tg/+*^ and *NPM-ALK*^*Tg/+*^*Cdk6*^*−/−*^*Stat5b*^*−/−*^ mice, we performed scRNA-seq of DN cells and identified all thymic DN subpopulations (Fig. 1d and Supplementary Fig. 1g). In the *NPM-ALK*^Tg/+^ sample, six additional cell clusters were identified, which were not found in the WT or *NPM-ALK*^Tg/+^*Cdk6*^*−/−*^*Stat5b*^*−/−*^ samples. The elevated expression of ALCL markers like ROR2^36^, CD44^24^ and CD30 (TNFRSF8)^3,10^ in these clusters classified them as tumor cells^37^ (Fig. 1d, 1e and Supplementary Fig. 1h). A detailed marker gene analysis, combined with unsupervised hierarchical clustering, identified a set of 149 genes that were almost exclusively expressed in the tumor cell clusters (Fig. 1f). 94 of these genes were found to be upregulated in samples from ALCL patients, further validating that cells in tumor clusters indeed resemble ALCL cells (Fig. 1g and Supplementary Fig. 1i). We confirmed that *ALK* mRNA expression of NPM-ALK^+^ cells was unaltered in *NPM-ALK*^*Tg/+*^*Cdk6*^*−/−*^*Stat5b*^*−/−*^ cells compared to *NPM-ALK*^*Tg/+*^ controls (Supplementary Fig. 1j).

As the tumor clusters formed a distinct and separated group of cells, we sought to determine whether the tumor cells arose from the DN compartment. Therefore, we integrated our scRNA-seq data with a publicly available reference dataset from the whole mouse thymus^38^. The majority of thymocytes from our scRNA-seq dataset clustered with their respective counterparts from the whole thymus, but the identified tumor cells remained a separate cluster, marking them as transcriptionally distinct from untransformed thymocytes (Fig. 1h). Label transfer analysis of the tumor cluster to the whole thymus confirmed its highest similarity to the DN compartment (Fig. 1i). These findings provide evidence that NPM-ALK-driven tumors originate from DN thymocytes and combined loss of *Cdk6* and *Stat5b* completely abrogates tumor initiation.

### NPM-ALK-driven tumors originate from DN2/DN3 thymic progenitor cells

**Fig. 2:**
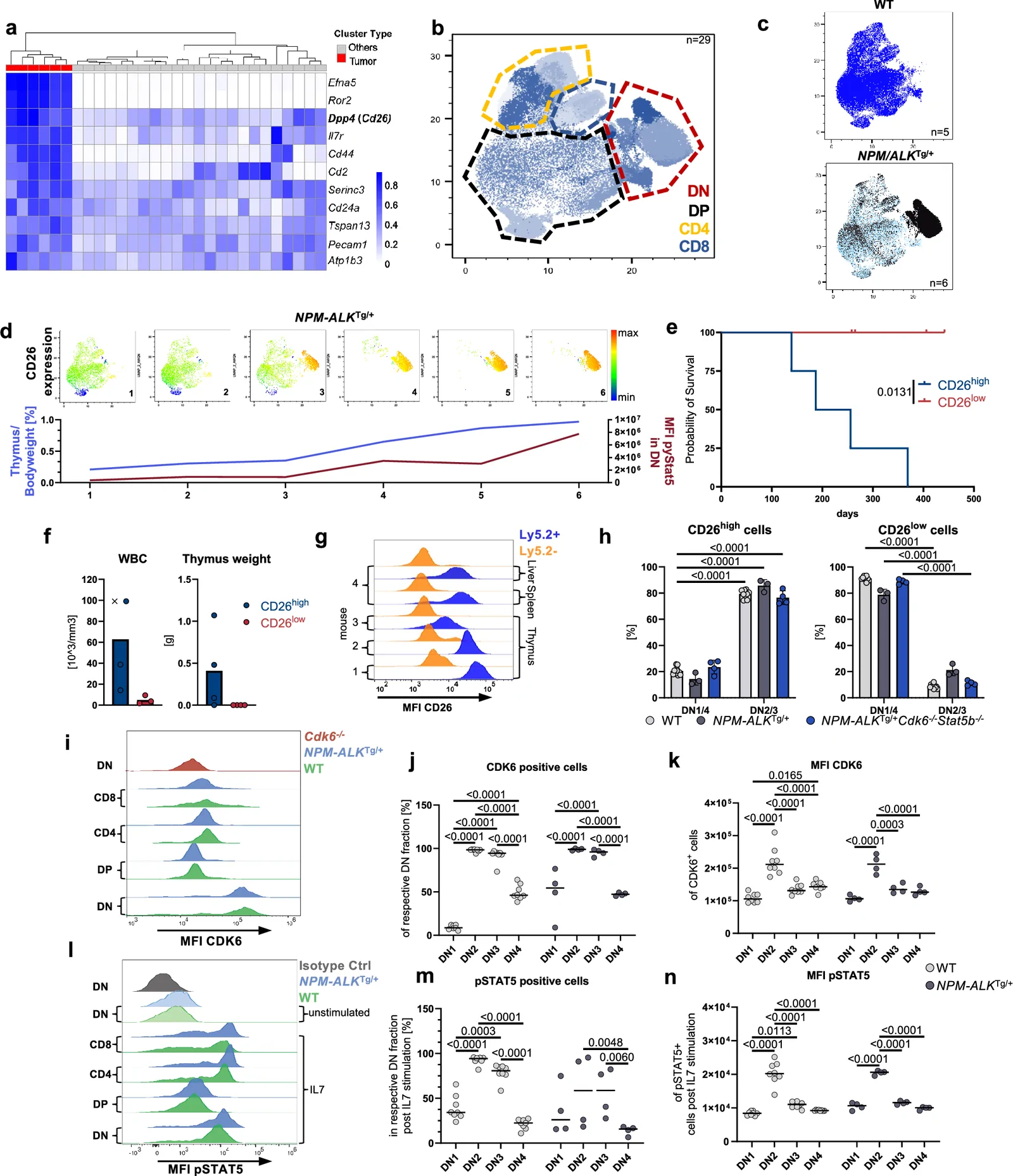
CD26^high^ DN2/3 T cells initiate tumors and have high pSTAT5 and CDK6 levels. **a** Surface markers among the tumor cluster markers shown in Fig. 1f, ordered by mean expression in tumor clusters. **b** UMAP plots (*n* = 29) from flow cytometry data of lineage negative cells stained for CD4, CD8, CD44, CD25, IL7R, TCRb, CD26 and CD24. **c** Selected genotypes from (b) (WT (*n* = 5); *NPM-ALK*^Tg/+^ (blue: WT; lightblue: sample 1–3 from  (d), black: sample 3-6 from (d) **d** Six *NPM-ALK*^Tg/+^ samples from (b, c), ordered by thymus-weight/body-weight (age sample 1–5 = 12.5 weeks, sample 6 = 16 weeks). UMAP plots show CD26 levels; thymus-weight/body-weight as well as the MFI of pSTAT5 are plotted below. **e** Kaplan-Meier analysis of NSG mice, injected with 8*10^4^ sorted *NPM-ALK*^Tg/+^ thymic DN T cells (CD26^high^ or CD26^low^; *p* values determined by log rank test). **f** White blood-cell count and thymus weight from mice in (**e**). x marks a measurement at the upper detection limit of the hematology analyzer. **g** Flow cytometry analysis of 4 transplanted neoplastic mice from (**e**) showing CD26 expression in injected (Ly5.2^+^) and host cells (Ly5.2^-^) from selected organs. **h** CD26 levels across the DN1-4 compartments in WT (*n* = 10), *NPM-ALK*^Tg/+^ (*n* = 3) and *NPM-ALK*^Tg/+^*Cdk6*^−/−^*Stat5b*^−/−^(*n* = 4) mice. **i** Representative CDK6 expression in major thymic compartments. **j**, **k** CDK6-positive cells and median fluorescent intensity (MFI) within the DN1-4 thymocyte subsets in WT (*n* = 8) and *NPM-ALK*^Tg/+^(*n* = 4) mice. **l** Representative pSTAT5 levels after IL7 stimulation. **m**, **n** pSTAT5-positive cells and MFI across the DN1-4 fractions in WT (*n* = 8) and *NPM-ALK*^Tg/+^(*n* = 4). For (**h**–**n**), significance between genotypes within each fraction was determined using two-way ANOVA with Tukey’s multiple comparisons test. Bars represent mean, lines median, error bars indicate standard deviation.

Among the 94 identified tumor markers (Fig. 1f, g and Supplementary Fig. 1i), we found 11 cell surface proteins (Fig. 2a and Supplementary Fig. 2a). We used 4 of the 11 markers (CD24, CD26 (DPP4), CD44 and IL7R) in combination with a conventional T cell panel for flow cytometric analysis. This allowed for the identification of a distinct tumor cluster within the DN compartment that was exclusively present in the *NPM-ALK*^*Tg/+*^ thymuses, reflecting our scRNA-seq findings (Fig. 2b, c and Supplementary Fig. 2b). CD26 performed not only best to distinguish this cluster but its levels were consistently high in human and mouse cell lines (Supplementary Fig. 2c, d), positively correlated with thymus to bodyweight ratio in the DN compartment and reached its highest levels in the tumor cluster (Fig. 2d). To test the potential of CD26 as a phenotypic marker, CD26^high^ or CD26^low^ expressing DN cells from *NPM-ALK*^Tg/+^ mice were transplanted into NSG mice (Fig. 2e and Supplementary Fig. 2e). Only mice receiving CD26^high^ DN cells developed disease exhibiting elevated white blood cell counts, enlarged thymuses and a CD26+ cell expansion in either thymus (3 cases) or spleen/liver (1 case; Fig. 2f, g). In contrast to CD26^low^ thymocytes, CD26^high^ cells were strongly enriched for DN2/3 cells (Fig. 2h), indicating that the tumor originates from the DN2/3 compartment. Analyzing the cell-cycle stages of WT and *NPM-ALK*^Tg/+^ DN1 to DN4 thymocytes revealed that *NPM-ALK*^Tg/+^ cells already displayed increased proliferation at the DN2 stage (Supplementary Fig. 2f). Established murine cell lines derived from primary NPM-ALK-driven tumors, similar to human ALCL lines and patient samples, lacked surface expression of TCRγδ and TCRβ (Supplementary Fig. 2c), despite harboring rearranged TCR loci (Supplementary Fig. 2g, h). Transcriptomic comparison of murine tumor cell lines, human ALCL lines, and patient samples revealed highly conserved activation of the three major ALCL oncogenic pathways, indicating that murine tumors recapitulate key molecular hallmarks of human ALCL (Supplementary Fig. 2i).

To study the abundance of CDK6 and STAT5B in DN2/3 cells, we analyzed CDK6 and phosphorylated STAT5 (pY694/699; pSTAT5) levels from ex vivo WT and untransformed *NPM-ALK*^Tg/+^ thymocytes by intracellular flow cytometry. Early thymocyte development was marked by elevated CDK6 levels in DN cells (Fig. 2i), particularly within the DN2/DN3 subsets (Fig. 2j), with peak levels in DN2 (Fig. 2k). IL7 stimulation, a widely used STAT5 stimulus in DN thymocytes^31,39,40^, led to robust induction of pSTAT5, particularly in DN2 and DN3 cells (Fig. 2l, m), with the strongest signal in DN2 cells (Fig. 2n). Consistent with a coordinated role of CDK6 and pSTAT5 during early transformation, scRNA-seq analysis revealed that *Cdk6, Stat5b* and *Dpp4* (encoding CD26) are concomitantly upregulated along the tumor trajectory at the DN2 to DN3 transition, with DN2/3 cells expressing high *Dpp4*, also showing elevated *Cdk6* and *Stat5b* levels (Supplementary Fig. 2j, k). In line, *DPP4* expression positively correlated with both *CDK6* and *STAT5B* expression in two independent ALCL patient cohorts (Supplementary Fig. 2l). These findings support the notion that tumor initiating CD26^high^ thymocytes are predominantly DN2/3 cells, characterized by high CDK6 levels and robust IL7 mediated pSTAT5 activation.

### Absence of CDK6 and STAT5B signaling rewires developmental pathways and halts tumor formation

**Fig. 3:**
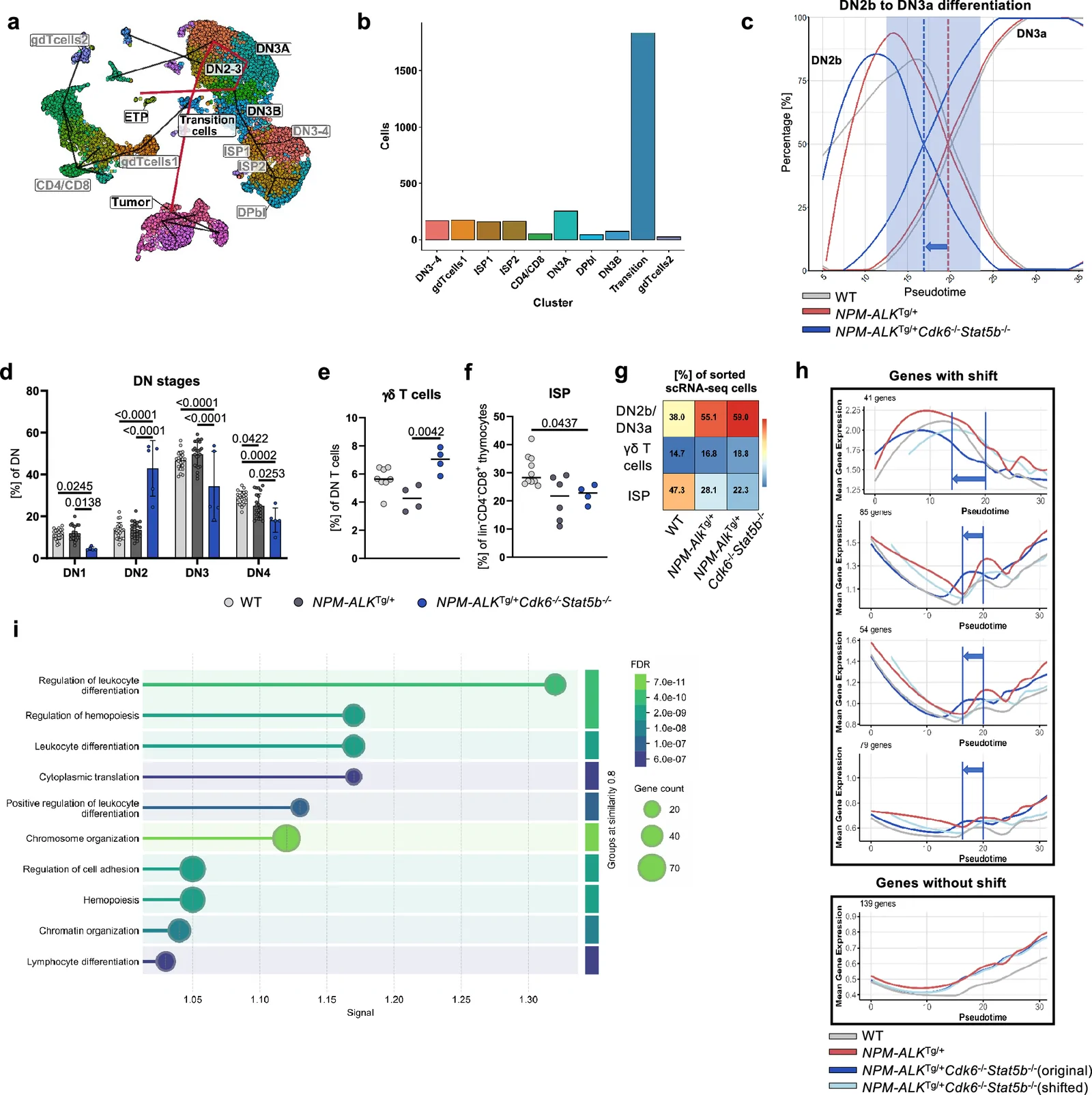
Developmental shift occurs at the tumor initiating stage upon CDK6 and STAT5B loss. **a** Trajectory analysis of scRNA-seq data from DN *NPM-ALK*^Tg/+^ cells. **b** Label Transfer analysis mapping the 6 tumor clusters onto all DN clusters in (a). **c** Proportion of cells per pseudotime interval classified as DN2b or DN3a (Immgen reference). Transition from DN2b to DN3a is indicated by the blue area. Data are shown for WT (gray), *NPM-ALK*^Tg/+^ (red) and *NPM-ALK*^Tg/+^*Cdk6*^−/−^*Stat5b*^−/−^ (blue). **d** Flow cytometry analysis of thymic DN subfractions (Lin^-^Thy1.2^+^CD4^-^CD8^-^) in WT (*n* = 21), *NPM-ALK*^Tg/+^ (*n* = 23) and *NPM-ALK*^Tg/+^*Cdk6*^−/−^*Stat5b*^−/−^ (*n* = 5) mice. Statistical significance between genotypes within each DN subfraction was determined using two-way ANOVA with Tukey’s multiple comparisons test. Bars represent the mean; error bars indicate standard deviation. **e** Flow cytometry results of γδ-TCR in the DN fraction (WT (*n* = 8), *NPM-ALK*^Tg/+^ (*n* = 4) and *NPM-ALK*^Tg/+^*Cdk6*^−/−^*Stat5b*^−/−^ (*n* = 4)) and **f**  of immature single positive cells (ISP; Lin^-^CD8^+^TCRB^-^CD24^hi^). Statistical significance between genotypes was determined using a Kruskal-Wallis test with Dunn’s multiple comparisons test (WT (*n* = 10), *NPM-ALK*^Tg/+^ (*n* = 6) and *NPM-ALK*^Tg/+^*Cdk6*^−/−^*Stat5b*^−/−^ (*n* = 4). **g** Percentage of DN2b/DN3a, γδ T cells and ISP cells within scRNA-seq data of WT, *NPM-ALK*^Tg/+^ and *NPM-ALK*^Tg/+^*Cdk6*^−/−^*Stat5b*^−/−^. **h** Four representative gene modules showing the most pronounced shifts in expression along pseudotime and a control group lacking the shift. **i** Enrichment of Gene Ontology (GO) Biological Processes in shift-associated genes (https://string-db.org/).

To understand the role of CDK6 and STAT5B in tumor development, we performed trajectory analysis on the scRNA-seq data to trace cellular differentiation (Fig. 3a). We identified a tumor-specific trajectory (red line) leading from the most immature cells (early thymic progenitors: ETP; subset of DN1) via the DN2/3 compartment to the tumor. An unambiguous annotation of an intermediate cluster connecting DN2/3 cells to the tumor cluster was not possible, as it showed cell type assignments of lower confidence and a diverse set of αβ and γδ-T cell annotations (Supplementary Fig. 3a). We therefore termed this cluster “transition cluster” (Fig. 3a). This cluster was present across all genotypes. An independent label transfer-analysis of the tumor cells confirmed the transition cluster as the closest related cluster to the tumor cells (Fig. 3b). This observation is not shared by WT and *NPM-ALK*^*Tg/+*^*Cdk6*^*−/−*^*Stat5b*^*−/−*^ samples, indicating that the transition cluster in the *NPM-ALK*^*Tg/+*^ sample is transcriptionally “re-programmed” (Supplementary Fig. 3b).

To address potential differences in DN development between the three genotypes, we focused on dynamic gene expression changes along the tumor-specific trajectory within each genotype (Fig. 3a and Supplementary Fig. 3c). We divided the tumor-specific trajectory into intervals and performed differential gene expression analysis between *NPM-ALK*^*Tg/+*^ and *NPM-ALK*^*Tg/+*^*Cdk6*^*−/−*^*Stat5b*^*−/−*^ samples at each interval. The number of deregulated genes peaked at the beginning of the DN2b to DN3a transition (Supplementary Fig. 3d, e). Comparing relative abundance of DN2b and DN3a cells along the pseudotime, we observed a developmental shift in the *NPM-ALK*^*Tg/+*^*Cdk6*^*−/−*^*Stat5b*^*−/−*^ sample (Fig. 3c). In line, flow cytometric analysis of DN stages showed an accumulation of DN2 and a reduction of DN3 cells in the absence of CDK6 and STAT5B (Fig. 3d, Supplementary Fig. 3f). Trajectory analysis further revealed three distinct developmental paths emerging from DN2/3 thymocytes (Fig. 3a). These include the two physiological paths into either ISPs or γδ T cells, and a third trajectory in the *NPM-ALK*^*Tg/+*^ cells, leading towards the tumor, reminiscent of transdifferentiation phenomena^41^. Flow cytometric and scRNA-seq analyses positioned *NPM-ALK*^*Tg/+*^*Cdk6*^*−/−*^*Stat5b*^*−/−*^ mice at the highest proportion of γδ T cells among the three genotypes. In contrast, ISP cells were reduced in the presence of NPM-ALK (Fig. 3e, f, g). The data confirm a shift in differentiation and a partial block at a stage where both factors are required for physiological and oncogenic progression.

To identify genes associated with that shift, we focused on the 2000 most variable genes along the tumor trajectory and grouped them into 18 gene modules based on shared expression dynamics. Seven showed above-median expression shifts between *NPM-ALK*^*Tg/+*^ *and NPM-ALK*^*Tg/+*^*Cdk6*^*−/−*^*Stat5b*^*−/−*^ (Fig. 3h (shift indicated with blue arrow) and Supplementary Fig. 3g, h). In line, enrichment analysis of the shifted genes revealed involvement in leukocyte and lymphocyte differentiation, chromosome/chromatin organization and cell adhesion (Fig. 3i).

### CDK6 and STAT5B engage an active chromatin landscape at the DN2/3 crossroad

**Fig. 4:**
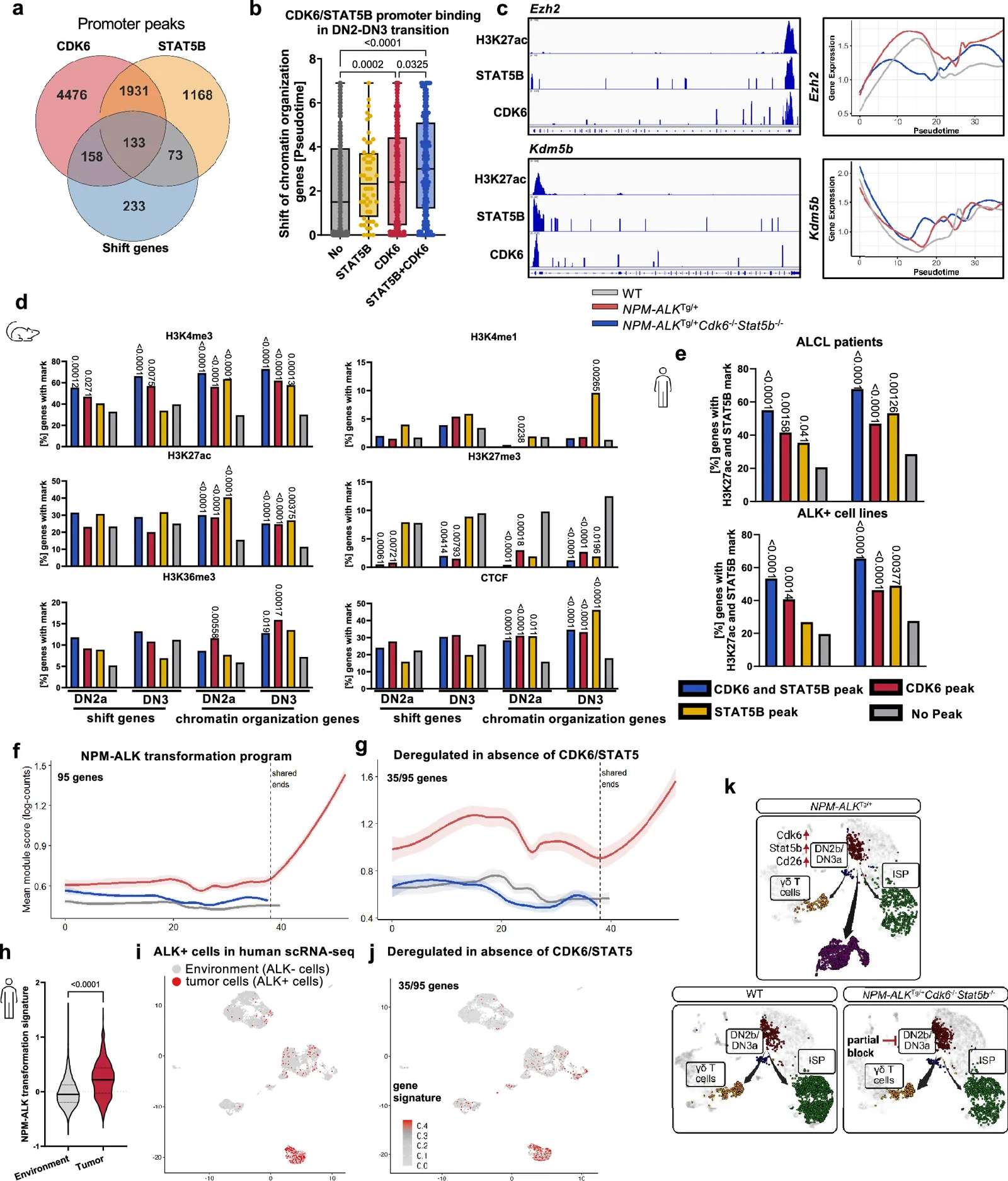
CDK6 and STAT5B engage an active chromatin landscape at the DN2/3 crossroad. **a** Overlap of genes with shifted expression (blue), with genes carrying a STAT5B (yellow) or a CDK6 (red) ChIP-seq promoter peak (−300/ + 200 bp; STAT5B ChIP-seq: DN2 cells ±IL7 stimulation, CDK6 ChIP-seq: murine *NPM-ALK*^Tg/+^ cell line). **b** Pseudotime shift of chromatin organization genes during the DN2 to DN3 transition, stratified by CDK6/STAT5B promoter occupancy. Each dot represents one chromatin organization pathway gene; *n* denotes the number of genes per group (No (*n* = 462), STAT5B (*n* = 52), CDK6 (*n* = 329), STAT5B + CDK6 (*n* = 242)). Box plots are displayed with all dots; center line represents median, bounds represent 25th and 75th percentiles; whiskers from minimum to maximum; statistics by Kruskal-Wallis test with Dunn’s test for multiple comparisons. **c** Two of the 133 genes from (a) that showed promoter peaks for CDK6 and STAT5B and had shifted expression along pseudotime in the *NPM-ALK*^Tg/+^*Cdk6*^−/−^*Stat5b*^−/−^sample. H3K27ac signals were added. **d** Percentage of murine shift genes and chromatin organization genes carrying the indicated promoter-proximal marks ( ± 1000 bp around the TSS) in DN2a and DN3 pro-T cells (ImmGen), stratified by CDK6/STAT5B promoter occupancy (two-sided Fisher’s exact test versus No Peak). **e** Percentage of shift and chromatin organization genes with concurrent H3K27ac and STAT5B occupancy (TSS ± 1000 bp) in primary ALCL patients and ALK^+^ cell lines, stratified by CDK6/STAT5B promoter occupancy (two-sided Fisher’s exact test versus No Peak). **f** Mean score of the 95-gene NPM-ALK transformation signature along tumor trajectory, shown as loess fit of the mean per genotype (line) with the 95% confidence interval (shaded band); dashed line marks the last point all three genotypes share. **g** As in (**f**) for the 35-gene CDK6/STAT5B-dependent subset. **h** 95-gene score in ALK⁺ (red) vs ALK⁻ (grey) cells from a CD45⁺-sorted human ALK⁺ ALCL biopsy (*n* = 1; 7554 cells; median and quartiles shown; two-tailed Mann-Whitney). **i** UMAP of biopsy, colored by ALK status. **j** Same UMAP, colored by the 35-gene subset score. **k** Graphical summary of developmental routes from the DN2b/DN3a stage, bifurcating into the αβ and γδ T cells, illustrates divergence of trajectories.

To study whether these genes are directly regulated by STAT5B and/or CDK6, we analyzed ChIP-seq data and identified 364 genes with CDK6, STAT5B or both peaks at their promoter. Importantly, 133 genes were co-bound and shifted, suggesting high sensitivity to CDK6 and STAT5B loss (Fig. 4a). Network analysis showed interactions with key regulators of T cell development and proliferation, like CCND2, RUNX1, JUN, JAK2, IL2RA, PIAS2, SOCS3 and KIT (Supplementary Fig. 4a). As it is well established that successful T-cell differentiation depends on the concerted action of chromatin modifying enzymes^42,43^, we asked if the observed alterations were direct or indirect consequences of CDK6 and STAT5B loss. Examining genes of the chromatin organization gene set during the DN2 to DN3 transition revealed that CDK6/STAT5B co-bound genes showed the largest shifts, indicating that dual occupancy maximizes transcriptional sensitivity (Fig. 4b). Among the direct targets of CDK6 and STAT5B was *Ezh2*, a core member of the PRC2 complex, the depletion of which was previously linked to the shutdown of genes that drive T-cell differentiation^44–46^. The histone demethylase KDM5B represses target genes by de-methylating activating histone marks^47^. The shifted expression of these chromatin modifiers might therefore explain the observed shift of DN2/3 expression profiles (Fig. 4c) and suggests altered developmental dynamics in T-cell differentiation in the absence of CDK6 and STAT5B. Additional co-bound chromatin regulators included *Ctcf, Arid1a, Yy1, Smarca5, Dnmt1, Rad21*, and *Hdac2*, with known roles in T-cell development^43,48–52^, indicating broad involvement of CDK6 and STAT5B in chromatin organization (Supplementary Fig. 4b). To determine whether this regulation occurs within a permissive chromatin environment, we profiled histone modifications at the DN2a and DN3 stage, the precise stage at which the transcriptional shifts occur. CDK6/STAT5B co-bound chromatin organization genes were consistently enriched for the activating promoter and enhancer marks H3K4me3, H3K27ac, and H3K4me1, defining a landscape of actively regulated genes. In contrast, at shift gene promoters CDK6/STAT5B binding was predominantly associated with reduced H3K27me3 relative to unbound shift genes, consistent with release from Polycomb-mediated repression during the DN2 to DN3 transition (Fig. 4d). The same loci further showed enrichment of the elongation mark H3K36me3 and of CTCF, pointing to a contribution of higher-order chromatin organization (Fig. 4d). Translating this analyses to human samples, H3K27ac profiling of primary ALCL biopsies and ALK^+^ and ALK^−^ cell lines confirmed enrichment of active chromatin marks at STAT5B and CDK6 co-bound gene programs identified in the mouse, supporting cross species conservation of this potential regulatory mechanism (Fig. 4e and Supplementary Fig. 4c, d**)**.

In addition to the observed shift in differentiation, we investigated whether the NPM-ALK related transcriptional program is affected by CDK6 and STAT5B loss. Comparing NPM-ALK^+^ cells along the tumor trajectory with WT cells, we defined a 95 gene NPM-ALK transformation signature, of which 35 genes were specifically deregulated in the absence of CDK6 and STAT5B (Fig. 4f, g). The relevance of these deregulated genes extends to human ALK + ALCL, where the subset is specifically expressed in ALK+ tumor cells of a primary biopsy analysed by scRNA-seq (Fig. 4h-j) In summary, the absence of tumor development observed in *NPM-ALK*^*Tg/+*^*Cdk6*^*−/−*^*Stat5b*^*−/−*^ mice is associated with a partial developmental block at the DN2/3 stage, an imbalance of differentiation towards γδ T cells and the failure to engage a CDK6/STAT5B dependent core program of NPM-ALK transformation (Fig. 4k).

### CDK6 and STAT5B transcriptionally regulate shared and distinct target genes during transformation

**Fig. 5:**
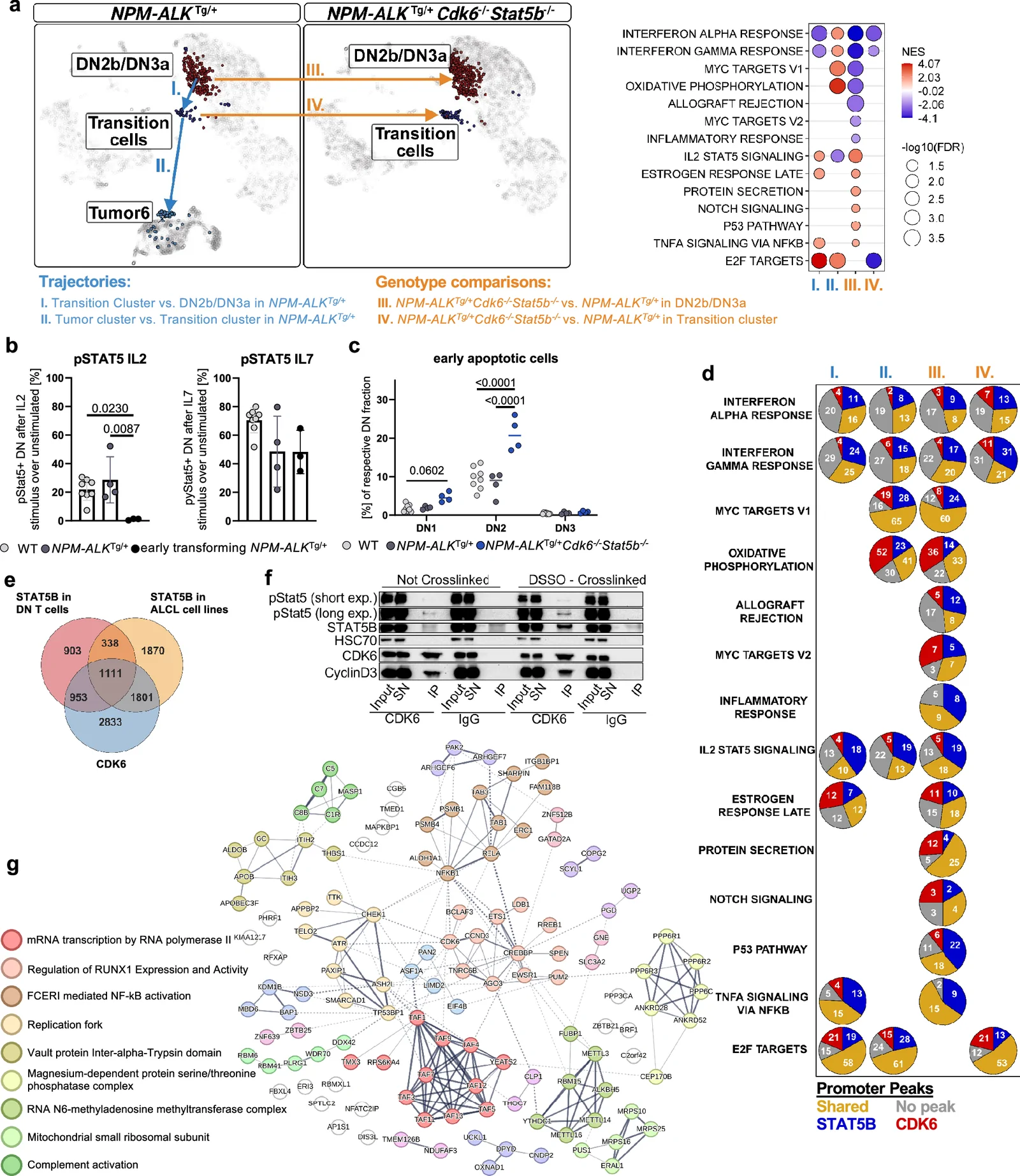
CDK6 and STAT5B co-regulate transformation pathways. **a** Trajectory-focused comparisons (I. and II. (blue labels)) follow transcriptional changes along the differentiation toward the tumor cluster in UMAP plots. Genotype-focused comparisons (III. and IV. (orange labels)) contrast *NPM-ALK*^Tg/+^ with *NPM-ALK*^Tg/+^*Cdk6*^−/−^*Stat5b*^−/−^within DN2b/DN3a and transition clusters (left). Bubble plots show GSEA results (Hallmark database) for trajectory- and genotype-focused comparisons (right). Pathways deregulated in both settings (FDR < 0.1) are shown. Bubble size reflects the -log_10_(FDR), color indicates normalized enrichment score (NES). **b** pSTAT5 levels in DN thymocytes following IL2 or IL7 stimulation. Data show normalized pSTAT5 signal (% of DN fraction) across genotypes: WT (*n* = 8), *NPM-ALK*^Tg/+^ (*n* = 4), *NPM-ALK* ^Tg/+^*Cdk6*^−/−^*Stat5b*^−/−^(*n* = 4) and *NPM-ALK*^Tg/+^ early tumor (*n* = 3). One-way ANOVA followed by Tukey’s multiple comparisons test was performed. Bars represent mean and error bars indicate standard deviation. **c** Flow cytometry analysis of early apoptotic (Annexin^+^) DN1-DN3 thymocytes (Lin^-^Thy1.2^+^CD4^-^CD8^-^CD44^+/-^CD25^+/-^). Frequencies were quantified in WT (*n* = 8), *NPM-ALK*^Tg/+^ (*n* = 4) and *NPM-ALK* ^Tg/+^*Cdk6*^−/−^*Stat5b*^−/−^(*n* = 4) mice. Two-way ANOVA with Tukey’s multiple comparisons test was performed. **d** Pie charts show numbers of core-enriched genes sets from Fig. 4A. Shown are genes with CDK6 and/or STAT5B ChIP-seq promoter peaks (−300/ + 200; red: CDK6 only, blue: STAT5B only, yellow: both and grey: neither). **e** Venn diagram of genes with STAT5B promoter peaks in murine DN2 cells (+/-IL7), human ALCL cell lines, and CDK6 promoter peaks in murine cells. Promoter regions were defined as -300 to +200 bp relative to the transcription start site. **f** Western blot of CDK6 IP in a human ALCL cell line (SU-DHL-1) +/- crosslinker DSSO. IgG was the negative control, Cyclin D3 the positive control and HSC70 the loading control. This experiment has been performed twice, with and without crosslinking. **g** STRING PPI network of the 129 stable interactors (confidence ≥ 0.4), clustered by MCL (inflation = 2). Edge thickness = combined interaction score; solid = within-cluster, dashed = between-cluster. Node color = MCL cluster; functional annotations are listed. Unclustered nodes are shown in white. Only annotated clusters with more than 4 proteins are shown in the legend.

The DN2b/DN3a cells and the neighbouring transition cluster, which represent the last two developmental stages before the tumor cluster, were analyzed for transcriptional changes between our genotypes (Fig. 5a). Along the trajectory towards the tumor in the NPM-ALK^Tg/+^ sample (blue arrows), we observed an increase in interferon response pathways, MYC targets, oxidative phosphorylation and E2F targets. All these pathways were downregulated in the *NPM-ALK*^*Tg/+*^*Cdk6*^*−/−*^*Stat5b*^*−/−*^ sample (orange arrows; Fig. 5a and Supplementary Fig. 5a). Surprisingly, IL2-STAT5 signalling was downregulated in the tumor. Since IL7 stimulation led to elevated pSTAT5 signals in DN2/3 cells (Fig. 2m, n) and *Il7r* was among the highest expressed tumor marker genes (Fig. 2a), we hypothesized that earliest transformation is driven preferentially by IL7-STAT5 signaling rather than IL2-STAT5 signalling. Supporting this, IL7, but not IL2, sustained pSTAT5 activity in early transforming cells (Fig. 5b). Notably, in *NPM-ALK*^*Tg/+*^*Cdk6*^*−/−*^*Stat5b*^*−/−*^ DN2b/DN3a cells, IL2 signaling appeared selectively upregulated (Fig. 5a and Supplementary Fig. 5b), with a trend of reduced IL7R levels in DN2 (Supplementary Fig. 5c), and increased IL2RA levels both transcriptionally and on the surface (Supplementary Fig. 5d, e). These data indicate a shift toward IL2 reliance in *NPM-ALK*^*Tg/+*^*Cdk6*^*−/−*^*Stat5b*^*−/−*^ DN2b/DN3a while IL7-STAT5B signaling dominates during transformation. This is supported by promoter binding of STAT5B to more than 700 IL7-induced putative target genes in human ALCL cell lines (Supplementary Fig. 5f).

At the DN2b/DN3a to transition cluster junction, apoptotic programs were exclusively activated in *NPM-ALK*^*Tg/+*^*Cdk6*^*−/−*^*Stat5b*^*−/−*^ cells (Fig. 5c and Supplementary Fig. 5g, h). DN2 cells showed increased apoptosis when CDK6 and STAT5B were missing, partially explaining the reduced number of DN3 cells (Fig. 3d) and the inhibition of tumorigenesis at this stage in *NPM-ALK*^*Tg/+*^*Cdk6*^*−/−*^*Stat5b*^*−/−*^ mice.

Integration of core-enriched genes from the most up- or downregulated pathways (Fig. 5a**)** with CDK6 and STAT5B ChIP-seq data revealed extensive co-binding of both proteins at promoters of key transformation-associated genes. This was especially pronounced in genes involved in MYC, TNFA, E2F and interferon response pathways (Fig. 5d). Comparative ChIP-seq analysis showed that the majority (67.5%) of all STAT5B-bound promoter regions in DN2/3 cells were also co-occupied by CDK6 in ALCL cells, with 2190 genes overlapping across datasets. In ALCL cell lines, CDK6 and STAT5B co-occupied 2887 promoter regions, underscoring a potential cooperative role in transcriptional regulation during transformation (Fig. 5e). To address whether CDK6 and STAT5B are part of the same transcriptional complex, we performed co-immunoprecipitation experiments. CDK6 was shown to interact with STAT5B and pSTAT5 (Fig. 5f and Supplementary Fig. 5i). To analyze the transcriptional interaction partners of CDK6 in more detail, we performed a CDK6 immunoprecipitation followed by mass spectrometry analysis in human ALCL cell lines. By overlapping the results of the control and Palbociclib treated cell lines, we identified 129 core interaction partners of CDK6 (Supplementary Fig. 5j, k). STRING network analysis revealed several transcriptional regulators present in CDK6-containing complexes. Enrichment analysis of these proteins highlighted RNA polymerase II preinitiation complex (PIC) assembly, transcription preinitiation complex assembly, protein-DNA complex assembly, and transcription factor TFIID complex as the most significantly enriched terms (Fig. 5g and Supplementary Fig. 5l, m). Consistent with the histone modifying interaction partners, CDK6/STAT5B co-bound regions are enriched for active chromatin marks (H3K4me3, H3K27ac) and depleted of repressive H3K27me3 (Fig. 4d and e). Overall, Palbociclib treatment did not alter the core interaction network.

### Reciprocal pathway activation in the absence of CDK6 or STAT5B

**Fig. 6:**
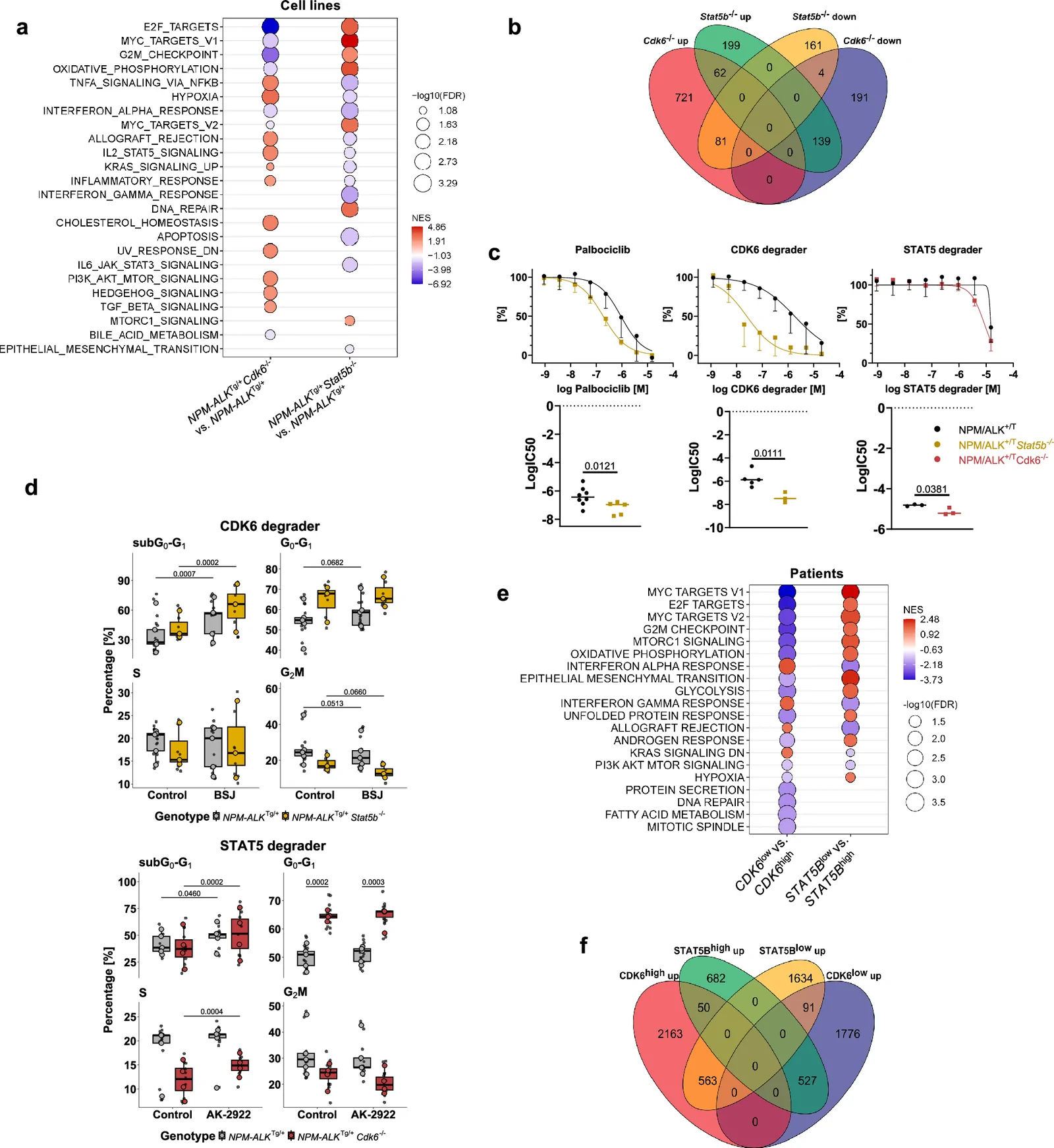
Reciprocal pathway activation is observed in the absence of CDK6 or STAT5A. **a** Deregulated Pathways with an FDR < 0.1 in mouse thymus derived *NPM-ALK*^Tg/+^*Cdk6*^−/−^ (CDK6KO) and *NPM-ALK*^Tg/+^*Stat5b*^−/−^ (BKO) cell lines compared to *NPM-ALK*^Tg/+^ (TG) cell lines. GSEA of 6 TG cell lines (3 biological replicates), 6 CDK6KO (3 biological) and 3 BKO cell lines (2 biological). **b** Venn diagram highlighting the cross-regulated pathways in *NPM-ALK*^Tg/+^Cdk6^−/−^ (Cdk6^−/−)^ or *NPM-ALK*^Tg/+^Stat5b^−/−^ (Stat5b^−/−^) compared to the *NPM-ALK*^Tg/+^ cell lines. **c** Treatment of *NPM-ALK*^Tg/+^*Cdk6*^−/−^ and *NPM-ALK*^Tg/+^ cell lines with the STAT5 PROTAC degrader AK-2292 and *NPM-ALK*^Tg/+^Stat5b^−/−^ and NPM-ALK^Tg/+^ cell lines with Palbociclib and the CDK6 PROTAC degrader BSJ-03-123. Statistical analysis used unpaired two-tailed t-tests (AK-2292, BSJ-03-123) and a paired two-tailed t-test (Palbociclib). Top row: IC_50_ curves (points: mean; error bars: standard deviation); bottom row: IC_50_ comparisons (lines show median; points indicate cell lines from separate transgenic mice; AK-2292: *n* = 3 vs. 3; BSJ-03-123: *n* = 5 vs. 3; Palbociclib: *n* = 5 vs.3 assayed in two independent experiments. **d** Propidium iodide cell-cycle analysis. Top: *NPM-ALK*^Tg/+^ (*n* = 5 cell lines) and *NPM-ALK*^Tg/+^Stat5b^−/−^ (*n* = 3 cell lines) with DMSO (vehicle) or CDK6 PROTAC degrader BSJ-03-123 at IC_50_ in triplicate. Bottom: *NPM-ALK*^Tg/+^ (*n* = 5 cell lines) and *NPM-ALK*^Tg/+^Cdk6^−/−^ (*n* = 4 cell lines) cell lines treated with either DMSO (vehicle) or the STAT5 PROTAC degrader AK-2292 at 6 µM in triplicate. Data were analyzed using linear mixed-effects models with the cell line identifier included as a random intercept. Pairwise comparisons were performed, and *p* values were adjusted for multiple testing using the Tukey method. Box plots show biological replicate means (large colored points, means; small black points, technical replicates; Box center line, median; bounds, 25th–75th percentiles (IQR); whiskers, 1.5×IQR). **e** GSEA analysis from ALK^+^ALCL patients stratified by CDK6 or STAT5B expression. Bubble size reflects -log_10_(FDR), and color indicates the normalized enrichment score (NES; red: positive enrichment, blue: negative enrichment). **f** Venn diagram showing the overlap of genes associated with high CDK6 or STAT5B expression in ALK^+^ ALCL patients. Genes are extracted from the rank-ordered gene list used for Fig. 5e ( | cutoff | > 0.3).

To distinguish CDK6- and STAT5B-dependent transcriptional alterations, we studied tumor-derived *NPM-ALK*^*Tg/+*^ cell lines from mice with and without CDK6 or STAT5B. Transcriptional analyses uncovered reciprocal pathway regulation in the absence of either CDK6 or STAT5B. CDK6-dependent transcriptional programs were upregulated in STAT5B-deficient cells and vice versa (Fig. 6a, b and Supplementary Fig. 6a). To examine if *STAT5B* knock-out cell lines have a higher dependency on CDK6 signaling, we treated *NPM-ALK*^*Tg/+*^ and *NPM-ALK*^*Tg/+*^*Stat5b*^*−/−*^ cell lines with a CDK6 degrader, BSJ-03-123, or the CDK4/6 kinase inhibitor, Palbociclib. *NPM-ALK*^*Tg/+*^*Stat5b*^*−/−*^ cells showed higher sensitivity to CDK6 inhibition than *NPM-ALK*^*Tg/+*^ cell lines. Additionally, CDK6-deficient cells displayed increased sensitivity to AK-2292, a STAT5 degrader^53^ (Fig. 6c and Supplementary Fig. 6b-f). Cell-cycle analysis revealed that CDK6- or STAT5B-deficient cells exhibited the highest percentage of sub-G_0_/G_1_ populations upon pharmacological inhibition of the complementary factor (Fig. 6d). Reciprocal pathway activation observed in murine cell lines could be partially recapitulated in ALK^+^ALCL patient data stratified according to *CDK6* or *STAT5B* expression levels. We found elevated E2F and MYC target signatures, G_2_/M checkpoint activation, and oxidative phosphorylation in STAT5B-low tumors (Fig. 6e). Importantly, genes positively correlated with either *CDK6* or *STAT5B* expression in patients showed minimal overlap and frequently displayed inverse correlation patterns (Fig. 6f), validating the results from our murine cell lines.

### Dual deletion of CDK6 and STAT5B induces synthetic lethality in human ALCL cells in a context dependent manner

**Fig. 7:**
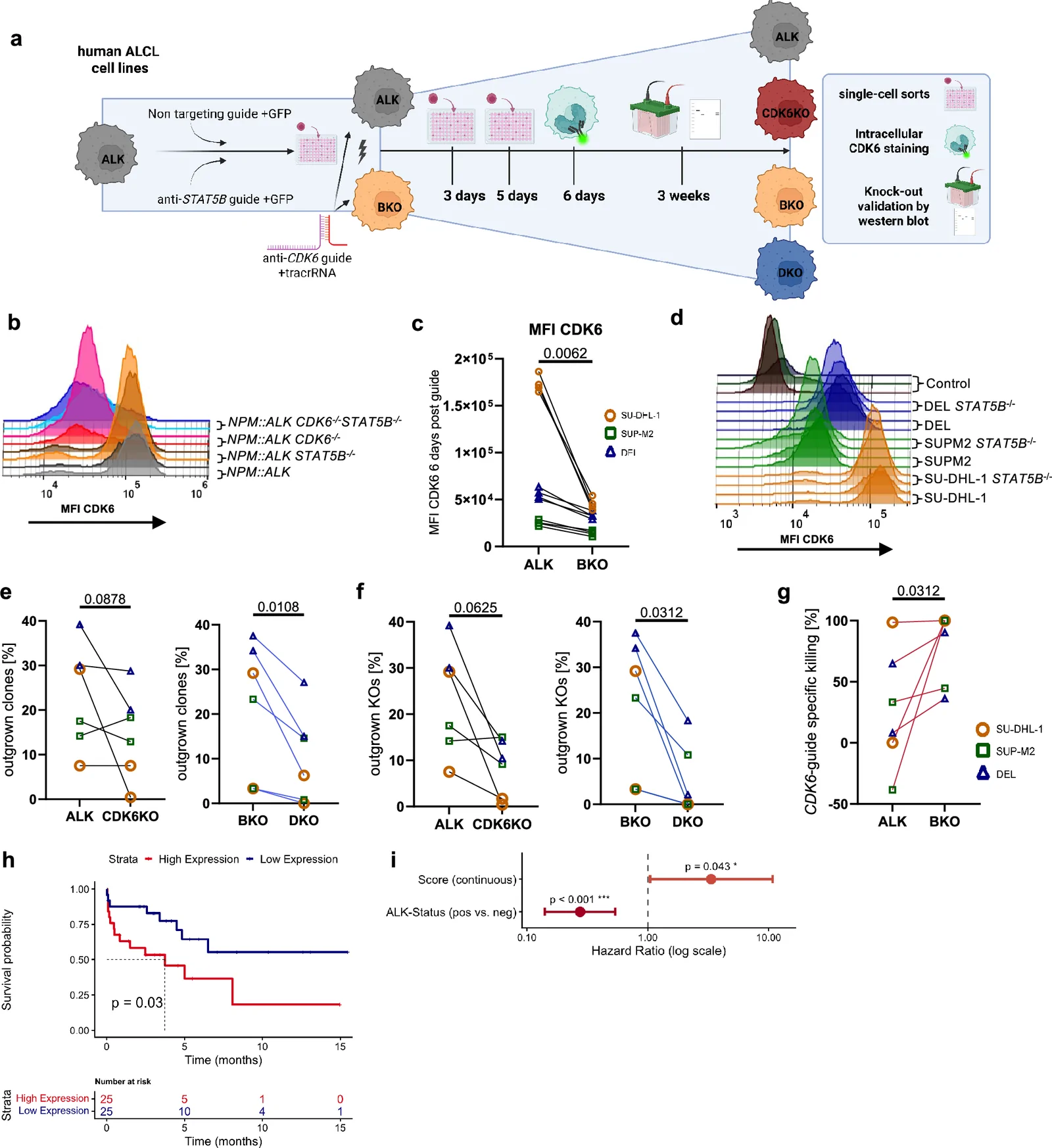
Dual CDK6 and STAT5B loss drives context-dependent synthetic lethality and stratifies human ALCL patient survival. **a** Experimental scheme showing the workflow of generating and testing CRISPR-Cas9 mediated *STAT5B* and *CDK6* knockouts in human NPM-ALK expressing ALCL cell lines (SU-DHL-1, DEL, SUP-M2) with and without a STAT5B knock-out (parental *NPM::ALK* (ALK), *NPM::ALK STAT5B*^−/−^ (BKO), *NPM::ALK CDK6*^−/−^ (CDK6KO), *NPM::ALK CDK6*^−/−^*STAT5B*^−/−^ (DKO). **b** Representative histogram overlay plots showing CDK6 levels in guide-positive cells (CDK6KO and DKO) compared with controls. **c** Quantification of MFI reduction six days post electroporation (right) of individually derived single cell clones *n* = 12 (4 clones from each of 3 independent human cell lines). Statistical significance was determined using a paired two-tailed t-test. **d** Representative histogram overlay plots showing CDK6 levels in the indicated cell line. **e** Quantification of total outgrown clones after 6 weeks of cultivation. 240 single cell clones were sorted from each of 3 different human ALCL cell lines using technical duplicates; paired two-tailed t-test. **f** Quantification of total verified knockout clones after 6 weeks. 240 single-cell clones were sorted from each of 3 different human ALCL cell lines using technical duplicates; paired two-tailed t-test. **g** Percentage of CDK6 guide-specific killing in ALK and BKO cell lines; paired two-tailed t-test. **h** Kaplan–Meier overall survival curves for ALCL patients (*n* = 74, 36 events; ALK^+^ = 51, ALK^− ^= 23; three independent cohorts) stratified by high (red, top tercile) versus low (blue, bottom tercile) expression of the 52-gene CDK6/STAT5B signature; patients in the middle tercile were excluded (*n* = 50). Log-rank *p* = 0.03. Tick marks indicate censored observations; the number-at-risk table is shown below. **i** Forest plot of the multivariate Cox proportional hazards model fitted on the 74 ALCL patients, including the signature score as a continuous variable and ALK status (positive vs. negative, with ALK- as the reference category) as covariates. Points indicate the hazard ratio (point estimate); error bars indicate 95% confidence intervals; the dashed vertical line marks HR = 1 *p* values are derived from two-sided Wald tests. No adjustment for multiple comparisons was applied, as each covariate was pre-specified.

We next assessed the effects of CDK6 and STAT5B loss in human ALCL lines by generating CRISPR-mediated knockouts. We made use of *NPM-ALK* expressing DEL, SU-DHL1 and SUP-M2 lines (ALK) and first established *STAT5B*-deficient cell lines (BKO) (Fig. 7a and Supplementary Fig. 7a). ALK and BKO cells were then transfected with a guide RNA targeting *CDK6*^54,55^ to generate CDK6-deficient (CDK6KO) and STAT5B/CDK6 double knock-out (DKO) cells, respectively (Fig. 7a). Three and five days post transfection, we sorted single cells to generate clones from each cell line and genotype. Overall, reduced CDK6 expression was determined in the remaining bulk cultures that had been transduced with a CDK6-targeting guide, indicating efficient gene knock-down (Fig. 7b and Supplementary Fig. 7b). The magnitude of depletion in each line was modulated by its baseline CDK6 expression (Fig. 7c, d). After three weeks, we assessed overall single-cell derived clonal outgrowth. We observed a significant reduction in DKO-derived colony numbers compared to BKO numbers, whereas CDK6KO-derived colonies exhibited on average similar outgrowth to their parental ALK counterparts (Fig. 7e). All grown colonies were genotyped and the proportion of surviving KO clones was assessed (Fig. 7f and Supplementary Fig. 7c). *CDK6* deletion resulted in a significantly stronger killing in cells lacking STAT5B compared to ALK cells (Fig. 7g). Together, these results indicate that while individual loss of either *CDK6* or *STAT5B* is tolerated across all tested cell lines, their combined loss results in a complete absence of viable clones in one line and significantly impaired outgrowth of the other cell lines. These data are consistent with context-dependent synthetic lethality.

### Patients expressing a *Cdk6*^*−/−*^*Stat5b*^*−/−*^ associated transcriptional profile showed survival benefit

To investigate if the transcriptional changes observed in association with *NPM-ALK*^*Tg/+*^*Cdk6*^*−/−*^*Stat5b*^*−/−*^ could be of clinical relevance, we compared them to ALCL patient data. A set of 52 genes that was downregulated at the tumor initiating DN2b/3a stage in the absence of CDK6 and STAT5B (Fig. 5a, III.), was found to be predictive for overall survival in a cohort of 74 ALCL patients. Stratifying patients by expression of the gene set into three equally sized groups (low, intermediate and high expressors; 25, 24 and 25 patients, respectively) revealed that patients with high expression of the gene set showed significantly worse survival than patients with low expression (Fig. 7h). Multivariate COX regression on the full cohort using gene set expression as a continuous predictor together with ALK status demonstrated the prognostic value of the gene set independent of ALK status. (HR = 3.35, 95% CI: 1.04–10.80, *p* = 0.043; Fig. 7i). This result was confirmed using an independent approach of dichotomizing the cohort (HR = 3.49, 95% CI: 1.22–10.03, p = 0.020; concordance index ~0.71; Supplementary Fig. 7d, e). These genes appear to represent a transcriptional program critical for tumor establishment in the mouse. Lower average expression of the homologous genes in ALCL patients was associated with prolonged survival, suggesting that the failure to activate this early oncogenic program impairs tumor fitness and aggressiveness. This gene signature may serve as a clinically relevant prognostic marker. Applied to a public dataset of other peripheral T cell lymphomas, the signature did not significantly stratify outcome (Supplementary Fig. 7f), consistent with a program preferentially relevant to ALCL.

## Discussion

We identified a cooperative role for CDK6 and STAT5B as essential drivers during NPM-ALK-mediated thymic transformation. Using knockout models, single-cell transcriptomics, flow cytometry and functional assays, we demonstrated that combined loss of these factors completely abrogated tumor development, disrupted thymocyte differentiation, impaired key oncogenic programs, and induced apoptosis.

While STAT3 has long been considered the central effector of ALK signaling^8,56^, it was shown that it is dispensable for NPM-ALK-driven T cell transformation^8^, whereas STAT5B is important. Despite their shared tumorigenic potential^57,58^, STAT5 and STAT3 show opposing roles, for example, in dendritic cells^59^. Unlike STAT3, STAT5B promotes cytotoxic effector molecules typically found in mature T cells, such as perforin and granzymes. STAT5B may also drive transdifferentiation, potentially allowing ALCL clones to evade thymic negative selection, similar to mechanisms in T-ALL/T-LBL^33^. Indeed, over 50% of immature T-ALL cases rely on a hyperactive IL7RA-JAK1-STAT5B axis, which induces mature-like programs promoting infiltration^33^.

The cell of origin of ALCL has been debated over many years^23,24,60^. Our data implicate the DN2/3 stage to represent the tumor-initiating population. This is supported by transplantation of CD26^high^ DN cells, pseudotime trajectory analysis, reference-guided cell identity inference and marker gene expression patterns in murine and human samples. Our findings align with the model by Malcolm et al.^24^ in which NPM-ALK bypasses β-selection to generate CD4^+/-^/CD8^+/-^ cells, lacking productive TCR signaling^24^. These malignant CD4^+/-^/CD8^+/-^ cells, although phenotypically mature, retain transcriptional hallmarks of immature progenitors, suggesting a form of transdifferentiation or developmental mimicry, as shown in T-ALL^33^.

In NPM-ALK^Tg/+^ mice, STAT5B activity peaks at the DN2/3 stage, marked by high IL7R, pSTAT5, and STAT5 target gene activation. At this stage, IL2RA expression was reduced and IL2 stimulation failed to induce pSTAT5 in early transforming tumors. Conversely, *Cdk6*/*Stat5b*-deficient thymocytes showed reduced IL7R, elevated IL2RA and enriched IL2-STAT5 signatures. IL2 signaling, known to support ALCL maintenance^61^, is further upregulated in CDK6-deficient murine ALK^+^ cell lines, suggesting a cytokine switch in response to loss of IL7-STAT5B signaling.

We uncovered a developmental shift in *Cdk6*/*Stat5b*-deficient mice, accompanied by an early transition into DN3a and a partial DN2 block, along with altered expression of genes involved in differentiation, cell adhesion and chromosome organization. Several key chromatin regulators, including EZH2 and KDM5B, were direct CDK6 and STAT5B targets, underscoring a critical role for chromatin remodeling in thymic development^62^ and oncogenic transformation^63^. This mirrors findings from *Ezh2* knockout models, where thymocyte development is stalled at DN2/3 due to defective pre-TCR signaling, despite intact VDJ rearrangement^46^, suggesting that precise chromatin regulation is required for ALCL transformation.

We detected rearranged β-, γ-, and δ-TCR chains in ALK+ murine cell lines, indicating transformation occurs during ongoing TCR rearrangement. Similar patterns in patient ALCLs^24^ support a developmental mimicry model.

Reciprocal pathway activation upon deletion of either *Cdk6* or *Stat5b* suggests functional compensation and signaling network plasticity. This may explain the limited efficacy of single-agent targeting. However, combined deletion prevents clonal outgrowth in human ALCL cell lines, uncovering a context dependent synthetic lethal interaction.

ChIP-seq revealed a strong overlap of CDK6 and STAT5B promoter binding sites, suggesting cooperative or antagonistic transcriptional regulation. We identified a CDK6-STAT5B interaction in NPM-ALK^+^ cells involving STAT5B, consistent with a nuclear complex with potential regulatory activity. Supporting this, cells at the DN2/3 thymic stage lacking both CDK6 and STAT5B displayed an increased number of upregulated genes compared to controls, suggesting also a repressive role for CDK6 and STAT5B in regulating gene expression, either independently or as a complex. One example is *Kdm5b*, a histone demethylase with a CDK6/STAT5B double peak at its promoter, earlier activated in the *NPM-ALK*^Tg/+^*Cdk6*^*−/−*^*Stat5b*^*−/−*^ cells. This suggests CDK6 and STAT5B normally restrain *Kdm5b* to ensure stage-specific expression, and that its upregulation reflects a decoupling from tightly regulated developmental control.

The CDK6 core interactome in ALCL showed strong interactions with proteins involved in RNA polymerase II preinitiation complex (PIC) assembly, transcription preinitiation complex assembly, protein–DNA complex assembly, and the TFIID complex. Palbociclib treatment did not substantially alter interactions among these core partners, consistent with our previous findings, and indicates that kinase-inactive CDK6 not only binds DNA loci similar to wild-type CDK6^64,65^, but also participates in comparable transcriptional complexes.

Moreover, we identified a CDK6/STAT5B-driven transcriptional program that is active during early transformation and correlates with poor overall survival in patients. This gene signature may define a high-risk subgroup in which CDK6/STAT5B-driven circuitry is active and opens a therapeutic window.

Our study establishes a critical role for STAT5B and CDK6 in ALK⁺ALCL. While STAT3 controls classical ALK-mediated transcriptional programs, STAT5B confers transformation capacity. CDK6, beyond its role in differentiation, functions as a transcriptional amplifier that supports STAT5-dependent oncogenic reprogramming. Their interplay enforces a malignant identity in developing thymocytes. Gene signatures associated with CDK6/STAT5B loss correlate with improved survival in ALK⁺ ALCL patients, indicating their dual role in transformation and clinical outcome.

We uncover a cooperation of CDK6 and STAT5B in ALCL, where combined loss fully blocks tumorigenesis. We identify a disease-initiating cell population, in which the absence of CDK6 and STAT5B disrupts key transcriptional programs and alters thymic differentiation. The two genes showed a synthetic lethal interaction in a human ALCL model, with a CDK6/STAT5B associated gene signature stratifying patients with distinct overall survival outcomes, highlighting therapeutic opportunities.

While our study provides critical insights into the cooperative role of CDK6 and STAT5B during NPM-ALK-driven transformation, we would like to mention some limitations. First, the transgenic NPM-ALK mouse model^34^, although highly informative, does not fully recapitulate the peripheral aspects of human ALK^+^ ALCL^24^. Transformation in our model occurs exclusively within the thymus, reflecting an intrathymic developmental process. However, recent studies have suggested a thymic origin at least for a subset of human ALCL^24–26^, supported by mediastinal involvement in 25-45% of pediatric and adolescent cases^1^. This model, therefore, provided an ideal experimental setting to dissect the earliest oncogenic events within a physiologically relevant thymic microenvironment. In addition, the murine tumors closely resembled human ALK^+^ ALCL in several key characteristics, including comparable heterogeneity in surface marker expression in end-stage tumors^27,35^, non-productive TCR rearrangements^24^, consistent with an immature origin and conserved transcriptional programs^24,26,66–68^. A second limitation is the absence of conditional alleles for CDK6 and STAT5B which would have allowed the exclusion of potential microenvironmental influences from the outset. We addressed that concern by demonstrating that the effect of CDK6 and STAT5B KO is cell intrinsic. Finally, there are currently only few datasets from ALCL patients available that have consistent gene expression and survival data. The cohort in which we performed survival analysis was therefore relatively small, although the observed associations were robust and biologically consistent. Analyses in larger cohorts would be desirable.

## Methods

### Ethics statement

All breeding pairs and planned experiments are approved by the Ethics and Animal Welfare Committee of the University of Veterinary Medicine, Vienna in accordance with the University’s guidelines for Good Scientific Practice and authorized by the Austrian Federal Ministry of Education, Science and Research in accordance with current legislation, under licenses BMBWF-68.205/0174-V/3b/2018, BMWFW-68.205/0093-WF/V/3b/2015, 2022-0.404.452, BMBWF-68.205/0094-WF/V/3b/2015, 2024-0.826.297.

Animal welfare was monitored daily by assessing food intake, locomotion, behaviour, appearance, and body weight according to a predefined scoring system. The overall score determined the frequency of health checks, ranging from daily to twice-daily assessments, and defined when humane endpoints were reached. Mice meeting the predefined endpoint criteria were immediately euthanized.

### Mouse crossing and maintenance

*NPM-ALK*^*Tg/+*^ mice (Tg(Cd4-NPM/ALK)^N1Ingh^)^34^ and previously described *NPM-ALK*^*Tg/+*^*Cdk6*^*−/−*^ *mice* (Tg(Cd4-NPM/ALK)^N1ingh^Cdk6^tm1Bbd^)^28^ were crossed with *Stat5b*^*−/−*^ mice (Stat5b^tmHwd^)^69^ to generate *NPM-ALK*^*Tg/+*^*Stat5b*^*−/−*^ and *NPM-ALK*^*Tg/+*^*Cdk6*^*−/−*^*Stat5b*^*−/−*^ mice. Genotypes were confirmed by PCR analysis. All mice, including NSG mice (NOD.Cg-Prkdc^scid^ Il2rgtm^1Wjl^/SzJ; The Jackson Laboratory) were maintained at the rodent facility of the University of Veterinary Medicine Vienna. Specific pathogen-free (SPF) conditions were frequently confirmed according to FELASA recommendations for health monitoring^70^. All strains were maintained on a C57BL/6 background. Both male and female mice were used. No sex-specific effects were observed or expected and data from both sexes were combined for analysis. The light cycle (12-hour light period) and the barn climate parameters (temperature: 22 °C ± 1; humidity: 50% ±10) were centrally controlled and monitored.

### Murine cell line generation and maintenance

Upon reaching endpoint criteria, thymic lymphomas of *NPM-ALK*^*Tg/+*^*, NPM-ALK*^*Tg/+*^*Cdk6*^*−/−*^ and *NPM-ALK*^*Tg/+*^*Stat5b*^*−/−*^ mice were processed into single-cell suspensions using 40–70 µm strainers. Cells were cultured in RPMI 1640 (Sigma) medium supplemented with 10% heat inactivated fetal calf serum (Gibco, Fisher Scientific), 50 µM β-mercaptoethanol (Sigma), and 100 U/mL penicillin, 100 µg/mL streptomycin (Sigma) at 37 °C and 5% CO_2_. Cells were monitored microscopically until outgrowth and stable proliferation and were then maintained under these conditions for all subsequent experiments.

### Human cell lines

SU-DHL-1, DEL, SUP-M2, Karpas-299, SR-786, KI-JK, Mac-1 and Mac-2A cell lines were cultured in complete RPMI: RPMI 1640 (Sigma) medium supplemented with 10% heat inactivated fetal calf serum (Gibco, Fisher Scientific), 50 µM β-mercaptoethanol (Sigma), and 100 U/mL penicillin, 100 µg/mL streptomycin (Sigma) at 37 °C and 5% CO_2_. SUP-M2 cells were a kind gift from Olaf Merkel (AKH, Vienna), SU-DHL-1, Mac-1, and Mac-2A were provided by Richard Moriggl, and DEL and KI-JK cells were obtained from DSMZ.

### Colony formation assay

Viral supernatants were generated by transfecting Plat-E cells with a pMSCV-NPM-ALK-IRES-GFP vector using Turbofect (Thermo Fisher Scientific) in serum-free Dulbecco’s Modified Eagle Medium (DMEM). After overnight incubation, the media was replaced with complete RPMI and collected at 24 h and 48 h post transfection. Supernatants were filtered (0.45 µm) and supplemented with recombinant murine IL-3 (R&D Systems), IL-6 (Preprotech), IL7 (StemCell Technologies), all to a final concentration of 10 ng/mL, and stem cell factor (SCF, 1:60, Preprotech). Murine bone marrow cells were recovered by crushing femora and tibiae in PBS (phosphate buffered saline) using a mortar and pestle. Single-cell suspensions were obtained using a 70 µm cell-strainer. The cells were then spin-infected twice on consecutive days with viral supernatant in 6-well plates (2.5 mL viral supernatant + 1:500 Polybrene per 4*10^6^ cells in 500 µL RPMI). The plates were centrifuged at 600 g for 45 min. 24 h after the second infection viral supernatant was removed and cells were resuspended in complete RPMI without cytokines. 2*10^3^ GFP+ cells were then seeded in 2.5 mL growth factor-free methylcellulose (StemCell Technologies) in 35 mm dishes (Corning). GFP+ colonies were counted by fluorescence microscopy after 3 weeks, and GFP+ cell numbers were consequently counted by flow cytometry.

### Single-cell RNA sequencing

FACS-sorted thymic DN T cells from WT, *NPM-ALK*^*Tg/+*^ and *NPM-ALK*^*Tg/+*^Cdk6^−/−^Stat5b^−/−^ mice (*n* = 3 female mice, aged 10 weeks (69–72 days)) were subjected to library preparation for scRNA-seq using technology from 10x Genomics according to the manufacturer’s instructions. Data were quality controlled, batch corrected and visualized by UMAP. Thymocyte populations were annotated, revealing a tumor cluster in the *NPM-ALK*^*Tg/+*^ sample, which was further subclustered. Trajectory analysis was performed to study thymocyte and tumor development. The bioinformatic data analysis is described in detail in the supplementary information file.

### Cell type annotation

Library size normalization was performed per sample using the “scuttle” R package and the functions librarySizeFactors() with default parameters followed by logNormCounts() with the *transform* argument set to “log”. To annotate each cell by its cell type we made use of bulk gene expression data from mouse immune cells, generated as part of the “Immunologic Genome project” and provided by the “celldex” 1.0.0 R package using the ImmGenData() function. This dataset consisted of 253 cell types that were used to annotate our in-house single cell data using the “SingleR” package with the following function: SingleR(test = sce, ref = ref, assay.type.test = “logcounts”, labels = ref$label.fine) where sce is our in-house dataset and ref constitutes the ImmGen data.

### Cluster marker genes

To calculate cluster marker genes in the *NPM-ALK*^Tg/+^ sample we first calculated log transformed normalized gene expression values. We used the “scuttle” package’s librarySizeFactors() function with default parameters, followed by the logNormCounts() functions with the *transform* argument set to “log”. To calculate marker scores for each gene in each cluster, we used the scoreMarkers() function from the scran package with the following parameters: the cluster information was passed to the *group* argument, *assay.type* was set to “logcounts”, and *lfc* was set to 0.67. Cluster markers with a median AUC above 0.65 were selected for unsupervised clustering to identify tumor cluster markers.

### Integration of the *NPM-ALK*^Tg/+^ scRNA-seq data with mouse whole thymus data

Murine Thymocyte scRNA-seq data from two male and two female C57Bl/6 J mice aged 11-13 weeks were downloaded from the Broad Institute Single Cell portal (https://singlecell.broadinstitute.org; study ID: SCP1833; downloaded on 19.02.2025). Seurat 4.0.1 was used to analyze the data starting from the processed barcodes, features and matrix files. A Seurat object was generated using the Read10X() and CreateSeuratObject() functions. The data were then processed by executing the following functions: NormalizeData(), FindVariableFeatures() (setting the *selection.method* argument to “vst” and *nfeatures* to 2000), ScaleData(), RunPCA(), RunUMAP() (with *dims* set to 1:30), FindNeighbors() (with the following arguments: *dims* = 1:30, *reduction* = “pca”, *k.param* = 7) and FindClusters() with a *resolution* of 0.6.

We next set out to annotate each cell by its cell type. To do that, a copy of the mouse thymocyte Seurat object was generated in SingleCellExperiment format. Then we used the ImmGen dataset to annotate the mouse thymocytes as described above for the annotation of our in-house single cell data. Here, the SingleR() function argument *test* constituted the murine thymocyte data and *ref* constituted the ImmGen data. The resulting cell labels were slightly simplified (e.g. all B cell and all CD4 T cell labels were renamed to “B cell” and “CD4”, respectively) and then added to the murine thymocyte Seurat object.

Next, this annotated mouse thymocyte object was integrated with our in-house *NPM-ALK*^Tg/+^ data using Seurat. The *NPM-ALK*^Tg/+^ object was transformed to the Seurat format, and the data were normalized using the NormalizeData() function. 2500 integration anchors were identified using FindIntegrationAnchors() with the mouse thymocyte dataset as the reference. These anchors were then passed to the IntegrateData() function. The resulting integrated dataset was scaled (ScaleData()) and PCA and UMAP were calculated using the RunPCA() and RunUMAP() (based on the first 30 PCA dimensions) functions, respectively.

To infer the closest related cell types of tumor cells within the whole thymus scRNA-seq we performed a label transfer analysis using Seurat 4.0.1. For this, clusters tumor1-6 of the *NPM-ALK*^Tg/+^ were mapped to the whole thymus. These six clusters were pre-processed using NormalizeData() and FindVariableFeatures() with the “vst” *selection.method*. With the FindTransferAnchors() function, transfer anchors were calculated using the whole thymus as the reference and the tumor clusters as the *query*. The dims were set to 1:30 and with TransferData() the anchorset was used to transfer cluster labels of the *reference* set to the tumor clusters.

### Differentially expressed patient ALCL markers

To identify tumor marker genes within our scRNA-seq that are also marker genes in patients, we performed differential gene expression analysis and marker gene analysis across multiple publicly available transcriptomic datasets.

Four datasets were downloaded using https://www.ncbi.nlm.nih.gov/geo/geo2r/ on 13.01.2025. Within these 4 datasets, we calculated the following comparisons: GSE14879 5 ALK^+^ALCL patients vs. lines vs. 41 controls (other cell types), GSE14879 3 ALCL cell lines vs. 41 controls (other cell types), GSE19069 20 ALK ALCL vs 10 controls (other T cells), GSE65823 23 ALCL vs. 8 PTCL, GSE78513 23 ALCL vs 12 reactive lymph nodes. All these datasets were analyzed with default settings, applying the Benjamini-Hochberg option for *p*-value calculation and downloaded. Genes with an adjusted *p* value below 0.1 and an absolute fold change above 1.25 were considered marker genes. In the case of multiple probes, only the probe with the lowest adjusted *p*-value was considered.

Additionally, scRNA-seq data (fastq files) generated from lymph nodes of two human ALK^+^ALCL patients were downloaded from the Sequence Read Archive (SRA) using the SRA toolkit according to the programmer’s instructions (Run accessions: SRR24860075 and SRR24860077, downloaded on 06.05.2024 and 08.05.2024, respectively). The data were processed using the CellRanger v.7.0.0 “count” program with the GRCh38-2024-A pre-built reference transcriptome (10x Genomics) to generate raw and filtered feature-barcode-matrix data. Further analysis was done in R with the “Seurat” package. The matrices were imported, calling the Read10X() and CreateSeuratObject() functions, allowing only cells with a minimum of 200 features to be included. Quality assessment and filtering were done by first calculating the percent mitochondrial UMIs per cell with the PercentageFeatureSet() function. Next, we manually inspected distributions of percent mitochondrial UMIs, total UMI counts and feature counts. We decided to keep cells that had more than 1000 and less than 15,000 total UMIs and more than 1% and less than 20% mitochondrial UMIs in sample SRR24860075. The filtering cutoffs for cells in sample SRR24860077 were more than 2000 and less than 50000 total UMIs and more than 2% and less than 25% mitochondrial UMIs. Next, the data were transformed using SCTransform() with *vars.to.regress* set to the mitochondrial UMI percentage. On the transformed data we performed dimensionality reduction with RunPCA() and RunUMAP() (the latter on the first 30 PCs). We called FindNeighbors() setting the following arguments: *dims* = 1:30, *reduction* = “pca”, *k.param* = 7. We then identified clusters using FindClusters() with the *resolution* argument set to 0.3. Tumor marker genes were detected by selecting the cluster with the highest ALK expression. Genes with an adjusted *p*-value below 0.01 and an average log_2_ fold-change above 1.5 (for SRR24860075) or 1 (for SRR24860077) were considered as marker genes. The last downloaded dataset was downloaded from https://www.ebi.ac.uk/gxa/experiments/E-MTAB-2770/Results on 13.01.2025 and comprised of TPM values from cell line RNA-seq data. We compared all ALK^+^ALCL cell lines with all hematopoietic cell lines within this set. Genes were defined as ALK^+^ALCL specific if they showed mean TPM > 5 in ALCL lines and a log_2_ fold-change above 1.

### Bulk RNA sequencing from mouse and human ALCL cell lines

Cell lines were cultured as described above before RNA was isolated using the Qiagen RNeasy Mini kit according to the manufacturer’s protocol. Libraries for RNA sequencing using Illumina technology were prepared at the Vienna Biocenter NGS core facility using the NEBNext Ultra II stranded poly-A library prep kit (New England Biolabs, Ipswich, MA, USA). Libraries were sequenced on an Illumina NovaSeq 6000 system (Illumina, San Diego, CA, USA) in paired-end mode with 50 bp read length.

### Bulk RNA sequencing datasets from ALCL patients

Human RNA-seq data (fastq files) from 38 ALCL patients were downloaded from the Genome Sequence Archive for human (https://ngdc.cncb.ac.cn/gsa-human/, accession HRA004246) via ftp://human.big.ac.cn on 20.12.2024 (ALCL patient set1). Human RNA-seq data (fastq files) from 23 ALCL patients (European Nucleotide Archive project ID: PRJNA255877) were downloaded via ftp://ftp.sra.ebi.ac.uk/ on 17.01.2025 (ALCL patient set2).

### Bulk RNA sequencing analysis

Raw sequencing reads were quality controlled using FastQC (version 0.12.1). Adapters and low-quality reads were trimmed using Trimmomatic (version 0.39) and the resulting reads were again quality controlled with FastQC. The processed reads were aligned against reference genomes using STAR (version 2.7.6a). For mouse samples, the *GRCm38 primary assembly* reference genome (Gencode vM25) was used together with the *Gencode vM25 primary assembly annotation* gene model. Human samples were aligned against the *GRCh38 primary assembly* reference genome (Gencode v26). The gene model used for the human data was the *Gencode V26 primary assembly annotation*. QC of the alignments was done with Qualimap (version 2.2.2a). Finally, reads overlapping exons were counted with the FeatureCounts program of the Subread package (version 2.0.1) and summarized as total counts per gene.

Differential gene expression analysis was performed in R (version 4.3.1) using the DESeq2 (version 1.40.2) package. Genes with less than 10 counts in total (sum over all samples) were excluded from further analysis. The gene expression levels of the remaining genes were then modeled as a function of sample genotype, which was coded as a factor variable with three levels (*NPM-ALK*^Tg/+^*Cdk6*^*−/−*^, *NPM-ALK*^Tg/+^*Stat5b*^*−/−*^ and WT; the latter being used as the reference level). The DESeq() function was called to perform the analysis and the results() function was used to extract analysis results comparing *NPM-ALK*^Tg/+^*Cdk6*^*−/−*^ vs. WT and *NPM-ALK*^Tg/+^*Stat5b*^*−/−*^ vs. WT samples.

### Pathway heatmap analysis

Normalized RNA-seq expression data were obtained from four RNAseq datasets described above. Expression matrices were processed in R (v4.3.1). Genes belonging to the JAK-STAT (KEGG), MAPK (Biocarta), and PI3K-AKT-mTOR (Hallmark) were identified from MSigdb databases. Gene expression matrices were filtered to include only genes which were found expressed in all four datasets. Log2 transformation with a pseudocount of 1 was applied, followed by min-max- normalization per sample to allow cross -sample comparison. The heatmap was plotted using pheatmap.

### Trajectory analysis

Trajectory analysis was performed with the scRNA-seq data of the *NPM-ALK*^Tg/+^ sample. Normalized expression values were calculated using the computeLibraryFactors() and logNormCounts() functions from the “scuttle” R package. To identify the 10% most variable genes, we used the modelGeneVarByPoisson() and the getTopHVGs() (with the *prop* argument set to 0.1) functions form the “scran” R package. PCA was performed based on these highly variable genes using runPCA() from the “scater” package. We then used the TSCAN package to calculate a minimum spanning tree through the cluster centroids. We used the createClusterMST() function with the following arguments: *x* was the *NPM-ALK*^Tg/+^ SingleCellExperiment object, *clusters* was set to the clusters defined above, *use.dimred* was set to the PCA name, *dist.method* was “simple”, outgroup was “TRUE” and no *endpoints* were defined. In order to visualize the minimum spanning tree on the UMAP, we calculated the UMAP coordinates of the cluster centroids using the aggregateAcrossCells() function from the “scuttle” package. We then used TSCAN’s reportEdges() function (passing on the aggregated object and setting *mst* to the result of createClusterMST(), *clusters* to NULL and *use.dimred* to the UMAP name) to retrieve edge information and store it in an object. These edges were added to the UMAP plot generated with plotReducedDim() by adding a line layer as follows: geom_line(data = <edge-object > , mapping=aes(x=dim1, y=dim2, group=edge)).

### Label transfer

The scRNA-seq data from the *NPM-ALK*^Tg/+^ sample was split into two datasets. First, the cells that were attributed to clusters which were also identified in the WT sample made up the “normal” subset. Second, the cells that were annotated to one of the tumor clusters made up the “tumor” subset. Seurat was then used to perform label transfer. The “normal” dataset was pre-processed by calling the following functions: NormalizeData(), FindVariableFeatures() (setting the *selection.method* argument to “vst”), ScaleData(), RunPCA(), RunUMAP() (with *dims* set to 1:50). The “tumor” sample was pre-processed using NormalizeData() and FindVariableFeatures() with the “vst” *selection.method*. Transfer anchors were then calculated using the FindTransferAnchors() function with the “normal” sample as *reference* and the “tumor” sample as *query*. The *dims* argument was set to 1:30. The resulting anchor set was used with the TransferData() function to transfer cluster labels from the “normal” sample to the cells in the “tumor” sample. To assess the similarity of the tumor cells with cells from both other genotypes (WT and *NPM-ALK*^*Tg/+*^*Cdk6*^*−/−*^*Stat5b*^*−/−*^), the analysis was repeated in a corresponding fashion. Only clusters with more than 20 assigned cells were plotted. All other methods used in this study are described in the supplementary information file.

### Reporting summary

Further information on research design is available in the Nature Portfolio Reporting Summary linked to this article.

## Supplementary information

Supplementary Information

Reporting Summary

Transparent Peer Review file

## Source data

Source Data

## Data Availability

Murine scRNA-seq data generated in this study have been uploaded to ArrayExpress under accession: E-MTAB-15478. Human and murine ChIP-seq data generated in this study are available under E-MTAB-15481, and human and murine bulk RNA-seq data generated in this study under E-MTAB-15486. Publicly available datasets analyzed in this study include microarray data (GSE14879 [https://www.ncbi.nlm.nih.gov/geo/query/acc.cgi?acc=GSE14879], GSE19069, GSE65823, GSE78513), human ALCL scRNA-seq (SRR24860075, SRR24860077 [https://www.ebi.ac.uk/ena/browser/view/PRJNA980417]), RNA-seq from ALCL patients (E-MTAB-2770 [https://www.ebi.ac.uk/biostudies/arrayexpress/studies/E-MTAB-2770], HRA004246, PRJNA255877), DN ChIP-seq data (GSE283392 [https://www.ncbi.nlm.nih.gov/geo/query/acc.cgi?acc=GSE283392]), human H3K27ac ChIP-seq data (GSE158916 [https://www.ncbi.nlm.nih.gov/geo/query/acc.cgi?acc=GSE158916]) and survival and gene expression data (GSE58445 [https://www.ncbi.nlm.nih.gov/geo/query/acc.cgi?acc=GSE58445], GSE19069, E-TABM-117). Source data are provided with this paper.
