# Supplementary material for: Ablation of CDK6 and STAT5B rewires T cell developmental dynamics and prevents ALK+ lymphoma formation
Source: Nat Commun. 2026 Sep 15;17:10853. doi: 10.1038/s41467-026-77466-4 (PMC13672290; doi:10.1038/s41467-026-77466-4)
Supplement: Supplementary file 1 [file 41467_2026_77466_MOESM1_ESM.pdf]

Supplementary Information

Contents

- Supplementary Methods
- References
- Supplementary figures
- Supplementary figure legends

Supplementary Methods

Analysis of single-cell RNA sequencing datasets

Single-cell RNA sequencing raw data were processed using the CellRanger v. 7.0.0 “count” program to generate raw feature-barcode-matrix data. Further analysis was performed in R. The “DropletUtils” package was used to import the cellRanger results using the read10xCounts() function. After removing duplicate genes (by gene symbols) emptyDrops() was used to identify empty droplets to be removed. QC metrics were calculated using the “scater” package’s perCellQCMetrics() function. Among total UMI counts per cell we also calculated the percentage of mitochondrial counts by providing a list of mitochondrial genes. We then selected cells that fulfilled the following requirements for further analysis: mitochondrial percentage >= 1 and mitochondrial percentage <= 6 and log2(total UMI count) >= 11 and log2(total UMI count) <= 14.5. Batch correction was then performed with the quickCorrect() function of the “batchelor” package, setting the following arguments: *correct.all* = TRUE, *assay.type* = "counts", *PARAM* = FastMnnParam(BSPARAM=BiocSingular::RandomParam(), *auto.merge*=TRUE. The quickCorrect() function returns low-dimensional corrected coordinates for every cell. The first 30 of these dimensions were used to perform uniform manifold approximation and projection (UMAP) analysis. We set a seed 1100101001 (R function set.seed()) and used the umap() function from the “uwot” package with default arguments, except for *spread* which was set to 2, *min_dist* which was set to 0.15 and *ret_model* was TRUE.

We next performed clustering of cells using the clusterRows() function from the “bluster” package. A seed was set to 650476. Again, the first 30 dimensions of the corrected matrix were used. The clustering algorithm function used was SNNGraphParam() with *k* set to 3, *cluster.fun* set to “louvain” and *cluster.args*

set to a resolution of 1.1. We identified a distinct group of tumor cells in the *NPM-ALK*$^{Tg/+}$ sample as described in the results and decided to further subcluster these cells. To do that, we first subsetted the SingleCellExperiment object to only cells from the NPM-ALK$^{Tg/+}$ sample. Next, we set out to perform dimensionality reduction as follows: We calculated normalized counts using the "scuttle" packages computeLibraryFactors() and logNormCounts() functions, setting *center.size.factors* to TRUE. We identified highly variable genes with the "scran" package and the modelGeneVar() function, followed by calling getTopHVGs(), setting the *prop* argument to 0.1. PCA was then done with these highly variable genes using runPCA() from the "scater" package. The resulting object was then subsetted for the tumor cells. Clustering of these cells was then performed as described above, with the exception that it was based on the newly generated principal components, that we used *k* = 20 nearest neighbors, and a resolution of 0.5. The used seed was 800747. Finally, we replaced the original cluster identities of the tumor cells with the newly generated tumor subclusters.

Flow cytometry

Murine thymuses were harvested and strained through a sterile 40 µm filter. To block Fc receptors, purified anti-CD16/CD32 antibodies (clone 93; Thermo Fisher Scientific (eBioscience™)) were used. Fluorochrome-conjugated antibodies for the detection of the following proteins, also for surface marker scrrenings in human and murine cell lines, were used from Biozym/BioLegend: CD4 BV650 (GK1.5), CD8a BV606 (53-6.7), CD26 APC (H194-112), CD44 PerCP (IM7), Gr-1 APC-Cy7 (RB6-8C5), TER-119 APC-Cy7 (TER-11), CD26 PE (H194-112), CD274 APC (B7-H1, PD-L1; 29E.2A3), CD54 PE (HA58), CD26 PE (BA5b), CD107a PE (H4A3), HLA-A,B,C PE (W6/32), CD135 PE (BV10A4H2), CD11b APC (ICRF44), CD90 PE-Cy5 (5E10), KLRG1 APC (2F1/KLRG1), CD24 FITC (ML5), CD120b APC (3G7A02), CD120a APC (W15099A), CD120a APC (55R-286), CD38 PE-Cy7 (HB-7), CD34 PE (581), CD48 PE (HM48-1), CD274 BV421 (10F.9G2), LY49A PE (A1/Ly49A), TCRB APC (H57-597), CD90.1 APC (OX-7/OX7), CD127 Alexa Fluor488 (A7R34), Clone: CD3 PB (17A2), CD226 APC (10E5), CD326 APC-Cy7 (G8.8), CD94 APC (18d3), CD126 APC (D7715A7), TCRGD BV605 (GL3), Streptavidin PECy7 (1:1000), Thy1.2 BV510 (53-2.1); from BD Biosciences: CD19 FITC (1D3), CD11B FITC (M1/70), Alexa Fluor647 CD61(2C9.G2 (HMß3-1)), CD11b APC-Cy7 (M1/70), CD25 PE-Cy7 (PC61), CD19 APC-Cy7 (1D3),

CD24 FITC (M1/69), CD63 FITC (H5C6), CD43 PE (S7), CD62L APC-Cy7 (MEL14), SCA1 PE-Cy7 (D7), CD5 PE (53-7.3), CD31 FITC (MEC13.3), CD34 FITC (RAM34), CD19 PE (1D3), LY6G/C FITC (RB6-8C5) CD107a FITC (1D4B), TCRA FITC (B20.1), CD4 FITC (GK1.5), CD335 FITC (29A1.4), CD44 APC-Cy7 (IM7), pSTAT5-PB450 (47/Stat5(pY694) (1:6)), Ki-67 FITC (1:10; B56), CD25 PE (3C7), CD8 APC (53-6.7); from eBioscience/FisherScientific: CD44-PE (IM7), CD127 (IL7R eFluor450 (A7R34)), CD9 FITC (SN4 C3-3A2), CD45 FITC (HI30), MHC Class I (H-2Db) PE (28-14-8), MHC Class II (I-A/I-E) FITC (M5/114.15.2), CD61 PE (2C9.G3), CD16/32 PE (93), CD140b PE (93), CD244.2 PE (eBio244F4), CD117 APC (2B8), CD51 PE (RMV-7), CD150 FITC (9D1), CD123 PE (5B11), CD253 PE (N2B2), CD135 PE (A2F10), CD215 APC (DNT15Ra), CD49b APC-Cy7 (DX5), NK1.1 eFluor 450 (PK136), CD197 eFluor450 (4B12), CD122 FITC (TM-b1), CD127 FITC (A7R34), purified anti-CD16/CD32 antibodies (clone 93); and from Invitrogen/FisherScientific: CD25 Biotin (7D49), GR1 FITC (RB6-8C5), IgG (H+L) Cross-Adsorbed Secondary Antibody Alexa Fluor647 (1:500), CD45 FITC (30-F11), CD71 FITC (R17217), CD9 eFluor450 (KMC8), CD144 eFluor660 (eBioBV13), KLRG1 FITC (2F1), CD93 APC (AA4.1), CD314 APC (CX5), CD4 PE-Cy7 (GK1.5). If not stated otherwise for each experiment, flow cytometry antibodies were used at a 1:200 dilution.

Flow cytometry samples were acquired using a Cytoflex S (Beckman Coulter), Cytoflex (Beckman Coulter), Cytoflex SRT (Beckman Coulter), or a BD FACSAria™ III Cell Sorter (BD Biosciences) and then further analyzed using CytExpert (Version 2.4.0.28, Beckman Coulter), CytExpert SRT (Version 1.1.0.10007, Beckman Coulter), or FlowJo (Version 10.8.-10.10.). Viability gating was validated in a secondary experiment employing a viability dye to ensure accuracy given the complexity of the staining panels.

Intracellular stainings

Intracellular CDK6 or pSTAT5 stainings were performed as described previously[1]. *Ex vivo* murine samples for pStat5 stainings were stimulated in complete RPMI using either 3000 U/mL human IL2 (Proleukin,SteriMax) or IL7 (50 ng/mL) for 20 min and compared with unstimulated samples. Surface panels for pSTAT5 stainings included the following antibodies: CD4 BV650, CD8 BV605, CD44 PE, CD25 Biotin, CD26 APC, lineage: (CD19 FITC, GR1 FITC, CD11B FITC) and for CDK6 stainings: CD4 BV650, CD8 FITC, CD44 PE, CD25 Biotin. Surface-stained cells were fixed with 2% parafolmaldehyde (Sigma-

Aldrich) in PBS at 37°C for 10 min, then permeabilized with 90% ice-cold methanol in PBS containing 2% FCS and 0.2% Tween-20 for 30 min at 4°C. All subsequent washes were performed using PBS containing 2% FCS and 0.2% Tween-20. For pSTAT5 stainings, cells were stained with pSTAT5-PB450 (47/Stat5(pY694); BD Biosciences) and Streptavidin-PECy7 antibodies on ice for 90 min before the final washing steps. For CDK6 stainings, the CDK6 antibody (rabbit polyclonal, PA5-27978; Invitrogen, USA; used at a 1:250 dilution) was added first and incubated at room temperature for 60 min before washing and incubating with IgG APC (H+L) and streptavidin PECy7 on ice for 30 min before final washing steps and subsequent analysis.

Pseudotime analysis and gene expression clusters

To determine cells limited to the exact tumor trajectory, we made use of a pseudotime analysis and started by only investigating the right side (this includes the earliest stages of T cell differentiation which includes all clusters adjacent to the tumor trajectory) of the UMAP. Murine sc-RNAseq data were processed using the “SingleCellExperiment” framework. Cells from selected clusters were excluded based on prior annotations. On the remaining subset of cells, we performed PCA on reconstructed counts using scater::runPCA(). Graph-based clustering was performed using the “bluster” package (SNNGraphParam(k=25, cluster.fun =”infomap”)). Library size factors were recalculated per batch and used for log-normalization. Cell type annotation was performed using SingleR() with the Immgen reference celldex, and dendritic cells (ImmGen labels starting with “DC”) were removed. The resulting dataset was used to perform PCA on log-normalized counts using scater::runPCA(), separately for each sample. The top 50 PCs were retained. Minimum spanning trees (MSTs) were generated using the createClusterMST() function from “TSCAN”, with median PCA coordinates per cluster (aggregateAcrossCells(), *dimred.stats*=”median”) used as cluster centroids. In the *NPM-ALK*$^{Tg/+}$ sample, we constructed a pruned MST, connecting the tumor cluster to precursors, as all tumor trajectories utilized the same entrypoint into the tumor, and we wanted to focus on genotypical differences of cells before entering the tumor cluster. To infer pseudotime, single cells were first projected onto the MST backbone using mapCellsToEdges(), followed by ordering via orderCells(). Pseudotime matrices from all samples were harmonized by aligning their column structure, and a unified pseudotime matrix was assembled by row-binding all samples. To

identify dynamically regulated genes, testPseudotime() was applied to the entire object. We focused only on the cells that were associated with the direct tumor trajectory and divided the entire lineage into 10 equidistant intervals (distance between 2 points ~5.2 pseudotime units (red points)) (Fig. S3c). To evaluate clustering robustness across the 10-25 cluster range, silhouette plots were generated on a random set of 2000 genes. Highly variable genes (HVGs) were then identified using the modelGeneVar() function from the scran() package, selecting the top 2000 HVGs ranked by their biological variance. For each of those genes, the slope of expression change within each interval using linear regression (lm) was used. The resulting slope matrix was clustered using K-means (k=18, 100 iterations) to identify gene groups with shared pseudotime dynamics. For each gene, the mean expression per pseudotime-interval was also calculated and combined with the slope values (slope per interval*expression in interval*100) to determine an “activity score”. The average activity score per cluster was computed using dplyr::summarise() with default parameters.

Shift gene analysis

For each gene cluster, pseudotime dependent gene expression was smoothed using *LOESS* regression on logcount values. The smoothed curves were plotted separately for each genotype. To account for potential temporal misalignment between the *NPM-ALK*$^{Tg/+}$*Cdk6*$^{-/-}$*Stat5b*$^{-/-}$ and *NPM-ALK*$^{Tg/+}$ pseudotime trajectories, an automated pseudotime shift calculation was implemented. For each cluster, mean gene expression from *NPM-ALK*$^{Tg/+}$ and *NPM-ALK*$^{Tg/+}$*Cdk6*$^{-/-}$*Stat5b*$^{-/-}$ were compared in the pseudotime range between 13 and 22 units (which covers the differentiation from DN2B to DN3A cells). A grid search over pseudotime shifts (0-7 in 0.15 unit increments) was performed to identify the optimal shift minimizing the mean squared error (MSE) between *NPM-ALK*$^{Tg/+}$ and shifted *NPM-ALK*$^{Tg/+}$*Cdk6*$^{-/-}$*Stat5b*$^{-/-}$ expression curves. Both curves were interpolated over 1000 evenly spaced pseudotime points, and MSE was computed between the *LOESS* predictions. The optimized shift was applied to *NPM-ALK*$^{Tg/+}$*Cdk6*$^{-/-}$*Stat5b*$^{-/-}$ pseudotime values, and both the original and shifted *NPM-ALK*$^{Tg/+}$*Cdk6*$^{-/-}$*Stat5b*$^{-/-}$ trajectories were plotted for comparison. From the resulting 18 shifts, we focused only on the gene sets showing a shift larger than the median shift, resulting in 7 gene sets that were affected most by the shift.

For the shift analysis, which included CHIP peaks of CDK6, STAT5B or both (Peak classification described in the CHIPseq analysis section), peaks were intersected with genes belonging to the GOBP_CHROMATIN_ORGANIZATION.v2025.1.Mm dataset. Genes were categorized according to the presence of CDK6 and/or STAT5B occupancy in their promoter region (range around the TSS ranging from -300 to 200 bp). The shift of every single gene was determined as described above.

Cell- type annotation and quantification across pseudotime

We performed time-resolved analysis across defined pseudotime intervals to determine dynamic changes in DN2B and DN3A T cell populations. Pseudotime was inferred using the prefiltered dataset as described above. For this analysis, a distance of two pseudotime units was selected. For each interval, cells were selected based on their pseudotime assignment and subsequently annotated using SingleR() with the ImmGen reference dataset, focusing on fine level annotations. Two cell types were included in downstream analysis: T cells (T.DN2B) and T cells (T.DN3A). For each pseudotime interval, we compared the relative abundance of DN2B and DN3A cells by calculating percentages based on the total number of annotated cells. This was done in each sample (WT, *NPM-ALK*$^{Tg/+}$ *and NPM-ALK*$^{Tg/+}$*Cdk6*$^{-/-}$*Stat5b*$^{-/-}$*)* separately. Smoothed plots displayed the *LOESS*- fitted cell type percentages along the pseudotime.

STRING Pathway Enrichment and Network Clustering

Pathway enrichment analysis using the Biological Processes (Gene Ontology) gene sets was performed using STRING (v12.0; https://string-db.org/, accessed 17.03.2025) based on the shifted gene list. The interaction network was analyzed on 07.08.2025 and was based on the shifted genes list with a STAT5B and/or a CDK6 peak in their promoter region after filtering for genes (364 genes) with a minimum interaction score of 0.7 (high confidence). Disconnected nodes were hidden to improve network clarity. For module detection, we made use of MCL clustering (inflation parameter: 1.5) and detected 24 clusters. Cluster composition and enriched pathways were further investigated using STRING's built-in functional enrichment tools.

Cell type annotation of DN2/3, ISP and γδ T cells in scRNA-seq

The cell type annotation using fine Immgen labels was performed as described above. To simplify cell type labels, T cell subsets, DN2B and DN3A were combined into a single category and other labels were reduced to the simplified labeling. Frequencies of DN2B/DN3A, γδ T and ISP cells were quantified across all clusters, excluding the tumor clusters, and stratified by genotype.

Dimensional reduction of Flow cytometry experiment

The UMAP visualization of thymic cell population from a clustering derived from 29 mice was done using FlowJo (v.10.10.0) with the following surface staining panel: lineage (Gr1 APC-Cy7, TER119 APC-Cy7, CD19 APC-Cy7, CD11b APC-Cy7), CD4 BV650, CD8 BV605, CD25 PE-Cy7, CD44 PerCP, CD26 PE, IL7R PB450, TCRβ APC, CD24 FITC. After downsampling, using the Plugin DownSample (v3.3.1), $1*10^4$ cells from lineage negative singlets of every sample were concatenated. UMAP display was generated using the plugin UMAP (v3.1)[2], and populations and surface marker expressions were investigated using FlowSOM (v3.0.18)[3].

Transplantation experiments

Thymic cells were obtained from mice at age 48-63d. Cells were handled as described above and stained with the following panel using the gating strategy in Fig. S2e: lineage (Gr1 APC-Cy7, TER119 APC-Cy7, CD19 APC-Cy7, CD11b APC-Cy7), CD4 BV650, CD8 BV605, CD25 PE-Cy7, CD44 PerCP, CD26 PE, IL7R FITC, CCR7 eFluor450 and CD61 Alexa Fluor647. $8*10^4$ cells from indicated populations were intravenously injected into NSG mice. In 3 of 4 cases, the indicated populations were pooled from two mice.

Apoptosis Staining Flow cytometry

Annexin V/DAPI (4',6-diamidino-2-phenylindole) staining of murine thymocytes was done according to the manufacturer’s protocol from the Annexin V Apoptosis Detection Kit (Thermo Fischer Scientific) with a few adjustments. In brief, cells were washed once with PBS and once with 500 µL 1x Binding Buffer. For the staining, FITC-conjugated Annexin V Antibody (1:20) (Biolegend/Biozym Scientific GmbH; Cat. 198364) was mixed with Binding Buffer and added to each tube. Samples were vortexed for 15 min at room temperature in the dark. After incubation, the cells were washed once with 1x Binding Buffer and subsequently resuspended in 100 µL of DAPI with a final concentration of 2 µg/mL. Surface staining was

performed using the following panel: lineage (Gr1 APC-Cy7, TER119 APC-Cy7, CD19 APC-Cy7, CD11b APC-Cy7), CD4 BV650, CD8 BV605, CD25 PECy7, CD44 PerCP, CD26 PE.

Cell cycle analysis by Flow Cytometry

Single cell suspensions from murine thymuses (40 µm filter) were Fc blocked (anti-CD16/CD32, Thermo Fisher) and surface stained for CD4 (PE-Cy7), CD8a (APC), CD25 (PE), CD44 (PerCP, IM7), Thy1.2 (BV510, 53-2.1), and a lineage panel (Gr-1, TER-119, CD19, CD11b; all APC-Cy7). Cells were fixed and permeabilized (BD Cytofix/Cytoperm, BD Biosciences) as adapted from Galvin et al. (2019)[4], stained intracellularly for Ki-67 (FITC, B56), and counterstained for DNA with DAPI (2 µg/mL, Sigma-Aldrich D9542). Gating proceeded from live cells (FSC/SSC) to singlets (DAPI area versus width), lineage negative (APC-Cy7 dump), and Thy1.2 positive cells. Double negative thymocytes (CD4−CD8−) were resolved by CD44 and CD25 into DN1 (CD44+CD25−), DN2 (CD44+CD25+), DN3 (CD44−CD25+), and DN4 (CD44−CD25−). Within each subset, Ki-67 versus DAPI defined G0 (Ki-67−, 2N), G1 (Ki-67+, 2N), and S-G2-M (Ki-67+, >2N).

Surfacemarker screens Flow cytometry

Single stains were done by using Fc-blocking antibodies (except for the analysis of CD16/CD32) and a subsequent incubation with the conjugated antibody of interest in the dark for 30 min at 4°C. Staining was performed separately for every cell line.

TCR chains rearrangements

Rearrangements of TCR β-, γ- ,δ-chains were assessed by PCR on genomic DNA using previously published primers and protocols[5,6]. All TCRβ chains were analyzed in a multiplex reaction, while γ- and δ-chains were amplified in individual PCRs.

Disuccinimidyl sulfoxide (DSSO) crosslinking

SU-DHL-1 cells were counted, and 20 million cells were starved in RPMI-1640 medium, 0% FCS for 20 min. Cells were then stimulated for 20 min in RPMI-1640 medium containing 20% FCS. After stimulation, cells were washed twice with ice-cold PBS and resuspended in 2 mL of ice-cold PBS. 1 mM DSSO (Thermo

Fisher Scientific, A33545) was added to the samples and incubated for 10 min on ice. The reaction was stopped by adding 20 mM Tris (tris(hydroxymethyl)aminomethane) buffer and incubating for 15 min on ice. The excess non-reacted crosslinker was removed from the cells by washing them two times with PBS.

Coimmunoprecipitation (co-IP)

SU-DHL-1 were counted, and 20 million cells were starved in RPMI-1640 medium, without FCS for 20 min. Cells were then stimulated for 20 min in RPMI-1640 medium containing 20% FCS. After stimulation, the cells were washed twice with ice-cold PBS and either directly lysed or subjected to DSSO crosslinking. Not crosslinked and crosslinked cell pellets were lysed in IP lysis buffer (50 mM Tris-HCL (pH 7.4), 150 mM NaCl, 1 mM EDTA, 1% Triton X-100, 10% glycerol, 1x protease inhibitor cocktail (Roche), 1 mM Na_3VO_4 and 10 mM NaF) on ice for 1 h with resuspension every 10 min. The samples were then centrifuged at 17000 g at 4°C for 5 min and the supernatants were obtained for protein concentration measurement. Protein concentration was measured colorimetrically (Pierce™ BCA Protein Assay Kit, Thermo Fisher Scientific) on a Varioskan™ LUX Multimode Reader and analyzed using the SkanIt™ software. 15 µg of total protein lysate was saved for the input fraction for western blot analysis. 250 µg of protein lysate was immunoprecipitated by incubating with 8 µg of a CDK6 antibody (rabbit polyclonal, PA5-27978; Invitrogen) at 4°C overnight under constant rotation in a total volume of 500 µL. As negative control, 250 µg of protein lysate was incubated with 8 µg of the rabbit IgG isotype control antibody (IgG-R; 02-6102, Invitrogen). Next, 25 µL of Pierce™ A/G Magnetic Beads (Ref 88803; ThermoScientific) were added into each IP tube and incubated for 1 h at room temperature on a rotator. 15 µg protein was saved as the supernatant fraction for western blot analysis. The IP samples were washed 5 times with IP lysis buffer, then eluted with 4x Laemmli buffer at 500 rpm at room temperature for 20 min. The IP samples were saved for western blot analysis. Western blot analysis of the input, supernatant and IP fractions was performed as previously described[7]. Membranes were blocked in 5% BSA followed by incubation with primary antibodies: CDK6 (rabbit polyclonal, PA5-27978; Invitrogen), Cyclin D3 (mouse, monoclonal; MA5-12717), HSC70 (mouse, monoclonal; sc-7298), STAT5b (rabbit monoclonal; ab178941), pSTAT5 pY694 (mouse; BD611964). Secondary HRP antibodies (Cell Signaling Technology) and VeriBlot for IP Detection Reagent (Abcam,

ab131366) were used, and membranes were visualized using Clarity™ ECL Western blotting substrate (Biorad).

Immunoblotting

Cells were lysed in Laemmli buffer (with PhosSTOP and cOmplete protease inhibitors, Roche), heated at 95°C for 10 min and sonicated in an ultrasonic bath. Proteins were separated on 8-12% SDS-PAGE gels and transferred onto 0.45 μm PVDF membranes (Sigma-Aldrich). Membranes were probed with primary antibodies at a 1:1000 dilution in 3% BSA in py-TBST, unless stated otherwise. The used antibodies were directed against αTubulin (DM1A) (Santa Cruz Biotechnology, Inc., Sc-32293), STAT5b (abcam, 178941, 1:2000), STAT5 (BD Biosciences, BD610192, 1:250), CDK6 (Invitrogen, PA5-27978), ACTB (Santa Cruz Biotechnology, Inc., Sc69879, 1:3000), pRb s780 (Cell Signaling Technology, CS9307S), GAPDH (ABS16, Merck Millipore) or HSC70 (sc-7298, Santa Cruz biotechnology, 1:10000). Secondary HRP antibodies (anti-mouse IgG, HRP-linked Antibody (cs7076, Cell Signaling Technology, 1:7500), anti-rabbit IgG, HRP-linked Antibody (cs7074, Cell Signaling Technology, 1:7500)) were used and membranes were visualized using Clarity™ ECL Western blotting substrate (Biorad).

Viability assay and cell-cycle analysis

To assess the IC_{50}, $3*10^3$ cells were seeded per well (triplicates per concentration and cell line) in round-bottom 96-well plates (Falcon). Cells were treated with serial dilutions of the STAT5 PROTAC AK-2292, the CDK6 PROTAC BSJ-03-123 (THP/Medchemexpress), or Palbociclib (Selleckchem). Etoposide (Selleckchem) treatment was used as a positive control and DMSO (Dimethylsulfoxid) as the vehicle control. After 24 h of incubation, cells were retreated and 72 h after seeding the cell viability was assessed using the CellTiter-Glo 2.0 luminescence assay. Luminescence was measured with a Varioskan™ LUX Multimode Reader and analyzed using the SkanIt™ software. Cells that were treated with a concentration closest to the IC50, were used for a propidium iodide (PI) staining by resuspending cells in PI buffer containing PI (20 μg/mL) and RNase A (100 μg/mL). After a 30 min incubation step at 37°C, cells were analyzed with a Cytoflex S flow cytometer (Beckman Coulter).

Degrader treatments

To evaluate the efficacy of the CDK6 PROTAC BSJ-03-123, NPM-ALK$^{Tg/+}$ cell lines (*n* = 5) were treated for 6 h with 3 µM BSJ-03-123, DMSO (control) or a combination of CDK6 PROTAC BSJ-03-123 and STAT5 PROTAC AK-2292. After treatment, cells were intracellularly stained for CDK6 and protein levels were analyzed by flow cytometry. To assess the functional activity of the STAT5 PROTAC AK-2292, three *NPM-ALK*$^{Tg/+}$ murine cell lines were treated with 1.5 µM AK-2292. Protein lysates were collected after 24, 48, and 72 h, and STAT5 protein levels were analyzed by Western blot.

Generation of human knockout cell lines

CRISPR-mediated genome editing was performed as previously described[8]. In brief, CRISPR guide RNAs targeting the genomic DNA of human *STAT5B* (ENST00000698809.1) were designed using the CHOPCHOPv2 online design tool[9–11] and cloned into LentiCRISPRv2GFP plasmid (Addgene plasmid #82416; https://www.addgene.org/82416/; RRID: Addgene_82416) according to the protocol available from Addgene[12,13]. Two previously published non-targeting guides were used as controls[12]. Lentiviral particles were produced by co-transfecting constructs with packaging and envelope plasmids psPAX2 and pMD2.G in a subclone of the transformed human embryonic kidney cell line (Lenti-X 293T) seeded in 10 cm tissue culture dishes using Lipofectamine 2000 (Life Technologies). psPAX2 and pMD2.G were a gift from Didier Trono (Addgene plasmid #12260; http://n2t.net/addgene:12260 and Addgene plasmid #12259; http://n2t.net/addgene:12259). 72 h post-transfection, viral supernatants were harvested, and virus concentrated using the PEG virus precipitation kit (Abcam), divided into aliquots, snap-frozen in liquid nitrogen and stored at -80°C. $5*10^5$ cells were transduced with 50 µL of viral supernatant in the presence of 10 µg/mL polybrene (Sigma) in 6-well plates for 72 h. Cells were selected after a 14-day quarantine period through FACS-sorting of GFP$^+$ cells.

To target *CDK6,* ALCL cell lines were electroporated with Alt-R CRISPR RNA (IDT) containing a previously described[14,15] CDK6-specific crRNA (targeting the first exon of CDK6: GCCCGCGACTTGAAGAACGG) and fluorescently labeled tracrRNA (ATTO 647). The RNA duplex was assembled by heating equal volumes of 200 µM crRNA and tracrRNA at 95°C for 5 min, followed by incubation at room temperature in the dark. RNA complexes were diluted with PBS before electroporation. $1*10^6$ cells per condition were resuspended in electroporation buffer. Electroporation was performed using the Lonza 4D Nucleofector with the program

DS137. 3 and 5 days after electroporation, single-cell clones were sorted into 96-well plates based on ATTO 647 signal. To determine the effects of CDK6 targeting, PI staining was performed 5 days post - electroporation to evaluate cell-cycle distribution. On day 6, intracellular CDK6 levels were assessed by flow cytometry. Expanded clones were screened for successful CDK6 knockout via immunoblotting.

Name	Sequence (5' – 3')
gRNA-hSTAT5B_1_F	CACCGTAACGCTTGCATCTGATGAA
gRNA-hSTAT5B_1_R	AAACTTCATCAGATGCAAGCGTTAC
gRNA-hSTAT5B_2_F	CACCGCCTCAAACGTCTGGTTGATC
gRNA-hSTAT5B_2_R	AAACGATCAACCAGACGTTTGAGGC
gRNA-hSTAT5B_3_F	CACCGAGCGGGCCAAACTGAGCTAG
gRNA-hSTAT5B_3_R	AAACCTAGCTCAGTTTGGCCCGCTC
NonTargetingCTRLGuideHuman_02_F	caccgCGCTTCCGCGGCCCGTTCAA
NonTargetingCTRLGuideHuman_02_R	aaacTTGAACGGGCCGCGGAAGCGc
NonTargetingCTRLGuideHuman_03_F	caccgATCGTTTCCGCTTAACGGCG
NonTargetingCTRLGuideHuman_03_R	aaacCGCCGTTAAGCGGAAACGATc
gRNA-CDK6	GCCCGCGACTTGAAGAACGG

Murine STAT5B ChIP sequencing data from DN2 and DN3 T cell progenitor populations

Processed ChIP sequencing data in WIG format were downloaded from https://www.ncbi.nlm.nih.gov/geo/query/acc.cgi?acc=GSE283392 on 4.12.2024. Genomic windows with a score of > 50 (DN2 samples) or > 30 (DN3 sample) were taken to further analysis. First, the file format was changed from WIG to BED whereby directly adjacent WIG windows were combined into one peak region. The highest score of the WIG windows contributing to a region became the region's score in the BED file. We then annotated all peaks with the nearest gene in the *Gencode vM25 primary assembly annotation* gene model. The annotation was performed by making use of the GenomicFeatures and GenomicsRanges R packages. We generated a *GRanges* object from the gene model gtf file by running makeTxDbFromGFF() followed by extracting transcripts with the transcripts() function, keeping transcript

and gene IDs. The transcripts were then resized to only contain the transcriptional start site (TSS; i.e. a single base) for each transcript. A second *Granges* object was generated from the peak BED files. The distanceToNearest() function was then used to identify the distance from each peak to the nearest transcriptional start site.

ChIP sequencing in murine and human ALCL cell lines

We performed ChIP targeting CDK6 in a murine cell line and ChIP targeting STAT5B in the following human cell lines: SU-DHL-1, DEL, SUP-M2, Karpas-299, SR-786. $1.5*10^7$ cells were collected, washed twice with ice-cold PBS containing 1 mM PMSF. The cell pellet was then crosslinked with 2 mM DSG in PBS under rotation for 30 min at room temperature, washed twice with PBS containing 1 mM PMSF and incubated with 1% formaldehyde in PBS for 10 min under rotation at room temperature. The formaldehyde crosslinking was quenched by adding a 0.125 M glycine solution and incubating for 5 min under rotation at room temperature. The samples were then washed twice with PBS and subjected to IP. IP was performed as previously described[16] with minor alterations as follows: sheared chromatin was incubated either with 5 µg anti-CDK6 (rabbit polyclonal, PA5-27978; Invitrogen, USA) or anti-IgG (rabbit polyclonal, 02-6102; Invitrogen, USA) and either with 5 µg STAT5b ([EPR16671], Abcam, ab178941) antibody or 5 µg IgG isotype control ([EPR25A], Abcam, ab172730) antibodies at 4°C overnight. Pulled-down DNA was sent for next generation sequencing.

Libraries were prepared at the Vienna Biocenter NGS core facility, using Truseq library prep chemistry. The libraries were sequenced on an Illumina NovaSeqX system (Illumina, San Diego, CA, USA) in single-end mode with 100 bp read length.

ChIP sequencing analysis

Raw data fastq files from different flowcell lanes were concatenated per sample. The concatenated fastq files were then quality controlled using the FASTQC software (version 0.12.1). Trimmomatic (version 0.39) was used to perform adapter trimming and quality-based filtering of reads. After another quality control using FASTQC, the filtered reads of mouse samples were mapped against the GRCm38 primary assembly (Gencode M25), and the filtered reads of human samples against the GRCh38 primary assembly (Gencode

v26) using bwa-mem (version 0.7.15). Alignment QC was done using Qualimap (version 2.2.2a). Blacklisted regions were removed with bedtools subtract. Multimappers and reads with low mapping quality were removed using samtools (version 1.14). We used MACS3 to perform peak calling with default parameters. For each ChIP-seq file we used the corresponding Input file as the control. The resulting "narrowPeak" files were annotated using R and the GenomicFeatures() and GenomidRanges() packages as described above. For human samples, we used the *Gencode v26 primary assembly annotation* gene model. To identify co-bindings of CDK6 and STAT5B we focused on a 500 bp region around the promoter region, defined here as the range around the TSS, ranging from -300 to 200 bp.

Differential gene expression (DGE) across genotypes and clusters in murine scRNA-seq data

To analyze genotype specific transcriptional differences within matched cell states, single- cell data from the *NPM-ALK*$^{Tg/+}$ and *NPM-ALK*$^{Tg/+}$*Cdk6*$^{-/-}$*Stat5b*$^{-/-}$ samples were converted to Seurat objects and normalized. Seurat's FindMarkers() method was used for DGE analysis.

Pathway GSEA using murine scRNA-seq and murine bulk RNA-seq

For Gene Set Enrichment Analysis GSEA, gene names were mapped to Ensembl IDs and rank files were prepared for pre-ranked GSEA. To prepare rank files, differentially expressed genes were filtered to include only those expressed in at least 5% of cells in either group. The ranking metric was calculated as the -log10(p-value) * sign(log fold-change). Using the GSEA application (v4.3.2), we performed the pre-ranked analysis with the hallmark gene set (mh.all.v2024.1.1.Mm.symbols.gmt), a classical enrichment statistic and gene set sizes from 7-500(bulk:hallmark:15-500; other gene sets 6-5000).

Pie Charts for ChIP overlaps in transformation pathways

To identify transcription factor binding in core-enriched genes from the hallmark gene sets, we conducted an overlap analysis, making use of various ChIP-seq datasets. Genes with promoter peaks of STAT5B or CDK6 were identified in previously published STAT5B ChIP-seq data from DN2/3 cells with and without IL7 stimulation (as described above), from a STAT5B ChIP-seq in 5 human cell lines SU-DHL-1, DEL, SUP-M2, Karpas-299, SR-786 and from a CDK6 ChIP-seq from a murine cell line. Promoter peaks were called in a 500 bp genomic interval from -300 to 200 bp around the transcriptional start site. To facilitate visualization,

overlap categories were simplified into four classes: CDK6- only peaks (red), STAT5B- only (blue), Shared peaks (Promoter-binding of CDK6 and STAT5B, yellow) and no peak of CDK6 or STAT5B in the promoter region of core enriched genes (grey).

CDK6/STAT5B expression ordered GSEA analysis

GSEA was performed using the GSEA software (v.4.3.2; Broad Institute), using the gene expression of a single gene as a continuous phenotype label. A normalized gene expression matrix of a human patient ALCL dataset was used (HRA004246 as described above). Patients were filtered to ensure a minimal normalized expression count of 10 in the *ALK* gene (ENSG00000171094). Gene ranking was based on the phenotype label using the expression of a gene of interest (*CDK6* ENSG00000105810; *STAT5B*: ENSG00000173757). Gene ranking was performed using the *Pearson* correlation metric in gene expression-ordered mode. The analysis applied the weighted scoring scheme and the *meandiv* normalization method. Enrichment was evaluated using the MSigDB Hallmark gene sets (v2024.1) and no Hallmark gene set was excluded.

Survival Analysis in human ALCL patients

For the combined cohort analysis, three publicly available gene expression datasets were integrated: GSE58445, GSE19069 (both generated on the Affymetrix Human Genome U133 Plus 2.0 platform, GPL570) and E-TABM-117. The first two datasets were obtained from the Gene Expression Omnibus (GEO), and E-TABM-117 was obtained from ArrayExpress. Preprocessed expression matrices were used as provided and further processed in a consistent manner. Probe sets were mapped to gene symbols using the hgu133plus2.db annotation package. In cases where multiple probe sets mapped to the same gene, the probe with the highest interquartile range (IQR) across samples was selected to retain the most variable and informative signal. The resulting gene-level expression matrices from all three datasets were merged based on common gene symbols. To account for potential batch effects between studies, batch correction was performed using the ComBat algorithm implemented in the sva package. After integration, the combined cohort comprised 74 samples (E-TABM-117: n = 32; GSE58445: n = 24; GSE19069: n = 18), of which 51 were ALK-positive and 23 were ALK-negative. To reduce noise prior to signature scoring, a variance-based filtering step was applied to the integrated expression matrix, retaining genes with a cross-

sample variance ≥ 1 (11,626 genes retained). Of the 60 genes in the CDK6/STAT5B signature identified in the mouse, 52 passed this filter in the human cohort and were used for downstream scoring. For signature scoring and visualization, expression values were z-score normalized on a per-gene basis across the combined cohort. The per-sample signature score was then computed as the mean z-score across the 52 retained signature genes. Clinical annotations, including overall survival time and event status, were extracted from the respective dataset metadata and harmonized across cohorts. Samples lacking survival information had been excluded. A total of 36 survival events were observed in the final cohort of 74 patients. For survival analysis, patients were stratified by signature score using two complementary approaches. In the tercile-split analysis, the cohort was stratified by the top versus bottom tercile of the signature score, with patients in the middle tercile excluded (n = 50 patients were kept). For the dichotomized analysis, the optimal cutpoint was determined using the maximally selected rank statistic with log-rank scoring and the Lau92 p value approximation (maxstat package). Multivariate Cox proportional hazards models including the signature (as a continuous variable or dichotomized as above) and ALK status as covariates were fitted to assess the independent prognostic contribution of the signature.

Human single-cell RNA sequencing

Single cell RNA sequencing data from a CD45+ sorted human ALK+ anaplastic large cell lymphoma primary lymph node biopsy (BioSample SAMN35660560, run accession SRR24860077, Library Name BRI710) were obtained from the NCBI Sequence Read Archive[17].

The filtered count matrix was normalized, and variance stabilized using SCTransform, with the percentage of mitochondrial reads included as a variable to regress out. Principal component analysis was performed on the SCT normalized expression matrix using RunPCA. The first 30 principal components were used as input for both Uniform Manifold Approximation and Projection (RunUMAP, dims = 1:30) for two dimensional visualization, and for the construction of the shared nearest neighbor graph (FindNeighbors, dims = 1:30, k.param = 7). Cell clusters were identified with the Louvain algorithm using FindClusters at a resolution of 0.3.

Identification of cluster marker genes. Cluster specific marker genes were identified using the FindAllMarkers function in Seurat with default parameters, which performs Wilcoxon rank sum tests

between each cluster and all remaining cells. Genes were ranked by average log2 fold change and adjusted *p* value.

Additional PTCL subsets

Gene set scoring and survival analysis. Batch corrected microarray expression data and matched clinical annotation (overall survival time, vital status, age, sex, molecular subtype) for 541 peripheral T cell lymphoma (PTCL) samples were obtained from a publicly available cohort data, which included the following files 541_PTCL_batch_adjusted_geo.id.Rdata and 541_paz_info_MUT.txt; https://github.com/biointf/PTCL)[18]. The analysis was performed within four molecular subtypes with sufficient samples and survival information: PTCL.nos, AITL, NKT, and ATLL. For each subtype, a per sample gene set score was computed by matching probes (Entrez gene IDs annotated to HGNC symbols via biomaRt/Ensembl) to the signature, collapsing multiple probes per gene to their maximum value, and filtering out low variance genes (variance ≤ 0.1 across samples within the subtype). Of the 60 genes identified in the mouse, 49, 46, 52 and 50 genes remained after probe collapse and variance filtering in human AITL, ATLL, PTCL.nos and NKT cohorts respectively and constituted the corresponding gene sets used for the survival analyses. The remaining genes were standardized across samples to mean zero and unit variance, and the per sample score was defined as the mean of these standardized values across all retained signature genes. Within each subtype, the association between score and overall survival was assessed by univariate Cox proportional hazards regression (Surv(time, status) ~ score), yielding a hazard ratio with 95% Wald confidence interval and a two-sided p value. Analyses were performed in R using the survival, biomaRt, and ggplot2 packages.

NPM-ALK program

NPM-ALK transformation signature and CDK6/STAT5B dependent subset. A 95 gene NPM-ALK transformation signature was defined from the mouse scRNA-seq data as the union of (i) genes upregulated in *NPM-ALK*$^{Tg/+}$ versus WT cells in the DN2/3 cluster (cluster 6) and in the transition cluster (cluster 19), (ii) genes upregulated in *NPM-ALK*$^{Tg/+}$ cells along the trajectory from cluster 19 to the tumor cluster (cluster t6), and (iii) genes upregulated along the DN2/3 to transition trajectory specifically in *NPM-ALK*$^{Tg/+}$ cells (i.e. not shared with WT or NPM-ALK$^{Tg/+}$Cdk6$^{-/-}$Stat5b$^{-/-}$ cells). All differential expression tests were performed with

FindMarkers in Seurat using a threshold of adjusted $p < 0.05$ and |log2 fold change| > 0.5. The CDK6/STAT5B dependent subset (35 genes) was defined as the intersection of the 95 gene transformation signature with genes either downregulated in NPM-ALK$^{Tg/+}$Cdk6$^{-/-}$Stat5b$^{-/-}$ versus *NPM-ALK*$^{Tg/+}$ cells in cluster 6 or 19, contained in the previously defined shift gene list.

To project the mouse derived signatures onto the human dataset, mouse gene symbols were mapped to their human orthologues using the Ensembl v108 human and mouse orthologue table (downloaded 2022-11-24). For each cell, a per cell signature score was computed as follows: SCT normalized expression values for the matched signature genes were extracted, restricted to genes with non-zero expression across the dataset, scaled to *z* scores across cells on a per gene basis and averaged across all signature genes within each cell.

Co-expression analysis of CDK6 and STAT5B with DPP4 in 2 human ALCL datasets

To assess the relationship between DPP4 and the combined expression of CDK6 and STAT5B across two independent human ALCL patient cohorts (ALCL patient set1 and ALCL patient set2_ sets, source described in section: Bulk RNA sequencing datasets from ALCL patients), gene expression values were converted to within-sample ranks (min_rank, dplyr; highest expression = rank 1) to mitigate technical variation between datasets. Per-dataset z-scores were then calculated on the ranks for DPP4, CDK6, and STAT5B to remove residual batch effects prior to pooling. A combined CDK6/STAT5B score was computed as the arithmetic mean of the z-scored CDK6 and STAT5B values, z_combined = (z_CDK6 + z_STAT5B) / 2. The association between DPP4 rank and z_combined was quantified using Spearman's rank correlation coefficient (r_s, cor.test, R base, exact = FALSE), calculated separately within each dataset. Linear regression lines with 95% confidence intervals are shown per dataset for visualization only.

Co-expression analysis of *Cdk6* and *Stat5b* in *Dpp4*-stratified cells along pseudotime

To assess whether *Dpp4* expression coincides with elevated *Cdk6* and *Stat5b* during the developmental window preceding tumor cluster emergence, WT and *NPM-ALK*$^{Tg/+}$ cells (excluding *NPM-ALK*$^{Tg/+}$*Cdk6*$^{-/-}$*Stat5b*$^{-/-}$) with pseudotime values between 15 and 23 (core of DN2b-DN3a transition) along the transformation lineage. Log-normalized expression values for *Dpp4, Cdk6*, and *Stat5b* were extracted from

the SingleCellExperiment object. *Cdk6* and *Stat5b* log-counts were z-score transformed across the filtered population, and a combined per-cell score was computed as the mean of the two z-scores. Cells were stratified into *Dpp4*^{low} and *Dpp4*^{high} groups by median split of *Dpp4* log-counts within each genotype. Differences in the combined Cdk6/Stat5b score between *Dpp4*^{low} and *Dpp4*^{high} cells were tested within each genotype by ordinary one-way ANOVA with Sidak's multiple comparisons test.

Immunoprecipitation coupled to mass spectrometry analysis

Samples from SU-DHL-1 and DEL cells used for co-immunoprecipitation were prepared as previously described for the analysis with Western blot, with the following modifications. Briefly, cells were pre-treated with 200 nM Palbociclib overnight and subsequently subjected to starvation in RPMI-1640 medium, without FCS for 20 min and then stimulated for 20 min in RPMI-1640 medium containing 20% FCS. Following protein lysis and determination of protein concentration, 900 µg of SU-DHL-1 and 500 µg of DEL protein lysates were incubated with either 10 µg of CDK6 antibody (rabbit polyclonal, PA5-27978; Invitrogen) or 10 µg of the rabbit IgG isotype control antibody (IgG-R; 02-6102, Invitrogen). Immunoprecipitated complexes were then captured on 35 µL of Pierce™ A/G Magnetic Beads (Ref 88803; ThermoScientific).

The beads were transferred into 200 µL tubes using 70, 65 and 65 µL 100 mM TEAB. After washing of the beads three times with 200 µL 100 mM TEAB, beads were dissolved in 50 µL 100 mM TEAB. For reduction, 2.6 µL of 100 mM TCEP in 100 mM TEAB (30 min 37°C). Then, for alkylation, 2.1 µL of 400 mM IAA in 100 mM TEAB were added (30 min 25°C). Finally, remaining IAA was quenched with 2.9 µL 100 mM TCEP in 100 mM TEAB (10 min 37°C).

For tryptic digest 2 µL of Trypsin/LysC Mix (Promega, Madison, WI) were added. Digest proceeded for 14 h at 37°C, followed by cool-down to 4°C. Digested peptides were removed using the magnetic rack and transferred to a fresh tube. Beads were washed twice with 50 µL 100 mM TEAB. The three portions of extracted peptides were combined, and digest was stopped by addition of 2 µL conc. TFA. Before LC-MS analysis, peptide extracts were desalted and cleaned up using C18 spin tips according to the manufacturer's protocol. The digested protein sample was dissolved in 40 µL 0.1% TFA. 600 ng digested

protein were loaded onto Evotips (EV2011 Evotip Pure, Evosep Biosystems, Odense, Denmark) using the manufacturer's standard protocol[19].

For mass spectrometric data acquisition, an Evosep One HPLC (Evosep Biosystems) was coupled with a timsTOF HT mass spectrometer (Bruker, Massachusetts, USA) via a Captive Spray II ion source (Bruker). Peptides were separated using the standardized 30 samples per day LC gradient method[20] with an Evosep Performance Column (15 cm x 150 µm x 1.5 µm) (EV1137, Evosep).

Data acquisition was performed in dia-PASEF mode with a MS1 scan range of 100–1,700 m/z. Six static m/z and ion mobility windows (50 Da width) were selected for serial MS2 fragmentation ranging from 368 to 1153 m/z and 0.65 to 1.45 V·s/cm² 1/K0 for mass and ion mobility range, respectively. Ramp and accumulation times were set to 100 ms, resulting in a cycle time of 0.74 s.

All dia-PASEF data were analyzed using Spectronaut version 20.4 (Biognosys AG, Schlieren, Switzerland). The directDIA+ (Deep) pipeline was used with default settings with minor modifications. In brief, Carbamidomethylation (C) was used as fixed modification and Deamidation (NQ) and Oxidation (M) were used as variable modifications. The PSM, Peptide, and Protein group false discovery rates (FDR) were set to 1% during the Pulsar search. Proteotypicity filter was set to "only protein group specific". Precursor and Protein Qvalue cutoff as well as Protein PEP cutoff were set to 0.01. No imputation was applied during quantification of the analytes.

Database searches were performed in a database downloaded from Uniprot for *Homo sapiens* (taxonomy ID 9606, downloaded 2025-06-12, 83,374 entries), together with the cRAP database with common contaminants (downloaded from www.thegpm.org and human entries were removed manually).

Raw protein abundances were normalized for total peptide abundance without imputation and exported for further statistical analysis.

Chromatin state at CDK6 and STAT5B target loci

Gene-level analyses used the mm10 assembly, with gene models and symbols from TxDb.Mmusculus.UCSC.mm10.knownGene and org.Mm.eg.db. Promoters were defined as the window spanning 1000 bp upstream to 1000 bp downstream of each annotated transcription start site (TSS). CDK6 and STAT5B ChIP-seq peaks were assigned to genes by promoter overlap, and each gene was classified by its binding status into one of four groups: co-bound by CDK6 and STAT5B (Both), CDK6 bound only, STAT5B bound only, or unbound by either factor (No Peak). Two gene sets were analyzed in parallel, the shift genes dynamically regulated during the DN2 to DN3 transition and the chromatin organization genes. Histone modification and CTCF occupancy were obtained from the ImmGen chromatin compendium for DN2a and DN3 pro-T cell progenitors, covering H3K4me3, H3K4me1, H3K27ac, H3K36me3, H3K27me3 and CTCF. The UCSC bigWigToBedGraph tool was used to load bigwig data for each chromatin mark and cell type from https://genomebrowser-uploads.hms.harvard.edu/data/ly82/ (accessed on 10 March 2026) and convert them to bedgraph files. Peak calling was performed in two steps using MACS3 bdgpeakcall. First, the setting *--cutoff-analysis* was used to determine individual cutoff values per sample. Next, peak calling was performed using these cutoff values. A gene was scored as carrying a given mark when any ImmGen peak for that mark and cell type overlapped its promoter window. For each mark, cell type and gene set, the percentage of genes carrying the mark was computed within each binding group, and enrichment relative to the unbound (No Peak) group was assessed by two-sided Fisher's exact test per comparison.

Human ALCL extension

Human analyses used the hg38 assembly (TxDb.Hsapiens.UCSC.hg38.knownGene, org.Hs.eg.db) with promoters defined identically as TSS ±1000 bp. STAT5B ChIP-seq peaks for ALK-positive (SU-DHL-1, DEL, SUP-M2, Karpas-299, SR-786) and ALK-negative (Mac-1, Mac-2a) ALCL cell lines were assigned to promoters, retaining only peaks falling within 1000 bp of a TSS to match the promoter window. Genes were grouped by STAT5B occupancy into three categories: STAT5B peaks present in ALK-positive samples, STAT5B peaks present in ALK-negative samples, or no STAT5B peak. H3K27ac ChIP-seq data for two primary ALCL biopsies (cases 54 and 208), two ALK-positive cell lines (SU-DHL-1, SUP-M2) and two ALK-negative cell lines (Mac-1, Mac-2A) were obtained by generating a download script at

https://www.ebi.ac.uk/ena/browser/view/PRJNA667133 (accessed 13 March 2026). The data were analysed using our in-house ChIP seq pipeline as described above and narrowPeak files were generated. Peaks within each sample group were merged into a single set of non-overlapping intervals before promoter assignment. The percentage of genes carrying H3K27ac at the promoter was computed for each STAT5B group within each H3K27ac sample context (primary ALCL, ALK-positive cell lines, ALK-negative cell lines), and enrichment was tested against the no STAT5B peak group by two-sided Fisher's exact test.

To relate the murine and human regulatory landscapes, the murine shift gene and chromatin organization gene groups defined above (Both, CDK6 only, STAT5B only, No Peak) were converted to human orthologs. Mouse symbols were mapped to Entrez identifiers (org.Mm.eg.db) and to human orthologs with homologene (inTax 10090, outTax 9606), and the resulting human genes were intersected with the hg38 promoter universe. For each ortholog group, the percentage of genes carrying concurrent STAT5B occupancy and H3K27ac at their promoter was computed in primary ALCL, ALK-positive cell lines and ALK-negative cell lines and compared against the unbound (No Peak) group by two-sided Fisher's exact test (significance thresholds as above). All peak to promoter overlaps were computed with GenomicRanges and IRanges.

REFERENCES

1. Kollmann S, Grausenburger R, Klampfl T, et al. A STAT5B-CD9 axis determines self-renewal in hematopoietic and leukemic stem cells. *Blood*. 2021;138(23):2347-2359.
2. McInnes L, Healy J, Melville J. *UMAP: Uniform Manifold Approximation and Projection for Dimension Reduction*; 2018. http://arxiv.org/pdf/1802.03426.
3. van Gassen S, Callebaut B, van Helden MJ, et al. FlowSOM: Using self-organizing maps for visualization and interpretation of cytometry data. *Cytometry. Part A : the journal of the International Society for Analytical Cytology*. 2015;87(7):636-645. Published January 8, 2015.
4. Galvin A, Weglarz M, Folz-Donahue K, et al. Cell Cycle Analysis of Hematopoietic Stem and Progenitor Cells by Multicolor Flow Cytometry. *Current protocols in cytometry*. 2019;87(1):e50. Published October 18, 2018.
5. Gärtner F, Alt FW, Monroe R, et al. Immature thymocytes employ distinct signaling pathways for allelic exclusion versus differentiation and expansion. *Immunity*. 1999;10(5):537-546.
6. Shin SB, Lo BC, Ghaedi M, et al. Abortive γδTCR rearrangements suggest ILC2s are derived from T-cell precursors. *Blood Advances*. 2020;4(21):5362-5372.

7. Schirripa A, Schöppe H, Nebenfuehr S, et al. Cdk6's functions are critically regulated by its unique C-terminus. *iScience*. 2025;28(2):111697. Published December 27, 2024.
8. Garces de Los Fayos Alonso I, Zujo L, Wiest I, et al. PDGFRβ promotes oncogenic progression via STAT3/STAT5 hyperactivation in anaplastic large cell lymphoma. *Molecular cancer*. 2022;21(1):172. Published August 31, 2022.
9. Labun K, Montague TG, Gagnon JA, Thyme SB, Valen E. CHOPCHOP v2: a web tool for the next generation of CRISPR genome engineering. *Nucleic acids research*. 2016;44(W1):W272-6. Published May 16, 2016.
10. Labun K, Montague TG, Krause M, Torres Cleuren YN, Tjeldnes H, Valen E. CHOPCHOP v3: expanding the CRISPR web toolbox beyond genome editing. *Nucleic acids research*. 2019;47(W1):W171-W174.
11. Montague TG, Cruz JM, Gagnon JA, Church GM, Valen E. CHOPCHOP: a CRISPR/Cas9 and TALEN web tool for genome editing. *Nucleic acids research*. 2014;42(Web Server issue):W401-7. Published May 26, 2014.
12. Sanjana NE, Shalem O, Zhang F. Improved vectors and genome-wide libraries for CRISPR screening. *Nature methods*. 2014;11(8):783-784.
13. Shalem O, Sanjana NE, Hartenian E, et al. Genome-scale CRISPR-Cas9 knockout screening in human cells. *Science (New York, N.Y.)*. 2014;343(6166):84-87. Published December 12, 2013.
14. Mayer IM, Doma E, Klampfl T, et al. Kinase-inactivated CDK6 preserves the long-term functionality of adult hematopoietic stem cells. *Blood*. 2024;144(2):156-170.
15. Heller G, Nebenfuehr S, Bellutti F, et al. The Effect of CDK6 Expression on DNA Methylation and DNMT3B Regulation. *iScience*. 2020;23(10):101602. Published October 7, 2020.
16. Platanitis E, Demiroz D, Schneller A, et al. A molecular switch from STAT2-IRF9 to ISGF3 underlies interferon-induced gene transcription. *Nature communications*. 2019;10(1):2921. Published July 2, 2019.
17. Mastini C, Campisi M, Patrucco E, et al. Targeting CCR7-PI3Kγ overcomes resistance to tyrosine kinase inhibitors in ALK-rearranged lymphoma. *Science translational medicine*. 2023;15(702):eabo3826. Published June 28, 2023.
18. Maura F, Agnelli L, Leongamornlert D, et al. Integration of transcriptional and mutational data simplifies the stratification of peripheral T-cell lymphoma. *American journal of hematology*. 2019;94(6):628-634. Published March 19, 2019.
19. Evosep Biosystems. Loading protocol for Evotip Pure; www.evosep.com/wp-content/uploads/2024/03/PR-001D-Sample-loading-protocol_WEB.pdf.
20. Evosep Biosystems. A standardized separation method with a throughput of 30 samples per day; https://www.evosep.com/wp-content/uploads/2024/03/AN-009C-30SPD-web.pdf.

Fig. S1

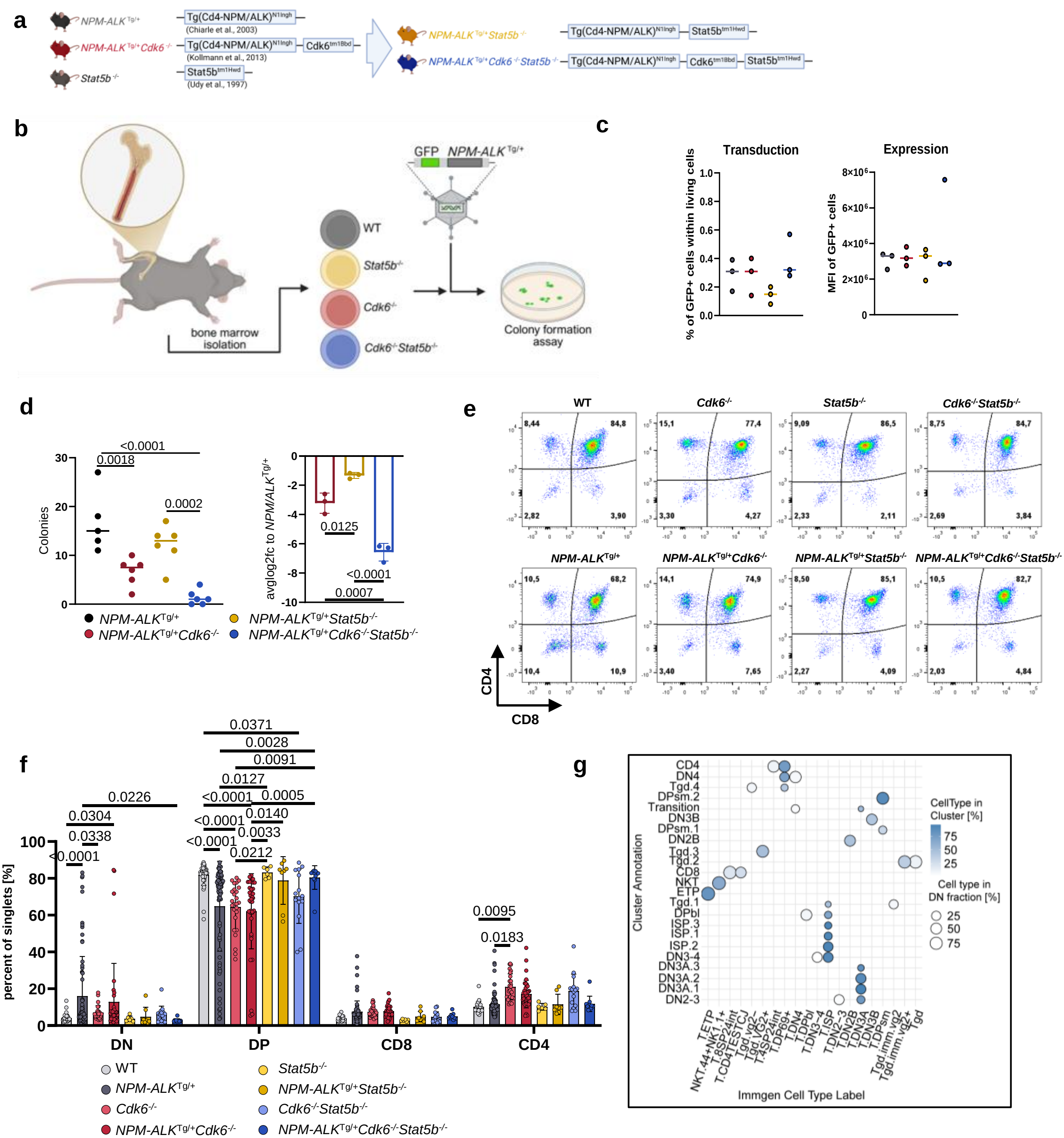
a
NPM-ALK Tg/+
NPM-ALK Tg/+ Cdk6-/-
Stat5b-/-
Tg(Cd4-NPM/ALK)N1Ingh
(Chiarle et al., 2003)
Cdk6tm1Bbd
(Kollmann et al., 2013)
Stat5btm1Hwd
(Udy et al., 1997)
NPM-ALK Tg/+ Stat5b-/-
NPM-ALK Tg/+ Cdk6-/- Stat5b-/-
b
GFP NPM-ALK Tg/+
WT
Stat5b-/-
Cdk6-/-
Cdk6-/-Stat5b-/-
bone marrow isolation
Colony formation assay
c
Transduction
Expression
% of GFP+ cells within living cells
MFI of GFP+ cells
d
Colonies
avglog2fc to NPM/ALK Tg/+
<0.0001
0.0018
0.0002
0.0125
<0.0001
0.0007
NPM-ALKTg/+
NPM-ALKTg/+Cdk6-/-
NPM-ALKTg/+Stat5b-/-
NPM-ALKTg/+Cdk6-/-Stat5b-/-
e
WT
Cdk6-/-
Stat5b-/-
Cdk6-/-Stat5b-/-
NPM-ALKTg/+
NPM-ALKTg/+Cdk6-/-
NPM-ALKTg/+Stat5b-/-
NPM-ALKTg/+Cdk6-/-Stat5b-/-
CD4
CD8
f
percent of singlets [%]
DN
DP
CD8
CD4
WT
NPM-ALKTg/+
Cdk6-/-
NPM-ALKTg/+Cdk6-/-
Stat5b-/-
NPM-ALKTg/+Stat5b-/-
Cdk6-/-Stat5b-/-
NPM-ALKTg/+Cdk6-/-Stat5b-/-
g
Cluster Annotation
Immgen Cell Type Label
CellType in Cluster [%]
Cell type in DN fraction [%]

Fig. S1

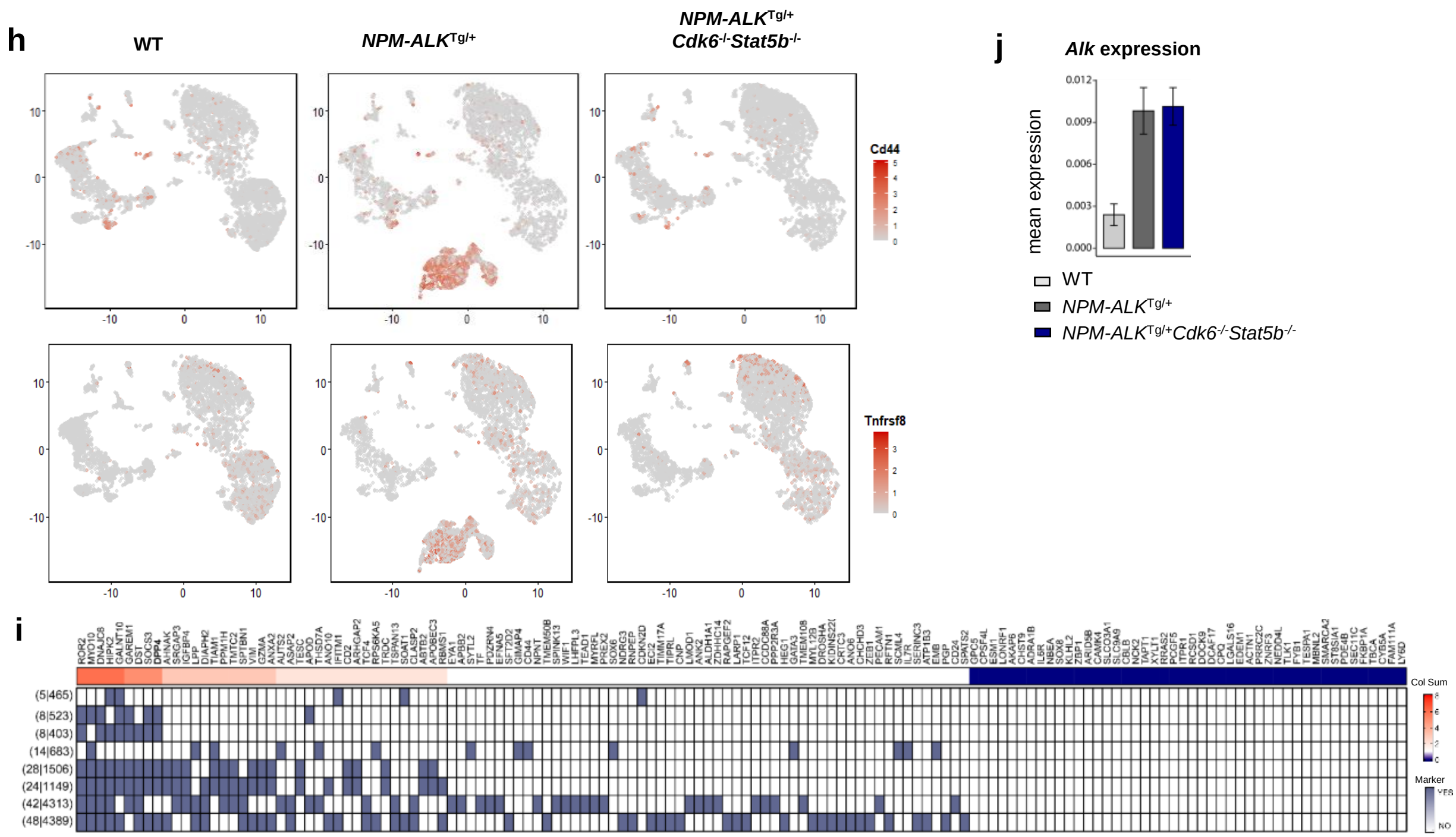

h
WT
NPM-ALK^Tg/+
NPM-ALK^Tg/+ Cdk6^-/-Stat5b^-/-
Cd44
Tnfrsf8
i
(5|465)
(8|523)
(8|403)
(14|683)
(28|1506)
(24|1149)
(42|4313)
(48|4389)
Col Sum
Marker
j
Alk expression
mean expression
0.012
0.009
0.006
0.003
0.000
WT
NPM-ALK^Tg/+
NPM-ALK^Tg/+Cdk6^-/-Stat5b^-/-

Fig. S2

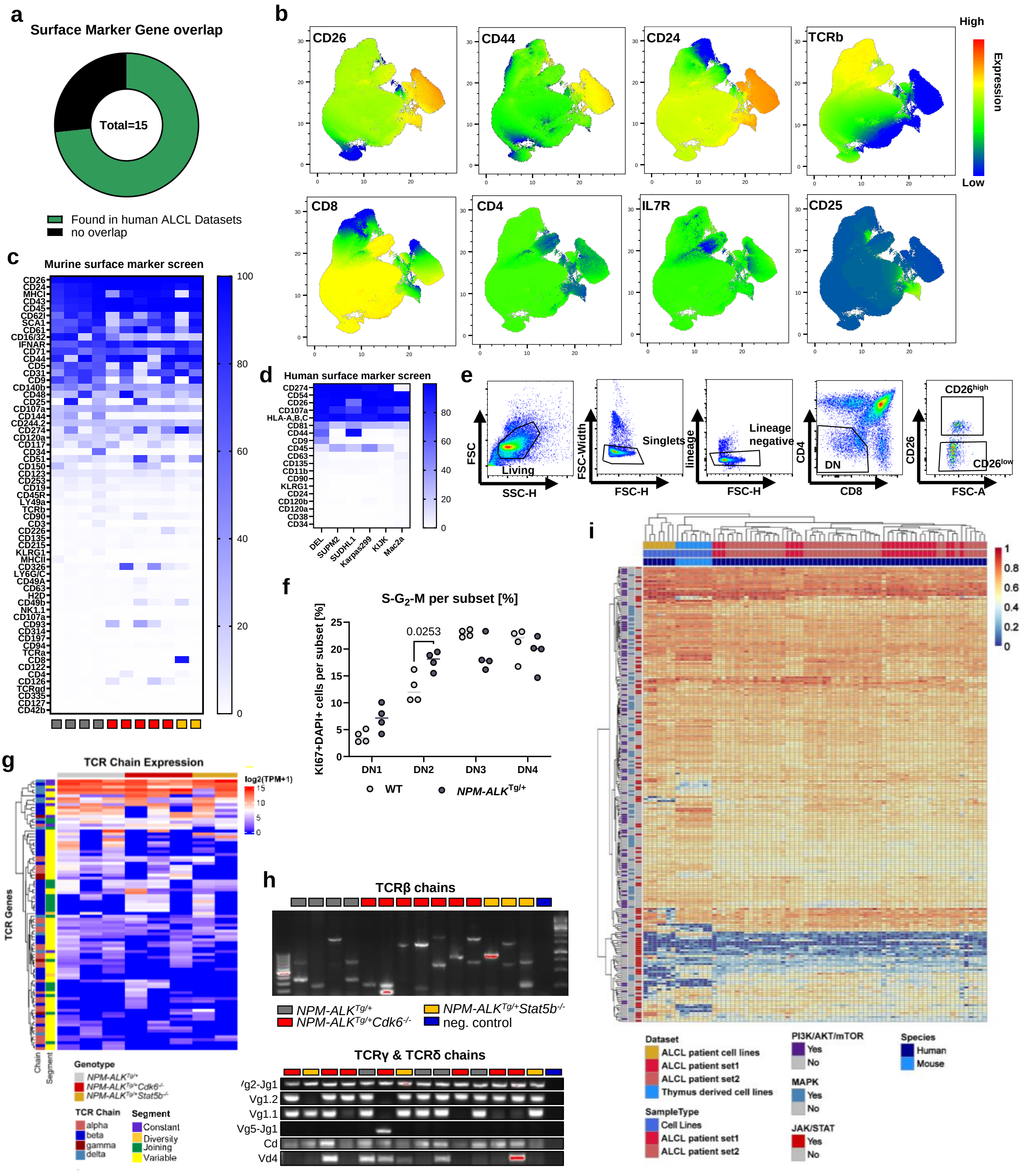

a
Surface Marker Gene overlap
Total=15
Found in human ALCL Datasets
no overlap
b
CD26
CD44
CD24
TCRb
CD8
CD4
IL7R
CD25
High
Expression
Low
c
Murine surface marker screen
CD26 CD24 MHCI CD43 CD45 CD62l SCA1 CD61 CD16/32 IFNAR CD71 CD44 CD5 CD31 CD9 CD140b CD48 CD25 CD107a CD144 CD244.2 CD274 CD120a CD117 CD34 CD51 CD150 CD123 CD253 CD19 CD45R LY49a TCRb CD90 CD3 CD226 CD135 CD215 KLRG1 MHCII CD326 LY6G/C CD49A CD63 H2D CD49b NK1.1 CD107a CD93 CD314 CD197 CD94 TCRa CD8 CD122 CD4 CD126 TCRgd CD335 CD127 CD42b
100
80
60
40
20
0
d
Human surface marker screen
CD274 CD54 CD26 CD107a HLA-A,B,C CD81 CD44 CD9 CD45 CD63 CD135 CD11b CD90 KLRG1 CD24 CD120b CD120a CD38 CD34
DEL SUPM2 SUDHL1 Karpas299 KIJK Mac2a
e
FSC
SSC-H
Living
FSC-Width
FSC-H
Singlets
lineage
FSC-H
Lineage negative
CD4
CD8
DN
CD26
FSC-A
CD26high
CD26low
f
S-G2-M per subset [%]
Ki67+DAPI+ cells per subset [%]
0.0253
DN1
DN2
DN3
DN4
WT
NPM-ALKTg/+
g
TCR Chain Expression
log2(TPM+1)
TCR Genes
Chain
Segment
Genotype
NPM-ALKTg/+
NPM-ALKTg/+Cdk6-/-
NPM-ALKTg/+Stat5b-/-
TCR Chain
alpha
beta
gamma
delta
Segment
Constant
Diversity
Joining
Variable
h
TCRβ chains
NPM-ALKTg/+
NPM-ALKTg/+Cdk6-/-
NPM-ALKTg/+Stat5b-/-
neg. control
TCRγ & TCRδ chains
Vg2-Jg1
Vg1.2
Vg1.1
Vg5-Jg1
Cd
Vd4
i
Dataset
ALCL patient cell lines
ALCL patient set1
ALCL patient set2
Thymus derived cell lines
SampleType
Cell Lines
ALCL patient set1
ALCL patient set2
PI3K/AKT/mTOR
Yes
No
MAPK
Yes
No
JAK/STAT
Yes
No
Species
Human
Mouse

Fig. S2

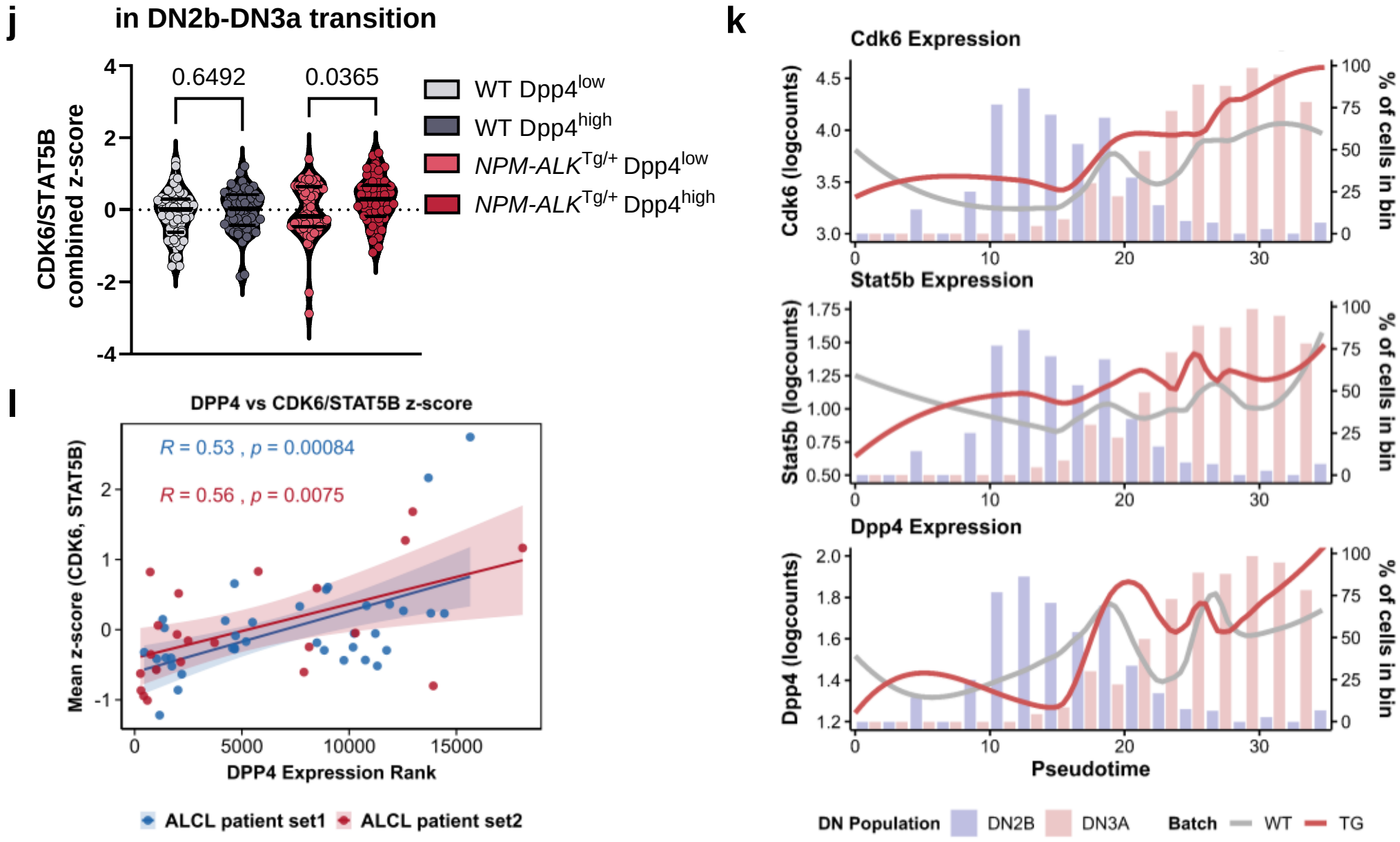

j
in DN2b-DN3a transition
CDK6/STAT5B combined z-score
0.6492
0.0365
WT Dpp4low
WT Dpp4high
NPM-ALKTg/+ Dpp4low
NPM-ALKTg/+ Dpp4high
k
Cdk6 Expression
Cdk6 (logcounts)
% of cells in bin
Stat5b Expression
Stat5b (logcounts)
Dpp4 Expression
Dpp4 (logcounts)
Pseudotime
DN Population
DN2B
DN3A
Batch
WT
TG
l
DPP4 vs CDK6/STAT5B z-score
R = 0.53 , p = 0.00084
R = 0.56 , p = 0.0075
Mean z-score (CDK6, STAT5B)
DPP4 Expression Rank
ALCL patient set1
ALCL patient set2

Fig. S3

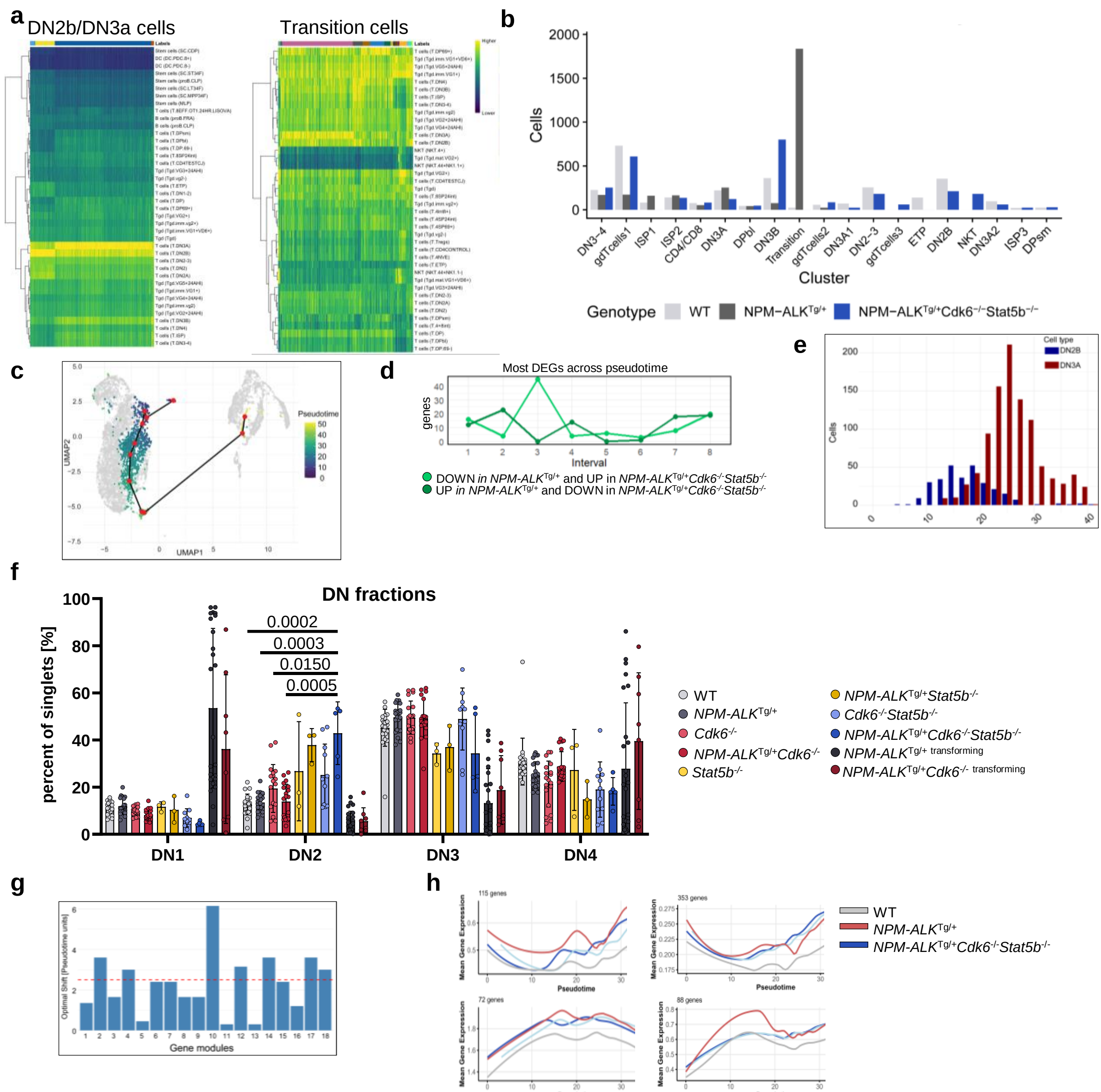

a
DN2b/DN3a cells
Transition cells
b
Cells
2000
1500
1000
500
0
DN3-4
gdTcells1
ISP1
ISP2
CD4/CD8
DN3A
DPbl
DN3B
Transition
gdTcells2
DN3A1
DN2-3
gdTcells3
ETP
DN2B
NKT
DN3A2
ISP3
DPsm
Cluster
Genotype WT NPM−ALK$^{Tg/+}$ NPM−ALK$^{Tg/+}$Cdk6$^{-/-}$Stat5b$^{-/-}$
c
UMAP1
UMAP2
Pseudotime
d
Most DEGs across pseudotime
genes
Interval
DOWN *in NPM-ALK$^{Tg/+}$* and UP in *NPM-ALK$^{Tg/+}$Cdk6$^{-/-}$Stat5b$^{-/-}$*
UP *in NPM-ALK$^{Tg/+}$* and DOWN in *NPM-ALK$^{Tg/+}$Cdk6$^{-/-}$Stat5b$^{-/-}$*
e
Cell type
DN2B
DN3A
Cells
f
DN fractions
percent of singlets [%]
0.0002
0.0003
0.0150
0.0005
DN1
DN2
DN3
DN4
WT
NPM-ALK$^{Tg/+}$
Cdk6$^{-/-}$
NPM-ALK$^{Tg/+}$Cdk6$^{-/-}$
Stat5b$^{-/-}$
NPM-ALK$^{Tg/+}$Stat5b$^{-/-}$
Cdk6$^{-/-}$Stat5b$^{-/-}$
NPM-ALK$^{Tg/+}$Cdk6$^{-/-}$Stat5b$^{-/-}$
NPM-ALK$^{Tg/+}$ transforming
NPM-ALK$^{Tg/+}$Cdk6$^{-/-}$ transforming
g
Optimal Shift (Pseudotime units)
Gene modules
h
115 genes
353 genes
72 genes
88 genes
Mean Gene Expression
Pseudotime
WT
NPM-ALK$^{Tg/+}$
NPM-ALK$^{Tg/+}$Cdk6$^{-/-}$Stat5b$^{-/-}$

Fig. S4

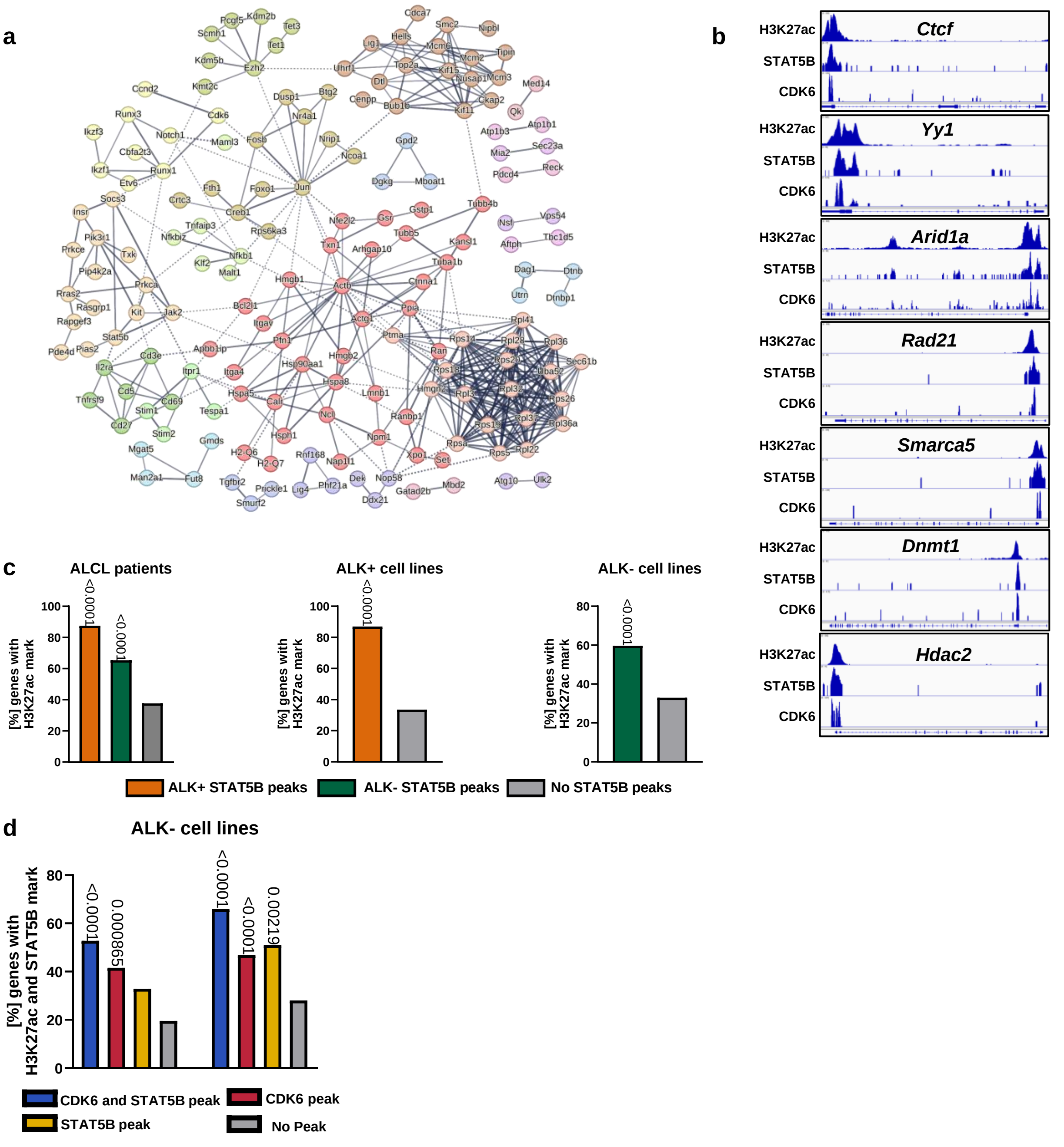
a
b
Ctcf
Yy1
Arid1a
Rad21
Smarca5
Dnmt1
Hdac2
H3K27ac
STAT5B
CDK6
c
ALCL patients
ALK+ cell lines
ALK- cell lines
[%] genes with H3K27ac mark
<0.0001
ALK+ STAT5B peaks
ALK- STAT5B peaks
No STAT5B peaks
d
ALK- cell lines
[%] genes with H3K27ac and STAT5B mark
<0.0001
0.000865
<0.0001
0.00219
CDK6 and STAT5B peak
CDK6 peak
STAT5B peak
No Peak

Fig. S5

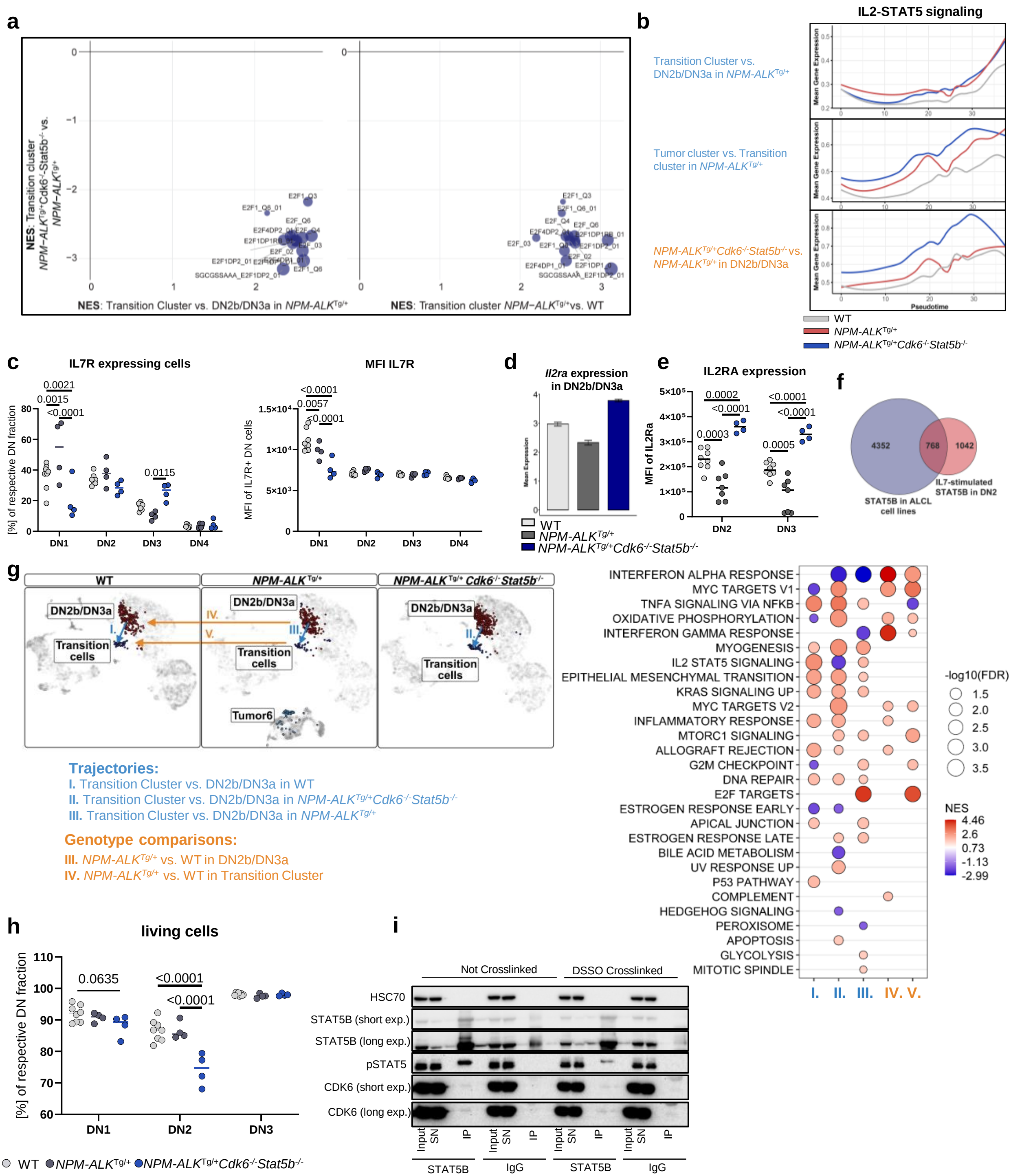

a
NES: Transition cluster NPM-ALKTg/+Cdk6-/-Stat5b-/- vs. NPM-ALKTg/+
NES: Transition Cluster vs. DN2b/DN3a in NPM-ALKTg/+
NES: Transition cluster NPM-ALKTg/+ vs. WT
b
IL2-STAT5 signaling
Transition Cluster vs. DN2b/DN3a in NPM-ALKTg/+
Tumor cluster vs. Transition cluster in NPM-ALKTg/+
NPM-ALKTg/+Cdk6-/-Stat5b-/- vs. NPM-ALKTg/+ in DN2b/DN3a
Mean Gene Expression
Pseudotime
WT
NPM-ALKTg/+
NPM-ALKTg/+Cdk6-/-Stat5b-/-
c
IL7R expressing cells
[%] of respective DN fraction
MFI IL7R
MFI of IL7R+ DN cells
DN1
DN2
DN3
DN4
d
Il2ra expression in DN2b/DN3a
Mean Expression
e
IL2RA expression
MFI of IL2Ra
f
4352
768
1042
STAT5B in ALCL cell lines
IL7-stimulated STAT5B in DN2
g
WT
NPM-ALKTg/+
NPM-ALKTg/+ Cdk6-/- Stat5b-/-
DN2b/DN3a
Transition cells
Tumor6
Trajectories:
I. Transition Cluster vs. DN2b/DN3a in WT
II. Transition Cluster vs. DN2b/DN3a in NPM-ALKTg/+Cdk6-/-Stat5b-/-
III. Transition Cluster vs. DN2b/DN3a in NPM-ALKTg/+
Genotype comparisons:
III. NPM-ALKTg/+ vs. WT in DN2b/DN3a
IV. NPM-ALKTg/+ vs. WT in Transition Cluster
INTERFERON ALPHA RESPONSE
MYC TARGETS V1
TNFA SIGNALING VIA NFKB
OXIDATIVE PHOSPHORYLATION
INTERFERON GAMMA RESPONSE
MYOGENESIS
IL2 STAT5 SIGNALING
EPITHELIAL MESENCHYMAL TRANSITION
KRAS SIGNALING UP
MYC TARGETS V2
INFLAMMATORY RESPONSE
MTORC1 SIGNALING
ALLOGRAFT REJECTION
G2M CHECKPOINT
DNA REPAIR
E2F TARGETS
ESTROGEN RESPONSE EARLY
APICAL JUNCTION
ESTROGEN RESPONSE LATE
BILE ACID METABOLISM
UV RESPONSE UP
P53 PATHWAY
COMPLEMENT
HEDGEHOG SIGNALING
PEROXISOME
APOPTOSIS
GLYCOLYSIS
MITOTIC SPINDLE
I. II. III. IV. V.
-log10(FDR)
NES
h
living cells
[%] of respective DN fraction
i
Not Crosslinked
DSSO Crosslinked
HSC70
STAT5B (short exp.)
STAT5B (long exp.)
pSTAT5
CDK6 (short exp.)
CDK6 (long exp.)
Input
SN
IP
STAT5B
IgG
WT NPM-ALKTg/+ NPM-ALKTg/+Cdk6-/-Stat5b-/-

Fig. S5

j

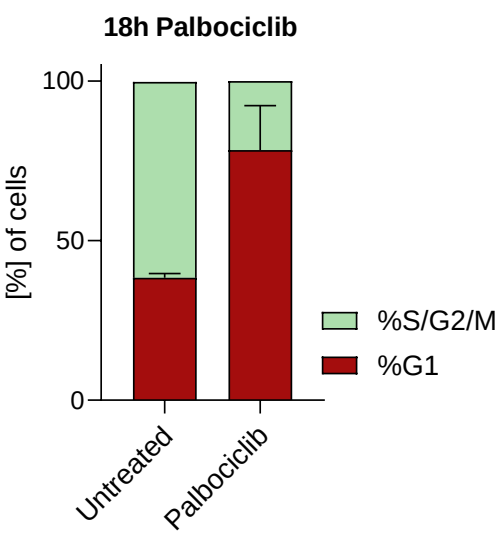

18h Palbociclib
[%] of cells
100
50
0
Untreated
Palbociclib
%S/G2/M
%G1

k

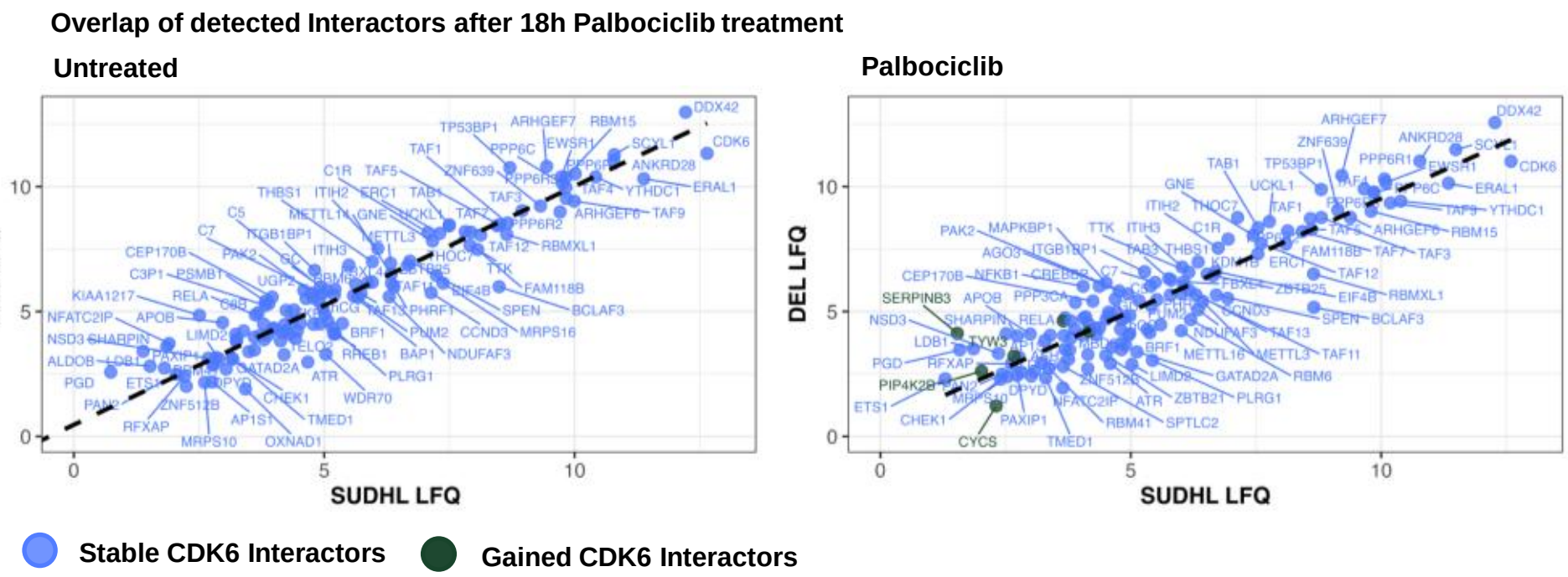

Overlap of detected Interactors after 18h Palbociclib treatment
Untreated
Palbociclib
DEL LFQ
SUDHL LFQ
Stable CDK6 Interactors
Gained CDK6 Interactors

l

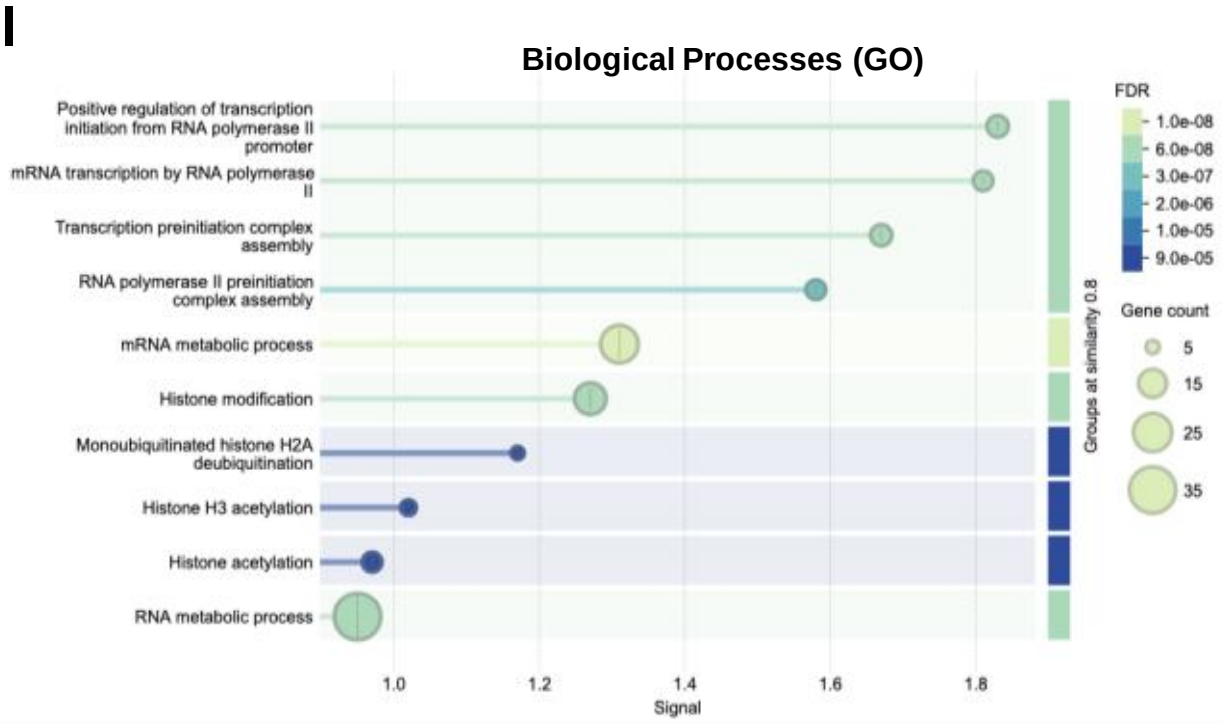

Biological Processes (GO)
Positive regulation of transcription initiation from RNA polymerase II promoter
mRNA transcription by RNA polymerase II
Transcription preinitiation complex assembly
RNA polymerase II preinitiation complex assembly
mRNA metabolic process
Histone modification
Monoubiquitinated histone H2A deubiquitination
Histone H3 acetylation
Histone acetylation
RNA metabolic process
Signal
Groups at similarity 0.8
FDR
1.0e-08
6.0e-08
3.0e-07
2.0e-06
1.0e-05
9.0e-05
Gene count
5
15
25
35

m

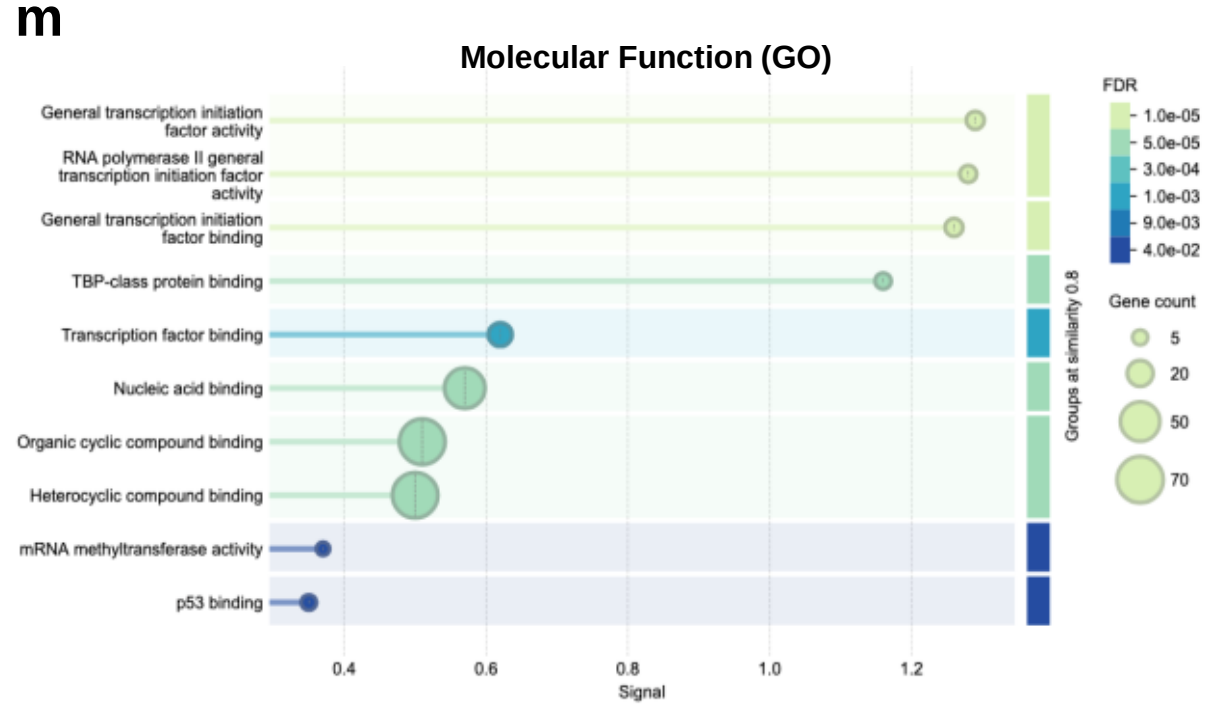

Molecular Function (GO)
General transcription initiation factor activity
RNA polymerase II general transcription initiation factor activity
General transcription initiation factor binding
TBP-class protein binding
Transcription factor binding
Nucleic acid binding
Organic cyclic compound binding
Heterocyclic compound binding
mRNA methyltransferase activity
p53 binding
Signal
Groups at similarity 0.8
FDR
1.0e-05
5.0e-05
3.0e-04
1.0e-03
9.0e-03
4.0e-02
Gene count
5
20
50
70

Fig. S6

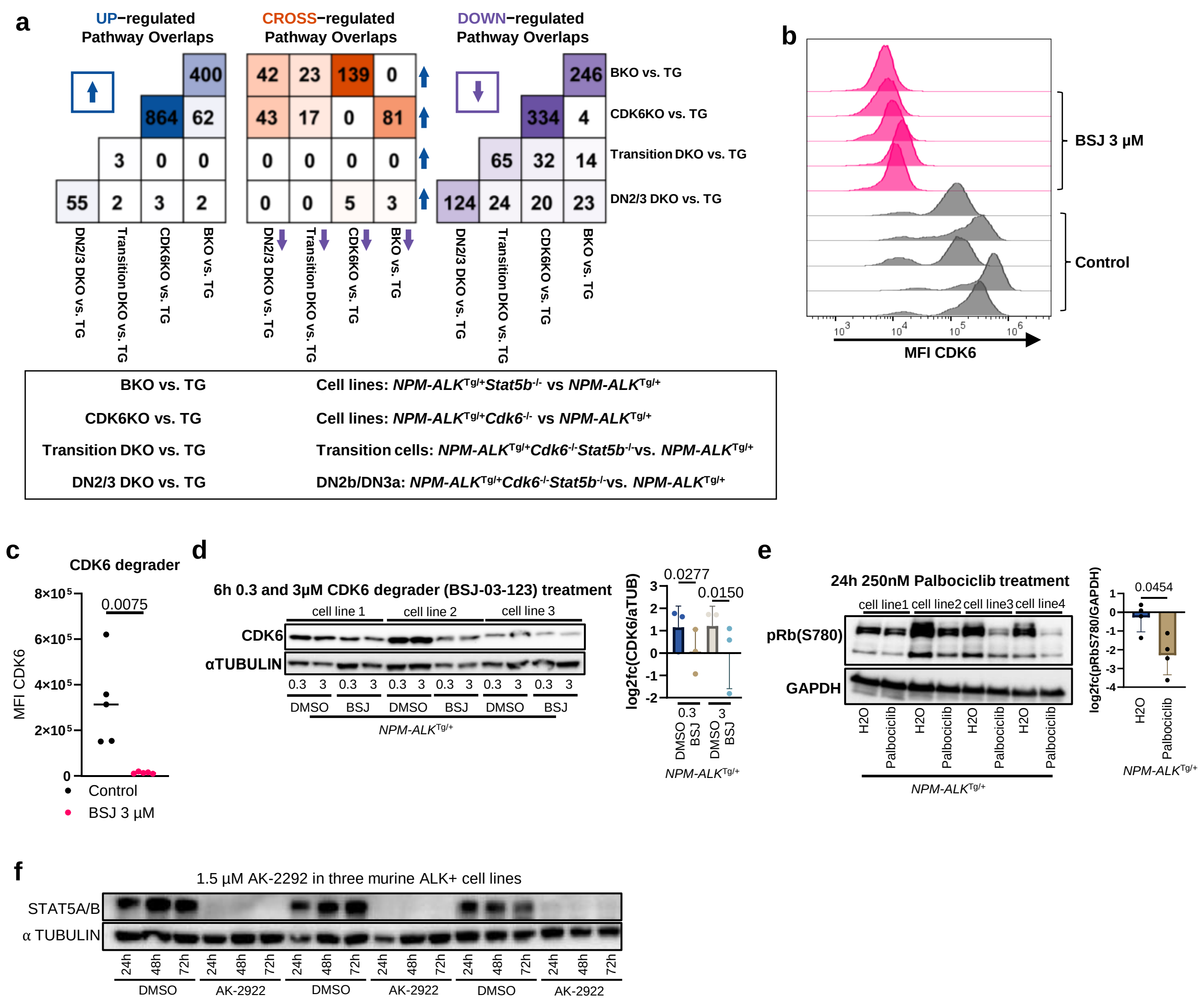
a
UP−regulated Pathway Overlaps
CROSS−regulated Pathway Overlaps
DOWN−regulated Pathway Overlaps
BKO vs. TG
CDK6KO vs. TG
Transition DKO vs. TG
DN2/3 DKO vs. TG
b
BSJ 3 µM
Control
MFI CDK6
BKO vs. TG — Cell lines: NPM-ALK Tg/+ Stat5b-/- vs NPM-ALK Tg/+
CDK6KO vs. TG — Cell lines: NPM-ALK Tg/+ Cdk6-/- vs NPM-ALK Tg/+
Transition DKO vs. TG — Transition cells: NPM-ALK Tg/+ Cdk6-/- Stat5b-/- vs. NPM-ALK Tg/+
DN2/3 DKO vs. TG — DN2b/DN3a: NPM-ALK Tg/+ Cdk6-/- Stat5b-/- vs. NPM-ALK Tg/+
c
CDK6 degrader
MFI CDK6
0.0075
Control
BSJ 3 µM
d
6h 0.3 and 3µM CDK6 degrader (BSJ-03-123) treatment
cell line 1
cell line 2
cell line 3
CDK6
αTUBULIN
DMSO
BSJ
NPM-ALK Tg/+
log2fc(CDK6/aTUB)
0.0277
0.0150
e
24h 250nM Palbociclib treatment
cell line1
cell line2
cell line3
cell line4
pRb(S780)
GAPDH
H2O
Palbociclib
NPM-ALK Tg/+
log2fc(pRbS780/GAPDH)
0.0454
f
1.5 µM AK-2292 in three murine ALK+ cell lines
STAT5A/B
α TUBULIN
24h
48h
72h
DMSO
AK-2922

Fig. S7

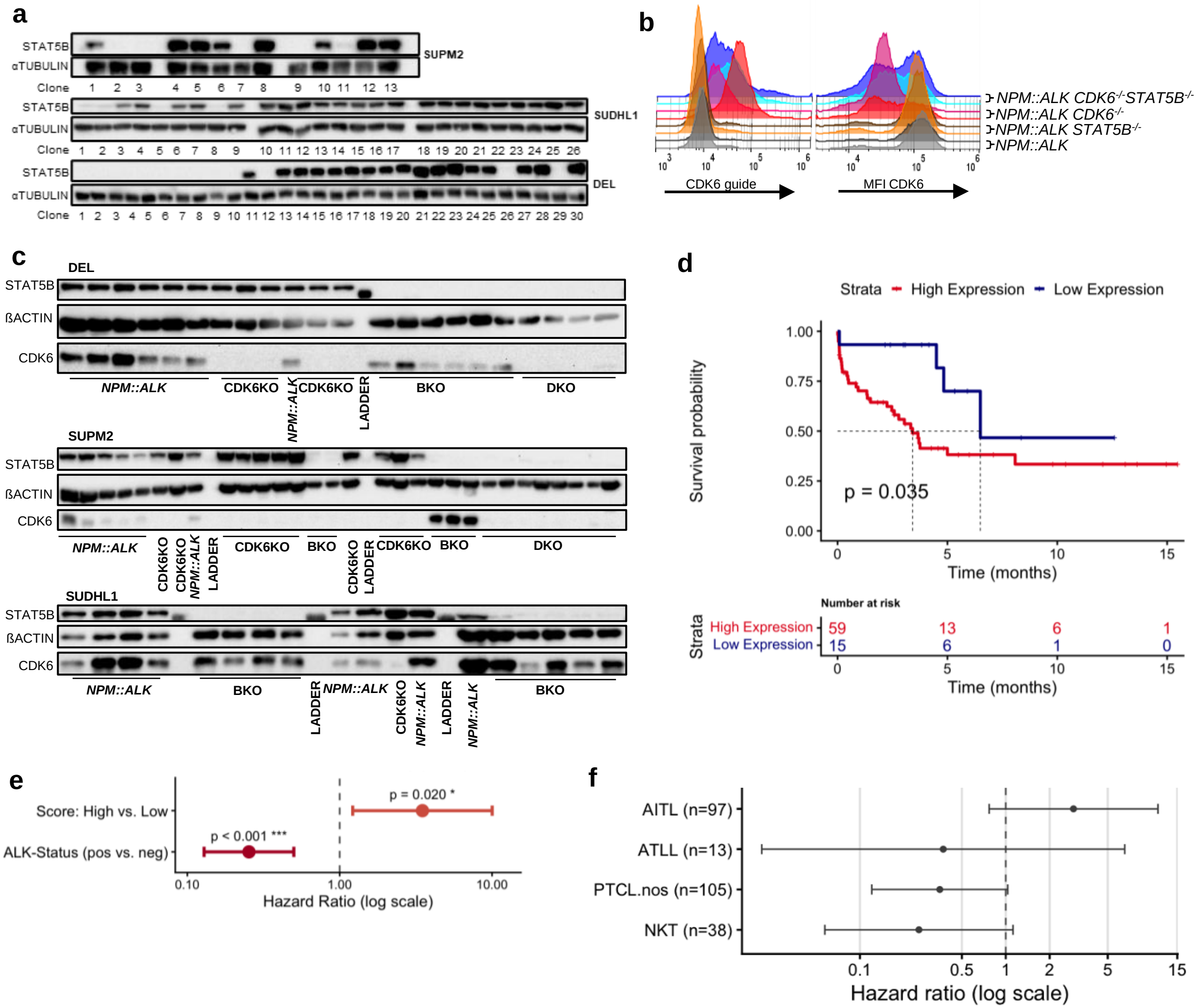
a
STAT5B
αTUBULIN
SUPM2
SUDHL1
DEL
Clone
b
NPM::ALK CDK6-/-STAT5B-/-
NPM::ALK CDK6-/-
NPM::ALK STAT5B-/-
NPM::ALK
CDK6 guide
MFI CDK6
c
DEL
SUPM2
SUDHL1
STAT5B
ßACTIN
CDK6
NPM::ALK
CDK6KO
BKO
DKO
LADDER
d
Strata
High Expression
Low Expression
Survival probability
p = 0.035
Time (months)
Number at risk
e
Score: High vs. Low
ALK-Status (pos vs. neg)
p = 0.020 *
p < 0.001 ***
Hazard Ratio (log scale)
f
AITL (n=97)
ATLL (n=13)
PTCL.nos (n=105)
NKT (n=38)
Hazard ratio (log scale)

Figure Legends of Supplementary Figures

Supplementary Figure 1: Genetic loss of *Cdk6* and *Stat5b* prevents NPM/ALK-driven tumor formation

(a) Summary of murine models analyzed. (**b**) Experimental scheme for (c-d). (**c**) (Transduction efficiency, expressed as the percentage of GFP^+ cells within the living cell population (*n* = 3 per genotype). Statistical significance was assessed by one-way ANOVA followed by Tukey's multiple comparisons test. Lines indicate the median. NPM-ALK expression levels, shown as the mean fluorescence intensity (MFI) of GFP within the GFP^+ cell population (*n* = 3 per genotype). Statistical significance was assessed by one-way ANOVA followed by Tukey's multiple comparisons test. Lines indicate the median. **(d)** Colony formation assay quantification. Bone marrow from knockout mice was transfected with an NPM-ALK plasmid and plated in growth factor-free methylcellulose. Data represent two independent experiments analyzed using a mixed-effects model (REML) followed by Tukey's multiple comparisons test. Lines indicate the median. Bars represent the mean; error bars indicate standard deviation. **(e)** Representative flow cytometry analysis plots of analysis in (f). **(f)** Major thymic T cell fractions of WT (*n* = 45), $NPM\text{-}ALK^{Tg/+}$ (*n* = 79), $Cdk6^{-/-}$ (*n* = 26), $NPM\text{-}ALK^{Tg/+}Cdk6^{-/-}$ (*n* = 37), $Stat5b^{-/-}$ (*n* = 6), $NPM\text{-}ALK^{Tg/+}Stat5b^{-/-}$ (*n* = 8), $Cdk6^{-/-}Stat5b^{-/-}$ (*n* = 15), $NPM\text{-}ALK^{Tg/+}Cdk6^{-/-}Stat5b^{-/-}$ (*n* = 11) mice; (Lin-Thy1.2$^+$CD4$^{+/-}$CD8$^{+/-}$). Significance between genotypes within each fraction was determined using two-way ANOVA with Tukey's multiple comparisons test. Bars represent the mean; error bars indicate standard deviation. **(g)** Cluster annotation of scRNA-seq showing the main cell type per cluster (color intensity) and the proportion of each Immgen cell type that originates from a given cluster (bubble size). **(h)** UMAP plots from scRNA-seq of WT, $NPM\text{-}ALK^{Tg/+}$ and $NPM\text{-}ALK^{Tg/+}Cdk6^{-/-}Stat5b^{-/-}$ DN T cells with *Cd44* and *Tnfrsf8* (*Cd30*) expression indicated. **(i)** Count heatmap of the 98 genes from Fig. 1g in ALCL patient samples. Top numbers indicate: (number of genes found in the list/total differentially expressed genes). **(j)** *Alk* expression in scRNA-seq data from WT, $NPM\text{-}ALK^{Tg/+}$ and $NPM\text{-}ALK^{Tg/+}Cdk6^{-/-}Stat5b^{-/-}$ mice excluding tumor clusters.

Supplementary Figure 2: CD26^{high} DN2/3 T cells initiate tumors and have high pSTAT5 and CDK6 levels

(a) Overlap of surface markers with up-regulated markers from human datasets from Supplementary Fig. 2e. **(b)** UMAPs indicating expression of all surface markers used in Fig. 2b, 2c, 2d. **(c)** Surface marker expression of selected markers in murine thymic lymphoma derived *NPM-ALK*$^{Tg/+}$ cell lines. **(d)** Surface marker expression of selected markers in human ALCL cell. **(e)** Gating strategy for CD26^{high} or CD26^{low} injected cells **(f)** Frequency of cycling cells (S-G2-M phase, defined as KI67+DAPI+) within each double negative thymocyte subset, assessed by flow cytometry. Each symbol represents an individual mouse and horizontal lines indicate the median. Statistical analysis was performed by ordinary two-way ANOVA with Sidak's multiple comparisons test. **(g)** Expression of TCR chains in murine cell lines *NPM-ALK*$^{Tg/+}$ (3 biological replicates), *NPM-ALK*$^{Tg/+}$*Cdk6*$^{-/-}$ (3 biological replicates) and *NPM-ALK*$^{Tg/+}$*Stat5b*$^{-/-}$(2 biological replicates). **(h)** Representative PCR analysis showing TCRβ, TCRγ and TCRδ chains in murine cell lines. **(i)** Unsupervised clustering of 4 datasets showing genes from the Datasets: Hallmark PI3K/AKT/mTOR Signaling, Biocarta MAPK signaling and KEGG JAK/STAT Signaling. **(j)** Violin plots comparing CDK6/STAT5B z-score in *Dpp4*^{high} vs *Dpp4*^{low} expressing cells (median split of *Dpp4*). Lines show median and quartile expression. Statistical significance was calculated within each genotype by ordinary one-way ANOVA with Sidak's multiple comparisons test. **(k)** Gene expression of *Cdk6*, *Stat5b* and *Dpp4* along pseudotime in WT (grey) and *NPM-ALK*$^{Tg/+}$ (red) thymocytes derived from scRNA-seq. In the background the transition of DN2b to DN3a cells is displayed. **(l)** Spearman rank correlation of DPP4 expression with CDK6 (left) and STAT5B (right) in two independent ALCL patient datasets.

Supplementary Figure 3: Developmental shift occurs at the tumor initiating stage upon CDK6 and STAT5B loss

(a) Heatmap of Immgen-based annotations for individual cells within the DN2-DN3 cluster (left) and transition cells (right). **(b)** Label transfer analysis of 6 *NPM-ALK*$^{Tg/+}$ tumor clusters to all other clusters of WT, *NPM-ALK*$^{Tg/+}$ and *NPM- ALK*$^{Tg/+}$*Cdk6*$^{-/-}$*Stat5b*$^{-/-}$. **(c)** Result of a lineage pseudotime analysis after reclustering of cells from the clusters annotated in black in Fig. 3a, connecting ETP cells to the tumor cluster. The lineage was divided into 10 equidistant intervals, with interval boundaries indicated by red dots. **(d)** Differentially expressed genes between, *NPM-ALK*$^{Tg/+}$ and *NPM-ALK*$^{Tg/+}$*Cdk6*$^{-/-}$*Stat5b*$^{-/-}$ accumulate in interval three. **(e)** The transition from DN2b to DN3a represents the most prominent cell type differentiation

event along the lineage trajectory and begins at interval 3. **(f)** Flow cytometry analysis from DN subfractions ((Lin^{-}Thy1.2^{+}CD4^{-}CD8^{-}) of WT (n = 22), *NPM-ALK*$^{Tg/+}$ (n = 23), *Cdk6*$^{-/-}$ (n = 19), *NPM-ALK*$^{Tg/+}$*Cdk6*$^{-/-}$ (n = 20), *Stat5b*$^{-/-}$ (n = 3), *NPM-ALK*$^{Tg/+}$*Stat5b*$^{-/-}$ (n = 3), *Cdk6*$^{-/-}$*Stat5b*$^{-/-}$(n = 11), *NPM-ALK*$^{Tg/+}$*Cdk6*$^{-/-}$*Stat5b*$^{-/-}$ (n = 5), *NPM-ALK*$^{Tg/+ transforming}$ (n = 25) and *NPM-ALK*$^{Tg/+}$*Cdk6*$^{-/- transforming}$ (n = 8) mice. Statistical significances between genotypes within each DN subfraction were determined using two-way ANOVA with Tukey's multiple comparisons test. **(g)** Calculated shifts from mean gene expressions per cluster throughout the trajectory between *NPM-ALK*$^{Tg/+}$ and *NPM-ALK*$^{Tg/+}$*Cdk6*$^{-/-}$*Stat5b*$^{-/-}$. **(h)** Additional plots to Fig. 3g showing large and small shifts.

Supplementary Figure 4: CDK6 and STAT5B engage an active chromatin landscape at the DN2/3 crossroad

(a) String Network (https://string-db.org/) showing high confidence (>0.7) STRING interactions from shift genes having either a CDK6 and/or a STAT5B ChIP peak in their promoter region. **(b)** Additional examples of chromatin regulators that had CDK6 and STAT5B binding in their promoters and also had an H3K27ac histone mark. **(c)** Bar graphs showing the percentage of shift genes and chromatin organization genes with a STAT5B ChIP-seq peak at their promoter (TSS ±1000 bp) in primary ALCL patient samples, ALK^{+} ALCL cell lines (SU-DHL-1, DEL, SUP-M2, Karpas-299, SR-786), and ALK^{-} ALCL cell lines (Mac-1, MAC-2a), stratified by STAT5B binding group: ALK^{+} STAT5B peaks (orange), ALK^{-} STAT5B peaks (green), or no STAT5B peak (grey). Asterisks denote two-sided Fisher's exact test versus the No STAT5B Peak reference (uncorrected). **(d)** Percentage of shift genes and chromatin organization genes with concurrent H3K27ac and STAT5B occupancy at their promoters (TSS ±1000 bp) in ALK^{-} cell lines, stratified by CDK6/STAT5B promoter occupancy (two-sided Fisher's exact test versus No Peak).

Supplementary Figure 5: CDK6 and STAT5B co-regulate transformation pathways

(a) Bubble plot illustrating deregulated transcription factor target pathways (MsigDB Transcription Factor Targets, legacy collection) with an FDR<0.1. Left panel: x-axis: NES from the comparison of Transition vs DN2b/DN3a clusters in *NPM-ALK*$^{Tg/+}$. y-axis: NES from the comparison of the transition cluster in *NPM-ALK*$^{Tg/+}$*Cdk6*$^{-/-}$*Stat5b*$^{-/-}$ vs *NPM-ALK*$^{Tg/+}$. Right panel: x-axis: NES from the comparison of Transition vs DN2b/DN3a clusters in WT. y-axis: remains as in the left panel. Bubble size represents the sum of

absolute NES values (|NES_TG| + |NES_DKOvsTG|), reflecting the combined strength of deregulation across comparisons. **(b)** Line plots showing the mean expression of core-enriched genes associated with the IL2-STAT5 signaling pathway along the pseudotime in three distinct comparisons from Fig. 4a. **(c)** Flow cytometry analysis of IL7R levels across DN1-DN4 stages in WT (*n* = 8), *NPM-ALK* $^{Tg/+}$ (*n* = 4) and *NPM-ALK*$^{Tg/+}$*Cdk6*$^{-/-}$*Stat5b*$^{-/-}$ (*n* = 4) mice. Left: Percentage of Il7R^{+} cells within the (Lin^{-}Thy1.2^{+}Cd4^{-}Cd8^{-}Cd44$^{+/-}$ Cd25$^{+/-}$) DN1-4 fractions. Right: MFI of IL7R within IL7R^{+} cells across all DN stages. Significance comparing DN1-4 per genotype within each fraction was determined using two-way ANOVA with Tukey's multiple comparisons test. **(d)** *Il2ra* expression in scRNA-seq data from WT, *NPM-ALK*$^{Tg/+}$ and *NPM-ALK*$^{Tg/+}$*Cdk6*$^{-/-}$ *Stat5b*$^{-/-}$ mice in the DN2b/DN3a cluster. **(e)** Flow cytometry analysis of IL2RA levels across DN1-DN4 stages in WT (*n* = 7), *NPM-ALK*$^{Tg/+}$ (*n* = 7) and *NPM-ALK*$^{Tg/+}$*Cdk6*$^{-/-}$*Stat5b*$^{-/-}$ (*n* = 4) mice. Mean fluorescent intensity (MFI) of IL2RA across DN2/3 stages. Significance comparing DN2-3 per genotype within each fraction was determined using two-way ANOVA with Tukey's multiple comparisons test. **(f)** Venn diagram showing STAT5B promoter peak overlaps of IL7 stimulated murine IL7 DN2 cells with ALCL cell lines. **(g)** Bubble plots displaying GSEA results (Hallmark database) from trajectory- and genotype-based comparisons. Trajectory comparisons (blue labels (I.-III.)) follow transcriptional changes along the differentiation path toward transformation, focusing on the final shared clusters across genotypes. Genotype comparisons (orange labels (III.-IV.) contrast *NPM-ALK*$^{Tg/+}$ with WT in the last two shared differentiation stages. Only pathways consistently deregulated (FDR<0.1) across both comparisons are shown. Bubble size reflects the -$\log_{10}$(FDR), and color indicates the normalized enrichment score (NES), with red denoting positive enrichment and blue negative enrichment. **(h)** Flow Cytometry of living cell (Annexin V^{-}, DAPI^{-}) percentage in DN1-DN3 thymocytes (Lin^{-}Thy1.2^{+}CD4^{-}CD8^{-}CD44$^{+/-}$CD25$^{+/-}$). Frequencies were quantified in WT (*n* = 8), *NPM-ALK*$^{Tg/+}$ (*n* = 4) and *NPM-ALK* $^{Tg/+}$*Cdk6*$^{-/-}$*Stat5b*$^{-/-}$(*n* = 4) mice. Two-way ANOVA with Tukey's multiple comparisons test was performed, corresponding to plot Fig 4C. **(i)** Western blot of STAT5B IP in a human ALCL cell line (SU-DHL-1) +/- crosslinker DSSO. IgG was the negative control. **(j)** Cell cycle distribution of SU-DHL-1 and DEL cells (combined, mean ± SD) after 18 h 200 nM palbociclib treatment. **(k)** IP-MS CDK6 interactors in SU-DHL-1 vs DEL cells, untreated (left) and palbociclib-treated (right). Stable interactors (*n* = 129, blue) and gained interactors (*n* = 6, dark green)

are highlighted; dashed lines indicate linear regression fits. STRING GO enrichment of the 129 stable interactors: **(l)** Biological Process, **(m)** Molecular Function.

Supplementary Figure 6: Reciprocal pathway activation is observed in the absence of CDK6 or STAT5B

(a) Classification of pathway overlaps based on GSEA across selected databases (GO biological processes, GO molecular functions, Hallmark, KEGG, PID, Reactome, Transcription factor targets legacy and WikiPathways). Pathways are grouped into three regulatory categories: UP-regulated overlaps (blue): pathways up-regulated in both of any two comparisons; CROSS-regulated overlaps (red): pathways oppositely regulated (Colored arrows(blue=up or purple=down) next to cross-regulated pathways indicate the direction of change in the respective comparison); DOWN-regulated overlaps (purple) indicate pathways down regulated in both of any comparisons. **(b)** Histogram overlay plot of intracellular flow cytometry analysis showing CDK6 levels after 6 h CDK6 PROTAC degrader BSJ-03-123 treatment , compared to DMSO (vehicle control) in 5 different murine *NPM-ALK*$^{Tg/+}$ cell lines. **(c)** MFI of CDK6 shown (b). Statistical analysis was performed using an unpaired two-tailed t-test. **(d)** Representative Western blot analysis of CDK6 protein levels in cell lines treated for 6 h with either DMSO or the CDK6 degrader BSJ-03-123 at 0.3 µM or 3 µM. αTUBULIN served as loading control. The bar graph on the right shows quantification of CDK6 signal normalized to αTUBULIN and expressed as log2 fold change relative to the corresponding DMSO control of *NPM-ALK*$^{Tg/+}$ cells. Data are presented as mean with standard deviation. Statistical analysis was performed by ordinary two-way ANOVA with Sidak's multiple comparisons test. **(e)** Representative Western blot analysis of phosphorylated Rb at Ser780 (pRb(S780)) in murine cell lines treated for 24 h with either H_2O (vehicle) or 250 nM Palbociclib. GAPDH served as loading control. The bar graph on the right shows quantification of pRb(S780) signal normalized to GAPDH and expressed as log2 fold change relative to the H_2O control of *NPM-ALK*$^{Tg/+}$ cells. Lanes of *Stat5b* and *Cdk6* deficient lines are shown as qualitative controls. Data are presented as mean with standard deviation. Statistical analysis was performed by a two-tailed paired t-test. **(f)** Western blot analysis showing total STAT5A/B levels after treatment with STAT5 PROTAC degrader AK-2292 in three murine cell lines after 24, 48 and 72 h.

Supplementary Figure 7: Dual deletion of CDK6 and STAT5B induces synthetic lethality in human ALCL cells in a context dependent manner

(a) Representative western blot analysis of STAT5B levels in single cell clones from 3 human ALCL cell lines. Numbers represent tested clones per cell line. **(b)** Representative histogram overlay plot of intracellular CDK6 staining showing tracrRNA/guide expression after six days post electroporation (left) and CDK6 levels in the same cells (right). **(c)** Representative Western blot analysis of CDK6KO and DKO cell lines confirming the loss of CDK6 and/or STAT5B. **(d)** Kaplan–Meier overall survival curves for the combined cohort ($n = 74$) stratified by high (red) versus low (blue) signature expression. The optimal cutpoint was determined using the maximally selected rank statistic (maxstat, log-rank scoring, Lau92 p-value approximation). **(e)** Forest plot of the multivariate Cox model in the combined cohort with the signature dichotomized as high vs. low (HR = 3.49, 95% CI: 1.22–10.03, $p = 0.020$) and ALK status (positive vs. negative; HR = 0.25, 95% CI: 0.13–0.50, $p < 0.001$) as covariates; concordance index = 0.71. **(f)** Forest plot of HR from univariate Cox proportional hazards models fitted independently within each subtype, with the per sample standardized gene set score as the sole covariate. Each row corresponds to one PTCL molecular subtype; sample sizes are given in parentheses next to the subtype label. Points indicate HR point estimates and horizontal bars span the 95% Wald confidence interval. The hazard ratio axis is shown on a base 10 logarithmic scale.
