# Supplementary material for: Ablation of CDK6 and STAT5B rewires T cell developmental dynamics and prevents ALK+ lymphoma formation
Source: Nat Commun. 2026 Sep 15;17:10853. doi: 10.1038/s41467-026-77466-4 (PMC13672290; doi:10.1038/s41467-026-77466-4)
Supplement: Supplementary file 2 [file 41467_2026_77466_MOESM2_ESM.pdf]

nature portfolio

Corresponding author(s): Karoline Kollmann and Thorsten Klampfl

Last updated by author(s): Oct 31, 2025

Reporting Summary

Nature Portfolio wishes to improve the reproducibility of the work that we publish. This form provides structure for consistency and transparency in reporting. For further information on Nature Portfolio policies, see our Editorial Policies and the Editorial Policy Checklist.

Statistics

For all statistical analyses, confirm that the following items are present in the figure legend, table legend, main text, or Methods section.

n/a	Confirmed	
☐	☒	The exact sample size (*n*) for each experimental group/condition, given as a discrete number and unit of measurement
☐	☒	A statement on whether measurements were taken from distinct samples or whether the same sample was measured repeatedly
☐	☒	The statistical test(s) used AND whether they are one- or two-sided<br>*Only common tests should be described solely by name; describe more complex techniques in the Methods section.*
☐	☒	A description of all covariates tested
☐	☒	A description of any assumptions or corrections, such as tests of normality and adjustment for multiple comparisons
☐	☒	A full description of the statistical parameters including central tendency (e.g. means) or other basic estimates (e.g. regression coefficient) AND variation (e.g. standard deviation) or associated estimates of uncertainty (e.g. confidence intervals)
☐	☒	For null hypothesis testing, the test statistic (e.g. *F*, *t*, *r*) with confidence intervals, effect sizes, degrees of freedom and *P* value noted<br>*Give P values as exact values whenever suitable.*
☒	☐	For Bayesian analysis, information on the choice of priors and Markov chain Monte Carlo settings
☒	☐	For hierarchical and complex designs, identification of the appropriate level for tests and full reporting of outcomes
☐	☒	Estimates of effect sizes (e.g. Cohen's *d*, Pearson's *r*), indicating how they were calculated

Our web collection on statistics for biologists contains articles on many of the points above.

Software and code

Policy information about availability of computer code

Data collection

Flow cytometry data were collected using CytExpert (Version 2.4.0.28, Beckman Coulter) or CytExpert SRT (Version 1.1.0.10007, Beckman Coulter). Luminescence data were acquired using the SkanIt™ Software (Thermo Fisher Scientific).

Data analysis

Single-cell RNA sequencing raw data were processed using the CellRanger v. 7.0.0 “count” program to generate raw feature-barcode-matrix data. Further analysis was performed in R (version 4.3.1 or higher) using publically available packages including “DropletUtils”, “scater”, “batchelor”, “uwot”, “bluster”, “scuttle”, and “scran”, for QC, batch correction, dimensionality reduction and clustering, “SingleR” for cell type annotation, “TSCAN” for trajectory and pseudotime analysis, and “Seurat” for differential gene expression and label transfer.

RNA sequencing raw reads were quality controlled using Fastqc (version 0.12.1). Adapters and low-quality reads were trimmed using Trimmomatic (version 0.39).The reads were aligned against reference genomes using STAR (version 2.7.6a). QC of the alignments was done with Qualimap (version 2.2.2a). Reads overlapping exons were counted with the FeatureCounts program of the Subread package (version 2.0.1) and summarized as total counts per gene. Differential gene expression analysis was performed in R (version 4.3.1) using the DESeq2 (version 1.40.2) package.

ChIP sequencing analysis: FASTQC software (version 0.12.1) was used for quality control, Trimmomatic (version 0.39) was used to perform adapter trimming and quality-based filtering of reads. Murine reads were mapped against the GRCm38 primary assembly (Gencode M25), reads from human samples against the GRCh38 primary assembly (Gencode v26) using bwa-mem (version 0.7.15). Alignment QC was done with Qualimap (version 2.2.2a). Blacklisted regions were removed with bedtools subtract. Multimappers and reads with low mapping quality were removed using samtools (version 1.14). We used MACS3 to perform peak calling with default parameters. For each ChIP-seq file we used the corresponding Input file as the control. The resulting “narrowPeak” files were annotated using R (version 4.3.1) and the “GenomicFeatures” and “GenomicRanges” packages.

Gene set enrichment analyses were performed with the Broad Institute's GSEA software (v4.3.2) or STRING (v12.0; https://string-db.org/, accessed 17.03.2025)

Flow cytometry analyses were performed using CytExpert (Version 2.4.0.28, Beckman Coulter), CytExpert SRT (Version 1.1.0.10007, Beckman Coulter) or FlowJo (Version 10.8.-10.10.)

For manuscripts utilizing custom algorithms or software that are central to the research but not yet described in published literature, software must be made available to editors and reviewers. We strongly encourage code deposition in a community repository (e.g. GitHub). See the Nature Portfolio guidelines for submitting code & software for further information.

Data

Policy information about availability of data

All manuscripts must include a data availability statement. This statement should provide the following information, where applicable:

- Accession codes, unique identifiers, or web links for publicly available datasets
- A description of any restrictions on data availability
- For clinical datasets or third party data, please ensure that the statement adheres to our policy

Murine scRNA-seq data have been uploaded to ArrayExpress under accession: E-MTAB-15478. Human and murine ChIP-seq data are available under E-MTAB-15481, and human and murine bulk RNA-seq data under E-MTAB-15486.
The data can be accessed using these links:
scRNA seq: https://www.ebi.ac.uk/biostudies/arrayexpress/studies/E-MTAB-15478
ChIP seq: https://www.ebi.ac.uk/biostudies/arrayexpress/studies/E-MTAB-15481
RNA seq: https://www.ebi.ac.uk/biostudies/arrayexpress/studies/E-MTAB-15486

Publicly available datatsets analyzed in this study include microarray data (GSE14879, GSE19069, GSE65823, GSE78513), human ALCL scRNA-seq (SRR24860075, SRR24860077), RNA-seq from ALCL patients (E-MTAB-2770, HRA004246, PRJNA255877), DN ChIP-seq data (GSE283392), H3K27ac ChIP-seq data (GSE158916) and GSE58445, GSE19069 (both generated on the Affymetrix Human Genome U133 Plus 2.0 platform, GPL570) and E-TABM-117 data from gene expression microarrays with survival information (E-TABM-117).

Research involving human participants, their data, or biological material

Policy information about studies with human participants or human data. See also policy information about sex, gender (identity/presentation), and sexual orientation and race, ethnicity and racism.

Reporting on sex and gender	This study did not involve human participants.
Reporting on race, ethnicity, or other socially relevant groupings	This study did not involve human participants.
Population characteristics	This study did not involve human participants.
Recruitment	This study did not involve human participants.
Ethics oversight	Not applicable.

Note that full information on the approval of the study protocol must also be provided in the manuscript.

Field-specific reporting

Please select the one below that is the best fit for your research. If you are not sure, read the appropriate sections before making your selection.

☒ Life sciences ☐ Behavioural & social sciences ☐ Ecological, evolutionary & environmental sciences

For a reference copy of the document with all sections, see nature.com/documents/nr-reporting-summary-flat.pdf

Life sciences study design

All studies must disclose on these points even when the disclosure is negative.

Sample size	No statistical methods were used to predetermine sample size. The number of animals used was based on genotype availability and experimental feasability. The chosen sample sizes are consistent with standards commonly used in the field and allowed robust detection of biological effects.
Data exclusions	Data were excluded only when clear experimental or technical errors were identified (e.g. failed genotyping, injection errors, or technical failure in downstream assays). All exclusion criteria were predefined and applied consistently across experiments.
Replication	All experiments were independently repeated as indicated in figure legends and methods sections. Replicate numbers are specified for each

Replication	experiment and all attempts at replication were successful unless stated otherwise.
Randomization	Mice were randomly assigned to experimental groups, this includes injections into NSG recipients to avoid allocation bias. Randomization was performed based on cage lists and genotype availability.
Blinding	During experiments, mice were identified only by ID numbers. Genotypes were revealed after data collection and analysis were completed.

Behavioural & social sciences study design

All studies must disclose on these points even when the disclosure is negative.

Study description	*Briefly describe the study type including whether data are quantitative, qualitative, or mixed-methods (e.g. qualitative cross-sectional, quantitative experimental, mixed-methods case study).*
Research sample	*State the research sample (e.g. Harvard university undergraduates, villagers in rural India) and provide relevant demographic information (e.g. age, sex) and indicate whether the sample is representative. Provide a rationale for the study sample chosen. For studies involving existing datasets, please describe the dataset and source.*
Sampling strategy	*Describe the sampling procedure (e.g. random, snowball, stratified, convenience). Describe the statistical methods that were used to predetermine sample size OR if no sample-size calculation was performed, describe how sample sizes were chosen and provide a rationale for why these sample sizes are sufficient. For qualitative data, please indicate whether data saturation was considered, and what criteria were used to decide that no further sampling was needed.*
Data collection	*Provide details about the data collection procedure, including the instruments or devices used to record the data (e.g. pen and paper, computer, eye tracker, video or audio equipment) whether anyone was present besides the participant(s) and the researcher, and whether the researcher was blind to experimental condition and/or the study hypothesis during data collection.*
Timing	*Indicate the start and stop dates of data collection. If there is a gap between collection periods, state the dates for each sample cohort.*
Data exclusions	*If no data were excluded from the analyses, state so OR if data were excluded, provide the exact number of exclusions and the rationale behind them, indicating whether exclusion criteria were pre-established.*
Non-participation	*State how many participants dropped out/declined participation and the reason(s) given OR provide response rate OR state that no participants dropped out/declined participation.*
Randomization	*If participants were not allocated into experimental groups, state so OR describe how participants were allocated to groups, and if allocation was not random, describe how covariates were controlled.*

Ecological, evolutionary & environmental sciences study design

All studies must disclose on these points even when the disclosure is negative.

Study description	*Briefly describe the study. For quantitative data include treatment factors and interactions, design structure (e.g. factorial, nested, hierarchical), nature and number of experimental units and replicates.*
Research sample	*Describe the research sample (e.g. a group of tagged Passer domesticus, all Stenocereus thurberi within Organ Pipe Cactus National Monument), and provide a rationale for the sample choice. When relevant, describe the organism taxa, source, sex, age range and any manipulations. State what population the sample is meant to represent when applicable. For studies involving existing datasets, describe the data and its source.*
Sampling strategy	*Note the sampling procedure. Describe the statistical methods that were used to predetermine sample size OR if no sample-size calculation was performed, describe how sample sizes were chosen and provide a rationale for why these sample sizes are sufficient.*
Data collection	*Describe the data collection procedure, including who recorded the data and how.*
Timing and spatial scale	*Indicate the start and stop dates of data collection, noting the frequency and periodicity of sampling and providing a rationale for these choices. If there is a gap between collection periods, state the dates for each sample cohort. Specify the spatial scale from which the data are taken*
Data exclusions	*If no data were excluded from the analyses, state so OR if data were excluded, describe the exclusions and the rationale behind them, indicating whether exclusion criteria were pre-established.*
Reproducibility	*Describe the measures taken to verify the reproducibility of experimental findings. For each experiment, note whether any attempts to repeat the experiment failed OR state that all attempts to repeat the experiment were successful.*
Randomization	*Describe how samples/organisms/participants were allocated into groups. If allocation was not random, describe how covariates were controlled. If this is not relevant to your study, explain why.*

Blinding | Describe the extent of blinding used during data acquisition and analysis. If blinding was not possible, describe why OR explain why blinding was not relevant to your study.

Did the study involve field work? ☐ Yes ☐ No

Field work, collection and transport

Field conditions | Describe the study conditions for field work, providing relevant parameters (e.g. temperature, rainfall).

Location | State the location of the sampling or experiment, providing relevant parameters (e.g. latitude and longitude, elevation, water depth).

Access & import/export | Describe the efforts you have made to access habitats and to collect and import/export your samples in a responsible manner and in compliance with local, national and international laws, noting any permits that were obtained (give the name of the issuing authority, the date of issue, and any identifying information).

Disturbance | Describe any disturbance caused by the study and how it was minimized.

Reporting for specific materials, systems and methods

We require information from authors about some types of materials, experimental systems and methods used in many studies. Here, indicate whether each material, system or method listed is relevant to your study. If you are not sure if a list item applies to your research, read the appropriate section before selecting a response.

Materials & experimental systems

n/a	Involved in the study	
☐	☒	Antibodies
☐	☒	Eukaryotic cell lines
☒	☐	Palaeontology and archaeology
☐	☒	Animals and other organisms
☒	☐	Clinical data
☒	☐	Dual use research of concern
☒	☐	Plants

Methods

n/a	Involved in the study	
☐	☒	ChIP-seq
☐	☒	Flow cytometry
☒	☐	MRI-based neuroimaging

Antibodies

Antibodies used

Chip-Seq:
anti-CDK6 (rabbit polyclonal, PA5-27978; Invitrogen, USA), anti-IgG (rabbit polyclonal, 02-6102; Invitrogen, USA)
STAT5b ([EPR16671], Abcam, ab178941) antibody, IgG isotype control ([EPR25A], Abcam, ab172730)

Immunoblotting:
Membranes were probed with primary antibodies at a 1:1000 dilution in 3% BSA in py-TBST, unless stated otherwise. The used antibodies were directed against αTubulin (DM1A) (Santa Cruz Biotechnology, Inc., Sc-32293), STAT5b (abcam, 178941, 1:2000), STAT5 (BD Biosciences, BD610192, 1:250), CDK6 (Invitrogen, PA5-27978), ACTB (Santa Cruz Biotechnology, Inc., Sc69879, 1:3000), pRb s780 (Cell Signaling Technology, CS9307S), GAPDH (ABS16, Merck Millipore) HSC70 (sc-7298, Santa Cruz biotechnology, 1:10000). Secondary HRP antibodies: (anti-mouse IgG, HRP-linked Antibody (cs7076, Cell Signaling Technology, 1:7500), anti-rabbit IgG, HRP-linked Antibody (cs7074, Cell Signaling Technology, 1:7500)) .

Immunoprecipitation:
anti-CDK6 (rabbit polyclonal, PA5-27978; Invitrogen, USA), anti-IgG (rabbit polyclonal, 02-6102; Invitrogen, USA)
STAT5b ([EPR16671], Abcam, ab178941) antibody, IgG isotype control ([EPR25A], Abcam, ab172730)

Flow Cytometry:
Fluorochrome-conjugated antibodies for the detection of the following proteins, also for surface marker screenings in human and murine cell lines, were used from:

Biozym/BioLegend:
CD4 BV650 (GK1.5), CD8a BV606 (53-6.7), CD26 APC (H194-112), CD44 PerCP (IM7), Gr-1 APC-Cy7 (RB6-8C5), TER-119 APC-Cy7 (TER-11), CD26 PE (H194-112), CD274 APC (B7-H1, PD-L1; 29E.2A3), CD54 PE (HA58), CD26 PE (BA5b), CD107a PE (H4A3), HLA-A,B,C PE (W6/32), CD135 PE (BV10A4H2), CD11b APC (ICRF44), CD90 PE-Cy5 (5E10), KLRG1 APC (2F1/KLRG1), CD24 FITC (ML5), CD120b APC (3G7A02), CD120a APC (W15099A), CD120a APC (55R-286), CD38 PE-Cy7 (HB-7), CD34 PE (581), CD48 PE (HM48-1), CD274 BV421 (10F.9G2), LY49A PE (A1/Ly49A), TCRB APC (H57-597), CD90.1 APC (OX-7/OX7), CD127 Alexa Fluor488 (A7R34), CD3 PB (17A2), CD226 APC (10E5), CD326 APC-Cy7 (G8.8), CD94 APC (18d3), CD126 APC (D7715A7), TCRGD BV605 (GL3), Streptavidin PECy7 (1:1000), Thy1.2 BV510 (53-2.1);

BD Biosciences:
CD19 FITC (1D3), CD11B FITC (M1/70), Alexa Fluor647 CD61(2C9.G2 (HMß3-1)), CD11b APC-Cy7 (M1/70), CD25 PE-Cy7 (PC61), CD19 APC-Cy7 (1D3), CD24 FITC (M1/69), CD63 FITC (H5C6), CD43 PE (S7), CD62L APC-Cy7 (MEL14), SCA1 PE-Cy7 (D7), CD5 PE (53-7.3), CD31 FITC (MEC13.3), CD34 FITC (RAM34), CD19 PE (1D3), LY6G/C FITC (RB6-8C5) CD107a FITC (1D4B), TCRA FITC (B20.1), CD4 FITC (GK1.5), CD335 FITC (29A1.4), CD44 APC-Cy7 IM7), pSTAT5-PB450 (47/Stat5(pY694) (1:6)), Ki-67 FITC (1:10; B56), CD25 PE (3C7), CD8 APC (53-6.7);

eBioscience/FisherScientific:
CD44-PE (IM7), CD127 (IL7R eFluor450 (A7R34)), CD9 FITC (SN4 C3-3A2), CD45 FITC (HI30), MHC Class I (H-2Db) PE (28-14-8), MHC Class II (I-A/I-E) FITC (M5/114.15.2), CD61 PE (2C9.G3), CD16/32 PE (93), CD140b PE (93), CD244.2 PE (eBio244F4), CD117 APC (2B8), CD51 PE (RMV-7), CD150 FITC (9D1), CD123 PE (5B11), CD253 PE (N2B2), CD135 PE (A2F10), CD215 APC (DNT15Ra), CD49b APC-Cy7 (DX5), NK1.1 eFluor 450 (PK136), CD197 eFluor450 (4B12), CD122 FITC (TM-b1), CD127 FITC (A7R34), purified anti-CD16/CD32 antibodies (clone 93);

Invitrogen/FisherScientific: CD25 Biotin (7D49), GR1 FITC (RB6-8C5), IgG (H+L) Cross-Adsorbed Secondary Antibody Alexa Fluor647 (1:500), CD45 FITC (30-F11), CD71 FITC (R17217), CD9 eFluor450 (KMC8), CD144 eFluor660 (eBioBV13), KLRG1 FITC (2F1), CD93 APC (AA4.1), CD314 APC (CX5), CD4 PE-Cy7 (GK1.5).
If not stated otherwise here or in the methods for each experiment, flow cytometry antibodies were used at a 1:200 dilution.

Validation

If not validated by manufacturer or prior publications, we validated antibodies based on expected staining patterns in positive and negative controls, use of single stains, FMOs or isotype controls and reproducibility across independent experiments. All ChIP antibodies were extensively validated using knockout (KO) controls, immunoblotting, immunocytochemistry (ICC), and immunohistochemistry (IHC). These antibodies were also successfully used and validated in previous studies from our laboratory (Kollmann et al., Blood 2021; Mayer et al., Blood 2024).

Eukaryotic cell lines

Policy information about cell lines and Sex and Gender in Research

Cell line source(s)

Upon reaching endpoint criteria, thymic lymphomas of NPM-ALK Tg/+, NPM-ALK Tg/+Cdk6-/- and NPM ALK Tg/+Stat5b-/- mice were processed into single-cell suspensions using 40-70 µm strainers. Cells were cultured in RPMI 1640 (Sigma) medium supplemented with 10% heat inactivated fetal calf serum (Gibco, Fisher Scientific), 50 µM β-mercaptoethanol (Sigma), and 100 U/mL penicillin, 100 µg/mL streptomycin (Sigma) at 37°C and 5% CO2. Cells were monitored microscopically until outgrowth and stable proliferation and were then maintained under these conditions for all subsequent experiments. SUP-M2 cells were a kind gift from Olaf Merkel (AKH, Vienna), SU-DHL-1, Mac-1, and Mac-2A were provided by Richard Moriggl, and DEL and KI-JK cells were obtained from DSMZ.

Authentication

DEL and KI-JK cells were obtained from DSMZ and not separately authenticated. SUP-M2 cells were authenticated by our collaborator. DEL, SU-DHL-1, and SUP-M2 cells used in the CRISPR knock-out experiment were authenticated by STR profiling.

Mycoplasma contamination

All cell lines were routinely testet for mycoplasma after thawing and before experiments using the abm G238 Mycoplasma PCR Detection Kit. All tests were negative.

Commonly misidentified lines
(See ICLAC register)

We did not use any commonly misidentified cell lines for our experimental studies.

Palaeontology and Archaeology

Specimen provenance

Provide provenance information for specimens and describe permits that were obtained for the work (including the name of the issuing authority, the date of issue, and any identifying information). Permits should encompass collection and, where applicable, export.

Specimen deposition

Indicate where the specimens have been deposited to permit free access by other researchers.

Dating methods

If new dates are provided, describe how they were obtained (e.g. collection, storage, sample pretreatment and measurement), where they were obtained (i.e. lab name), the calibration program and the protocol for quality assurance OR state that no new dates are provided.

☐ Tick this box to confirm that the raw and calibrated dates are available in the paper or in Supplementary Information.

Ethics oversight

Identify the organization(s) that approved or provided guidance on the study protocol, OR state that no ethical approval or guidance was required and explain why not.

Note that full information on the approval of the study protocol must also be provided in the manuscript.

Animals and other research organisms

Policy information about studies involving animals; ARRIVE guidelines recommended for reporting animal research, and Sex and Gender in Research

Laboratory animals

NPM-ALK Tg/+ mice (Tg(Cd4-NPM/ALK)N1Ingh) (Chiarle, R. et al., Blood, 2003) and previously described NPM-ALK Tg/+Cdk6-/- mice

Laboratory animals	(Tg(Cd4 NPM/ALK)N1inghCdk6tm1Bbd) (Kollmann, K. et al., Cancer Cell, 2013) were crossed with Stat5b-/- mice (Stat5btmHwd) (Udy, G. B. et al. PNAS, 1997) to generate NPM ALK Tg/+Stat5b / and NPM-ALK Tg/+Cdk6-/-Stat5b-/- mice. All strains were maintained on a C57BL/6 background. Genotypes were confirmed by PCR analysis. Most experiments were carried out with mice around 10 weeks of age. The age range is specified in figure legends. All mice, including NSG mice (NOD.Cg-Prkdcscid Il2rgtm1Wjl/SzJ; The Jackson Laboratory) were maintained at the rodent facility of the University of Veterinary Medicine Vienna. Specific pathogen-free (SPF) conditions were frequently confirmed according to FELASA recommendations for health monitoring.
Wild animals	The study did not involve wild animals.
Reporting on sex	Both male and female mice were used. No sex-specific effects were observed or expected and data from both sexes were combined for analysis.
Field-collected samples	The study did not involve samples collected from the field.
Ethics oversight	All breeding pairs and planned experiments are approved by the Ethics and Animal Welfare Committee of the University of Veterinary Medicine, Vienna in accordance with the University's guidelines for Good Scientific Practice and authorized by the Austrian Federal Ministry of Education, Science and Research in accordance with current legislation, under licenses BMBWF-68.205/0174-V/3b/2018, BMWFW-68.205/0093-WF/V/3b/2015, 2022-0.404.452, BMBWF-68.205/0094-WF/V/3b/2015, 2024 0.826.297.

Note that full information on the approval of the study protocol must also be provided in the manuscript.

Clinical data

Policy information about clinical studies

All manuscripts should comply with the ICMJE guidelines for publication of clinical research and a completed CONSORT checklist must be included with all submissions.

Clinical trial registration	*Provide the trial registration number from ClinicalTrials.gov or an equivalent agency.*
Study protocol	*Note where the full trial protocol can be accessed OR if not available, explain why.*
Data collection	*Describe the settings and locales of data collection, noting the time periods of recruitment and data collection.*
Outcomes	*Describe how you pre-defined primary and secondary outcome measures and how you assessed these measures.*

Dual use research of concern

Policy information about dual use research of concern

Hazards

Could the accidental, deliberate or reckless misuse of agents or technologies generated in the work, or the application of information presented in the manuscript, pose a threat to:

No	Yes	
☒	☐	Public health
☒	☐	National security
☒	☐	Crops and/or livestock
☒	☐	Ecosystems
☒	☐	Any other significant area

Experiments of concern

Does the work involve any of these experiments of concern:

No	Yes	
☒	☐	Demonstrate how to render a vaccine ineffective
☒	☐	Confer resistance to therapeutically useful antibiotics or antiviral agents
☒	☐	Enhance the virulence of a pathogen or render a nonpathogen virulent
☒	☐	Increase transmissibility of a pathogen
☒	☐	Alter the host range of a pathogen
☒	☐	Enable evasion of diagnostic/detection modalities
☒	☐	Enable the weaponization of a biological agent or toxin
☒	☐	Any other potentially harmful combination of experiments and agents

Plants

Seed stocks

Report on the source of all seed stocks or other plant material used. If applicable, state the seed stock centre and catalogue number. If plant specimens were collected from the field, describe the collection location, date and sampling procedures.

Novel plant genotypes

Describe the methods by which all novel plant genotypes were produced. This includes those generated by transgenic approaches, gene editing, chemical/radiation-based mutagenesis and hybridization. For transgenic lines, describe the transformation method, the number of independent lines analyzed and the generation upon which experiments were performed. For gene-edited lines, describe the editor used, the endogenous sequence targeted for editing, the targeting guide RNA sequence (if applicable) and how the editor was applied.

Authentication

Describe any authentication procedures for each seed stock used or novel genotype generated. Describe any experiments used to assess the effect of a mutation and, where applicable, how potential secondary effects (e.g. second site T-DNA insertions, mosiacism, off-target gene editing) were examined.

ChIP-seq

Data deposition

☒ Confirm that both raw and final processed data have been deposited in a public database such as GEO.

☒ Confirm that you have deposited or provided access to graph files (e.g. BED files) for the called peaks.

Data access links
May remain private before publication.

https://www.ebi.ac.uk/biostudies/arrayexpress/studies/E-MTAB-15481?key=cc19b8e1-01fd-4d1c-86a1-09a814c5092f

Files in database submission

273912_S27_R1_001_20240426.fastq.gz
273912_S64_R1_001.fastq.gz
273928_S43_R1_001.fastq.gz
273928_S43_R1_001_20240426.fastq.gz
273928_S48_R1_001.fastq.gz
273934_S42_R1_001.fastq.gz
273934_S49_R1_001.fastq.gz
273934_S49_R1_001_20240426.fastq.gz
273935_S41_R1_001.fastq.gz
273935_S50_R1_001.fastq.gz
273935_S50_R1_001_20240426.fastq.gz
273936_S40_R1_001.fastq.gz
273936_S51_R1_001.fastq.gz
273936_S51_R1_001_20240426.fastq.gz
273937_S39_R1_001.fastq.gz
273937_S52_R1_001.fastq.gz
273937_S52_R1_001_20240426.fastq.gz
273938_S38_R1_001.fastq.gz
273938_S53_R1_001.fastq.gz
273938_S53_R1_001_20240426.fastq.gz
273948_S28_R1_001.fastq.gz
273948_S63_R1_001.fastq.gz
273948_S63_R1_001_20240426.fastq.gz
273949_S27_R1_001.fastq.gz
273949_S64_R1_001.fastq.gz
273949_S64_R1_001_20240426.fastq.gz
273950_S26_R1_001.fastq.gz
273950_S65_R1_001.fastq.gz
273950_S65_R1_001_20240426.fastq.gz
273951_S25_R1_001.fastq.gz
273951_S66_R1_001.fastq.gz
273951_S66_R1_001_20240426.fastq.gz
273952_S24_R1_001.fastq.gz
273952_S67_R1_001.fastq.gz
273952_S67_R1_001_20240426.fastq.gz
CDK6_417_CDK6_WT_peaks.narrowPeak
Stat5_DEL_Stat5_peaks.narrowPeak
Stat5_K299_Stat5_peaks.narrowPeak
Stat5_SR786_Stat5_peaks.narrowPeak
Stat5_SUDHL1_Stat5_peaks.narrowPeak
Stat5_SUPM2_Stat5_peaks.narrowPeak

Genome browser session
(e.g. UCSC)

Link to genome browser session of human ChIP seq data:
http://genome.ucsc.edu/cgi-bin/hgTracks?hgS_doLoadUrl=submit&hgS_loadUrlName=https://www.dropbox.com/scl/fi/1macm26woegede0hla4sr/human_ChIPseq_tracks_session_file?rlkey=5mvrs7y9dtpdixi0vdubfshju&st=1iliwzce&dl=0

Link to genome browser session of mouse ChIP seq data:
http://genome.ucsc.edu/cgi-bin/hgTracks?hgS_doLoadUrl=submit&hgS_loadUrlName=https://www.dropbox.com/scl/fi/7v93o27qwx9yws0qt93oa/mouse_ChIPseq_tracks_session_file?rlkey=yanrbxm7b071dr3m4dkclwj7d&st=2lzr939y&dl=0

Methodology

Replicates

Stat5 binding was assessed in five human ALCL cell lines. CDK6 binding was assessed in a single mouse cell line.

Sequencing depth

All samples were sequenced in a 100 bp single-end fashion.

sample (number of raw reads, number of uniquely aligned reads)
CDK6_417_CDK6_WT (24170674, 3756766)
CDK6_417_Input_WT (14279037, 5485451)
Stat5_DEL_Input (8194930, 4493169)
Stat5_DEL_Stat5 (5680294, 2645455)
Stat5_K299_Input (11313688, 7249693)
Stat5_K299_Stat5 (35007566, 25020367)
Stat5_SR786_Input (11224800, 6644710)
Stat5_SR786_Stat5 (4370443, 2290102)
Stat5_SUDHL1_Input (11129139, 7845506)
Stat5_SUDHL1_Stat5 (4911360, 3081731)
Stat5_SUPM2_Input (8205808, 4636834)
Stat5_SUPM2_Stat5 (22759916, 17262869)

Antibodies

anti-CDK6 (rabbit polyclonal, PA5-27978; Invitrogen, USA), anti-IgG (rabbit polyclonal, 02-6102; Invitrogen, USA)
STAT5b antibody ([EPR16671], Abcam, ab178941), IgG isotype control ([EPR25A], Abcam, ab172730)

Peak calling parameters

MACS3 was used for peak calling with the following command:
macs3 callpeak -t <treatment.bam> -c <control.bam> -g <mm or hs> -n <output_file_prefix> --outdir <output directory>
where <treatment.bam> is the alignment file of the ChIP'ed sample and <control.bam> is the alignment file of the corresponding Input sample

Data quality

Data quality was assessed during read processing (initial read QC, adapter trimming and removal of low quality reads, trimmed reads QC, alignment QC). The final peak numbers were:
sample (number of peaks above 5-fold enrichment, number of peaks at FDR 5%)
CDK6_417_CDK6_WT (7893, 10153)
Stat5_K299_Stat5 (12268, 14173)
Stat5_SR786_Stat5 (14690, 18899)
Stat5_SUDHL1_Stat5 (1392, 1650)
Stat5_SUPM2_Stat5 (2380, 2619)
Stat5_DEL_Stat5 (2479, 2659)

Software

FASTQC software (version 0.12.1) was used for quality control, Trimmomatic (version 0.39) was used to perform adapter trimming and quality-based filtering of reads. Murine reads were mapped against the GRCm38 primary assembly (Gencode M25), reads from human samples against the GRCh38 primary assembly (Gencode v26) using bwa-mem (version 0.7.15). Alignment QC was done with Qualimap (version 2.2.2a). Blacklisted regions were removed with bedtools subtract. Multimappers and reads with low mapping quality were removed using samtools (version 1.14). We used MACS3 to perform peak calling with default parameters. For each ChIP-seq file we used the corresponding Input file as the control. The resulting “narrowPeak” files were annotated using R (version 4.3.1) and the “GenomicFeatures” and “GenomicRanges” packages.

Flow Cytometry

Plots

Confirm that:

☐ The axis labels state the marker and fluorochrome used (e.g. CD4-FITC).

☐ The axis scales are clearly visible. Include numbers along axes only for bottom left plot of group (a 'group' is an analysis of identical markers).

☒ All plots are contour plots with outliers or pseudocolor plots.

☒ A numerical value for number of cells or percentage (with statistics) is provided.

Methodology

Sample preparation

Flow cytometry and Sorting
Single- cell suspensions were prepared from murine thymus, thymic lymphomas by straining through a sterile 40 µm filter. These suspensions or cultured cells were washed twice with PBS. Prior to surface marker staining, Fc receptors were blocked

	with purified anti-CD16/CD32 antibodies (clone 93; Thermo Fisher Scientific (eBioscience™)).<br><br>Intracellular stainings:<br>Ex vivo murine samples for pStat5 stainings were stimulated in complete RPMI using either 3000 U/mL human IL2 (Proleukin,SteriMax) or IL7 (50 ng/mL) for 20 min and compared with unstimulated samples. Surface panels for pSTAT5 stainings included the following antibodies: CD4 BV650, CD8 BV605, CD44 PE, CD25 Biotin, CD26 APC, lineage: (CD19 FITC, GR1 FITC, CD11B FITC) and for CDK6 stainings: CD4 BV650, CD8 FITC, CD44 PE, CD25 Biotin. Surface-stained cells were fixed with 2% parafolmaldehyde (Sigma-Aldrich) in PBS at 37°C for 10 min, then permeabilized with 90% ice-cold methanol in PBS containing 2% FCS and 0.2% Tween-20 for 30 min at 4°C. All subsequent washes were performed using PBS containing 2% FCS and 0.2% Tween-20.<br><br>Surfacemarker screens:<br>Single stains were done by using Fc-blocking antibodies (except for the analysis of CD16/CD32) and a subsequent incubation with the conjugated antibody of interest in the dark for 30 min at 4°C. Staining was performed separately for every cell line.<br><br>Cell cycle analysis:<br>Single cell suspensions from murine thymuses (40 μm filter) were Fc blocked (anti-CD16/CD32, Thermo Fisher) and surface stained for CD4 PE-Cy7, CD8a APC, CD25 PE, CD44 PerCP, Thy1.2 (BV510, 53-2.1), and a lineage panel (Gr-1, TER-119, CD19, CD11b; all APC-Cy7). Cells were fixed and permeabilized (BD Cytofix/Cytoperm, BD Biosciences) as adapted from Galvin et al. (2019), stained intracellularly for Ki-67 (FITC) , and counterstained for DNA with DAPI (2 μg/mL, Sigma-Aldrich D9542).
Instrument	Cytoflex S (Beckman Coulter), Cytoflex (Beckman Coulter), Cytoflex SRT (Beckman Coulter) or a BD FACSAria™ III Cell Sorter (BD Biosciences). Sorts for injections were performed with the Cytoflex SRT (Beckman Coulter) in purity mode and single- cell sorts even in single mode.
Software	Flow cytometry data were analyzed using CytExpert (Version 2.4.0.28, Beckman Coulter), CytExpert SRT (Version 1.1.0.10007, Beckman Coulter) or FlowJo (Version 10.8.-10.10.).
Cell population abundance	Cell population abundances were determined as percentages of total live, single cells after exclusion of doublets and debris, if not stated otherwise. Frequencies of major thymic populations were consistent across biological replicates. For clustering and UMAP analyses, equivalent numbers (1×10^4 lineage negative singlets pere mouse) were downsamples and concatenated to ensure equal representation across samples. For transplantation assays, 8×10^4 sorted cells from the indicated thymic subpopulations were injected per NSG recipient. Comparable proportions of the main thymic subsets were observed across genotypes and experimental replicates. For cell cycle analysis, the percentage of the DN cells was analyzed.
Gating strategy	Living single cells were identified using FSC/SSC parameters and doublet exclusion. Viability gating was validated in secondary experiments employing a viability dye (SYTOX™ Blue Dead Cell Stain (Fisher Scientific part of Thermo Fisher Scientific)) to ensure accuracy given the complexity of the staining panels. Lineage-negative thymocytes were defined as GR-1-,TER119-,CD19 and CD11b-. Downstream gating for T cell developmental populations used CD25,CD44 CD26, IL7R, TCRb and CD24, always starting from the CD4-CD8- gate. An exemplary gating strategy can be found in the supplemental Data. Other Combinations are outlined in the methods for every panel. Gating was based on FMO and single-stained controls and verified with biological replicates.<br>For cell cycle: Gating proceeded from live cells (FSC/SSC) to singlets (DAPI area versus width), lineage negative (APC-Cy7 dump), and Thy1.2 positive cells. Double negative thymocytes (CD4−CD8−) were resolved by CD44 and CD25 into DN1 (CD44+CD25−), DN2 (CD44+CD25+), DN3 (CD44−CD25+), and DN4 (CD44−CD25−). Within each subset, Ki-67 versus DAPI defined G0 (Ki-67−, 2N), G1 (Ki-67+, 2N), and S-G2-M (Ki-67+, >2N).

☒ Tick this box to confirm that a figure exemplifying the gating strategy is provided in the Supplementary Information.

Magnetic resonance imaging

Experimental design

Design type	*Indicate task or resting state; event-related or block design.*
Design specifications	*Specify the number of blocks, trials or experimental units per session and/or subject, and specify the length of each trial or block (if trials are blocked) and interval between trials.*
Behavioral performance measures	*State number and/or type of variables recorded (e.g. correct button press, response time) and what statistics were used to establish that the subjects were performing the task as expected (e.g. mean, range, and/or standard deviation across subjects).*

Acquisition

Imaging type(s)	*Specify: functional, structural, diffusion, perfusion.*
Field strength	*Specify in Tesla*
Sequence & imaging parameters	*Specify the pulse sequence type (gradient echo, spin echo, etc.), imaging type (EPI, spiral, etc.), field of view, matrix size, slice thickness, orientation and TE/TR/flip angle.*
Area of acquisition	*State whether a whole brain scan was used OR define the area of acquisition, describing how the region was determined.*

Diffusion MRI ☐ Used ☐ Not used

Preprocessing

Preprocessing software	*Provide detail on software version and revision number and on specific parameters (model/functions, brain extraction, segmentation, smoothing kernel size, etc.).*
Normalization	*If data were normalized/standardized, describe the approach(es): specify linear or non-linear and define image types used for transformation OR indicate that data were not normalized and explain rationale for lack of normalization.*
Normalization template	*Describe the template used for normalization/transformation, specifying subject space or group standardized space (e.g. original Talairach, MNI305, ICBM152) OR indicate that the data were not normalized.*
Noise and artifact removal	*Describe your procedure(s) for artifact and structured noise removal, specifying motion parameters, tissue signals and physiological signals (heart rate, respiration).*
Volume censoring	*Define your software and/or method and criteria for volume censoring, and state the extent of such censoring.*

Statistical modeling & inference

Model type and settings	*Specify type (mass univariate, multivariate, RSA, predictive, etc.) and describe essential details of the model at the first and second levels (e.g. fixed, random or mixed effects; drift or auto-correlation).*
Effect(s) tested	*Define precise effect in terms of the task or stimulus conditions instead of psychological concepts and indicate whether ANOVA or factorial designs were used.*

Specify type of analysis: ☐ Whole brain ☐ ROI-based ☐ Both

Statistic type for inference (See Eklund et al. 2016)	*Specify voxel-wise or cluster-wise and report all relevant parameters for cluster-wise methods.*
Correction	*Describe the type of correction and how it is obtained for multiple comparisons (e.g. FWE, FDR, permutation or Monte Carlo).*

Models & analysis

n/a	Involved in the study
☐	☐ Functional and/or effective connectivity
☐	☐ Graph analysis
☐	☐ Multivariate modeling or predictive analysis

Functional and/or effective connectivity	*Report the measures of dependence used and the model details (e.g. Pearson correlation, partial correlation, mutual information).*
Graph analysis	*Report the dependent variable and connectivity measure, specifying weighted graph or binarized graph, subject- or group-level, and the global and/or node summaries used (e.g. clustering coefficient, efficiency, etc.).*
Multivariate modeling and predictive analysis	*Specify independent variables, features extraction and dimension reduction, model, training and evaluation metrics.*
