# Supplementary material for: Ablation of CDK6 and STAT5B rewires T cell developmental dynamics and prevents ALK+ lymphoma formation
Source: Nat Commun. 2026 Sep 15;17:10853. doi: 10.1038/s41467-026-77466-4 (PMC13672290; doi:10.1038/s41467-026-77466-4)
Supplement: Supplementary file 3 [file 41467_2026_77466_MOESM3_ESM.pdf]

nature portfolio

Peer Review File

Ablation of CDK6 and STAT5B rewires T cell developmental dynamics and prevents ALK+ lymphoma formation

Corresponding Author: Dr Thorsten Klampfl

This file contains all reviewer reports in order by version, followed by all author rebuttals in order by version.

Version 0:

Reviewer comments:

Reviewer #1

(Remarks to the Author)
Kendler et al. present a comprehensive and mechanistically sophisticated study demonstrating that cooperative CDK6–STAT5B activity is essential for NPM–ALK–driven T-cell lymphomagenesis, acting at a defined DN2b/DN3a developmental checkpoint. Using a combination of genetic mouse models, single-cell transcriptomics, chromatin profiling, functional transplantation assays, and human ALCL validation, the authors establish that simultaneous loss of CDK6 and STAT5B completely abrogates tumor formation and induces synthetic lethality in human ALCL cells.

This is a well-executed study that provides novel insight into how oncogenic signaling intersects with thymocyte development to drive ALCL. The genetic evidence is compelling, the single-cell analyses are robust, and the translational relevance is clear.

Comments
1)The authors show that tumors do not develop in NPM–ALK transgenic mice (lacking CDK6 and STAT5b expression (NPM-ALKTg/+Cdk6-/-Stat5b-/- mice). They attribute this phenotype mainly to a developmental block at the DN2/DN3 thymocyte stage and report increased apoptosis at this stage compared with WT or NPM-ALKTg/+ mice. However, it remains unclear whether tumor prevention reflects the disruption of a specific NPM–ALK–driven transformation program or, more generally, a failure of DN thymocyte survival and/or proliferation. The authors are encouraged to comment on this point and clarify the underlying mechanism.

2) The age range at which the thymuses were analyzed is very broad (7–12.5 weeks). Some NPM-ALKTg/+ mice may have already developed thymoma and may even have died. This is supported by the survival curves (Fig. 1A) and by the literature. Performing the analyses at relatively advanced ages may compromise data interpretation, as some mice may already have developed pathology.
In addition, throughout the manuscript, the age of the mice is sometimes reported in days and sometimes in weeks; it would be preferable to use a consistent unit for clarity.

3)It would also be important to clarify at what age the scRNA-seq analysis of the thymus was performed.

4) In Figure 1C, the percentages of DP and DN thymocytes in NPM-ALKTg/+mice show a very wide range, whereas this is not observed in the NPM-ALKTg/+Cdk6-/-Stat5b-/- mice. Notably, DKO mice display DN percentages similar to those of WT mice. Could the authors comment on this point?

5) In Figure S1B-C. It would be important to assess NPM–ALK expression levels. Are the levels of NPM-ALK transduction and expression comparable? This analysis should also be performed using an NPM-ALK kinase dead.

6) In the experiments assessing the tumor-initiating capacity of CD26high DN2/3 thymocytes, it is not clear whether CD26 is functionally involved in this process or whether it is used merely as a phenotypic marker. Are CD26high thymocytes more proliferative than their CD26low counterparts?

7)Figure 2F: The data on WB cells and thymus weight are based on only three mice and show very high variability, which limits the strength of the conclusions. It would be helpful to clarify whether DN2/3 thymocytes co-express high levels of

CD26 along with elevated CDK6 and pSTAT5 upon IL-7 stimulation. Additionally, the pSTAT5 signal appears highly heterogeneous, which should be addressed or discussed in the context of the findings.

8) In figure 3H there is a decrease in DN1 thymocytes and an increase in DN2 cells in NPM-ALKTg/+Cdk6-/-Stat5b-/- compared to WT and NPM-ALKTg/+ mice. It would be helpful to provide a mechanistic explanation for the reduction in DN1 cells. What are the potential biological consequences of this DN1 decrease for thymocyte development? Clarifying this point would strengthen the interpretation of the data.

9) Figure 4F: To further strengthen the results, the authors should perform co-immunoprecipitation using an anti-STAT5 antibody in addition to the current co-IP with the anti-CDK6 antibody.

10) The degradation of CDK6 should be assessed by Western blot, and the effectiveness of CDK6 inhibition should also be confirmed by Western blot analysis, for example, by examining pRB1 or p21/p27 levels.

11) The STAT5 degrader used is not specific for STAT5B. How can the effects observed be distinguished from those resulting from the combined degradation of STAT5A and STAT5B? The authors should provide a comment or discussion on this point.

12) To further strengthen the synthetic lethality approach in a human context, it would be valuable to test the CDK6 degrader or inhibitor on STAT5B knockout cells, and conversely, assess the effects of STAT5B loss in the presence of CDK6 inhibition.

13) Figure 6AS: lanes in the western blot are missing, and the figure is not clear

14) Figure S6C please show the western blot also for SU-DHL1 and SUP-M2 cell lines.

Reviewer #2

(Remarks to the Author)
Dear Editor,
The manuscript in nice and elegant work to dissect the role CDK6 and STAT5B in NPM-ALK driven lymphoma. The Authors studied the effects of the genetic deletion of CDK6 and STAT5B in NPM-ALK transgenic mice. The absence of both CDK6 and STAT5B had a strong effect on the onset of such lymphoma, basically abrogating new lymphoma formation.
Large part of the work is focused on the development of T cells in the thymus, showing differences in cell maturation of T lymphocytes in the absence of both CDK6 and STAT5B, in particular at the DN2/3 stage. The Authors also studied the consequences of the genetic KO of CDK6 and STAT5B in human ALK+ ALCL lines, showing reduced cell growth and a synthetic lethality. In a limited series of ALK+ ALCL patients they found that a gene signatures potentially correlated with CDK6 and STAT5B could stratify the survival of patients.
The work is technically elegant, sound and well conducted, with the proper controls and experimental plan. The enthusiasm is somewhat diminished by considering that this work is incremental to what has been published by the same group in previous papers. Also, it is primarily focused on mouse T cell development, with little data from human lines or human samples. In it present form, the translatability of the findings to human ALK+ ALCL is unsure. The origin from a putative thymic precursor cell of ALK+ ALCL is still a controversial hypothesis in the field, thus these nice findings on mouse thymocytes might not readily translate into key biological findings in ALCL.

Major points:

- Gene signature and profiling should be repeated on human CDK6 and STAT5B KO cell lines or human samples with low CDK6 and STAT5B to determine the overlap seen with data obtained in mouse lines.
- The stratification of ALK+ALCL patients into groups with distinct overall survival based on the identified CDK6/STAT5B transcriptional programs (Fig. 6H) is weak due to the small number of cases analyzed. The Authors should determine directly the expression levels of STAT5B and CDK6 by IHC and use it to stratify with survival in ALCL.
- Is the signatures observed in DKO lines specific for ALK+ ALCL or simply a signature deriving from the ablation of these two genes in any T cell lymphoma line?

Reviewer #3

(Remarks to the Author)
This manuscript investigates the earliest oncogenic events in ALK^+ anaplastic large cell lymphoma using a genetic model of NPM-ALK mouse model combined with single-cell transcriptomics, chromatin analyses, transplantation, and human validation. CDK6 and STAT5B act as cooperative, non-redundant drivers of transformation specifically at the DN2/3

thymocyte stage, enforcing a tumor-specific developmental trajectory through shared transcriptional and chromatin programs. Dual loss of CDK6 and STAT5B completely blocks tumor initiation, induces apoptosis, and reveals a context-dependent synthetic lethal vulnerability with prognostic relevance in patients. The study is very impactful for the field.

Below, I am outlining a few suggestions that might help clarify aspects of the study:

DN2/3 thymocytes are the initiating population; can this be shown independently of scRNA-seq inference and transplantation? I suggest that the group performs limiting-dilution transplantation of purified DN subsets.

Hematopoietic-intrinsic effect: To fully exclude niche effects, the authors should ideally perform competitive thymic reconstitution assays.

Is the kinase activity of CDK6 necessary for the role of CDK6 and cooperativity with STAT5B activity or its transcriptional/scaffolding role? The authors can perform rescue studies using WT or kinase-dead CDK6. Additionally they could perform a comparison of the molecular signatures induced by CDK6 degrader and the CDK4/6 inhibitor.

Direct regulation of the chromatin effect: can the authors test the effect via ATAC-seq or CUT&RUN/CUT&Tag (e.g. H3K27me3, H3K4me3) in DN cells and cross-comparison to data in human systems?

What is the independent prognostic value of the 50-gene signature the authors identified? Can the authors test this using validation in different cohorts if available?

Version 1:

Reviewer comments:

Reviewer #1

(Remarks to the Author)
The authors have carefully revised the manuscript and provided detailed responses to my comments. The concerns raised during the previous review round have been satisfactorily addressed, and the additional data and clarifications have significantly strengthened the study.

In my opinion, the manuscript is now suitable for publication and meets the standards of Nature Communications. I therefore support its acceptance for publication.

Reviewer #2

(Remarks to the Author)
Dear Editor,
the Authors have satisfactorily revised the manuscript which is now clearly improved. In particular, they have strengthened the conclusions on human ALCL and the potential clinical value of the identified signature.

Open Access This Peer Review File is licensed under a Creative Commons Attribution 4.0 International License, which permits use, sharing, adaptation, distribution and reproduction in any medium or format, as long as you give appropriate credit to the original author(s) and the source, provide a link to the Creative Commons license, and indicate if changes were made.
In cases where reviewers are anonymous, credit should be given to 'Anonymous Referee' and the source.
The images or other third party material in this Peer Review File are included in the article's Creative Commons license, unless indicated otherwise in a credit line to the material. If material is not included in the article's Creative Commons license and your intended use is not permitted by statutory regulation or exceeds the permitted use, you will need to obtain permission directly from the copyright holder.
To view a copy of this license, visit https://creativecommons.org/licenses/by/4.0/

Reviewer's Comments:

Reviewer #1 (Remarks to the Author)

Kendler et al. present a comprehensive and mechanistically sophisticated study demonstrating that cooperative CDK6–STAT5B activity is essential for NPM–ALK–driven T-cell lymphomagenesis, acting at a defined DN2b/DN3a developmental checkpoint. Using a combination of genetic mouse models, single-cell transcriptomics, chromatin profiling, functional transplantation assays, and human ALCL validation, the authors establish that simultaneous loss of CDK6 and STAT5B completely abrogates tumor formation and induces synthetic lethality in human ALCL cells.

This is a well-executed study that provides novel insight into how oncogenic signaling intersects with thymocyte development to drive ALCL. The genetic evidence is compelling, the single-cell analyses are robust, and the translational relevance is clear.

Comments:

1)The authors show that tumors do not develop in NPM–ALK transgenic mice (lacking CDK6 and STAT5b expression (NPM-ALKTg/+Cdk6-/-Stat5b-/- mice). They attribute this phenotype mainly to a developmental block at the DN2/DN3 thymocyte stage and report increased apoptosis at this stage compared with WT or NPM-ALKTg/+ mice. However, it remains unclear whether tumor prevention reflects the disruption of a specific NPM–ALK–driven transformation program or, more generally, a failure of DN thymocyte survival and/or proliferation. The authors are encouraged to comment on this point and clarify the underlying mechanism.

We thank Reviewer #1 for overall positive response and also for important advice and suggestions how to further improve the revised version of our manuscript. We agree that disentangling a general survival/proliferation defect from disruption of a specific NPM-ALK transformation program is essential. Indeed, our data support a combined, rather than mutually exclusive, interpretation. The increased apoptosis observed at the DN2/DN3 stage **(Fig. 5C and Fig. S5G)** indicates that CDK6 and STAT5B act as critical survival mediators downstream of NPM-ALK at a developmental stage that is inherently apoptosis-sensitive (DN2/3 transition, or β-selection) in line with literature that displayed that Pre-T cell receptor localization and trafficking are independent of its signaling[1]. Their loss reduces the pool of cells available for transformation. However, several lines of evidence indicate that CDK6 and STAT5B are not merely permissive survival proteins but are also integral to the NPM-ALK oncogenic transcriptional program: (i) The developmental block at DN2/DN3 **(Fig. 3D)** is accompanied by a shift in differentiation-associated gene expression in the *NPM-ALK*$^{Tg/+}$*Cdk6*$^{-/-}$*Stat5b*$^{-/-}$ cells **(Fig. 3C, H, I)**, with a trend toward γδ rather than αβ T-cell commitment **(Fig. 3E-G)**. These findings argue for altered lineage trajectory rather than uniform cell loss. Moreover, a total failure of DN thymocyte survival and/or proliferation can be excluded as our flow cytometry and

scRNA-seq analysis present all 'non-transformed' cell clusters in the double knockout mice as well. (ii) By comparing NPM-ALK^{+} cells along the tumor trajectory (DN2/DN3 and transition cluster) with WT cells, we have now defined a 95-gene NPM-ALK transformation signature that is selectively upregulated in the *NPM-ALK*$^{Tg/+}$ but not the other genotypes **(Fig. R1A,** now **Fig. 4F** in the revised manuscript). (iii) Within this 95-gene program, 35 genes are specifically deregulated in the absence of CDK6 and STAT5B **(Fig. R1B,** now **Fig. 4G)**. Thus, our data demonstrate that CDK6-STAT5B both shape and not just permit an oncogenic gene expression program. (iv) The relevance of this "re-programming" may extend to human disease: in an ALK^{+} ALCL biopsy analyzed by scRNA-seq, we found significant enrichment of the NPM-ALK transformation signature in ALK^{+} tumor cells compared to ALK^{-} microenvironmental cells **(Fig. R1C, D,** now **Fig. 4H, I)**. In line with above argumentation, the CDK6/STAT5-dependent 35-gene subset was specifically enriched in the cluster harboring the highest proportion of ALK^{+} cells **(Fig. R1E,** now **Fig. 4J)**. Together, these findings indicate that tumor prevention in *NPM-ALK*$^{Tg/+}$*Cdk6*$^{-/-}$*Stat5b*$^{-/-}$ mice cannot only be attributed to reduced thymocyte survival but it could reflect a failure to establish and sustain the specific NPM-ALK-dependent transformation program via transcriptional re-wiring.

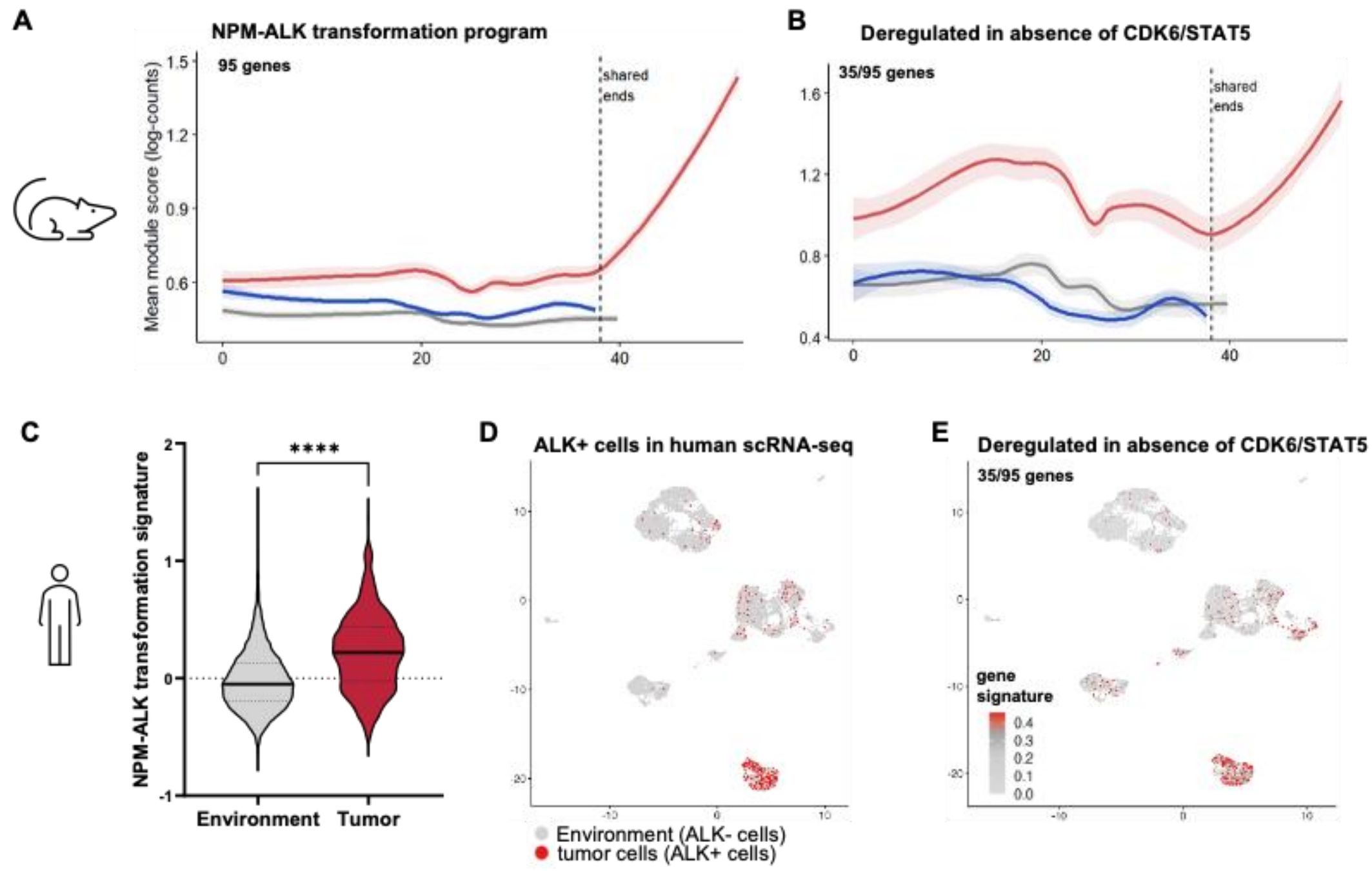

Figure R1: The NPM-ALK transformation program is partially dependent on CDK6 and STAT5B and is conserved in human ALK^{+}ALCL.

*(A) Mean score (log-counts) of the 95-gene NPM-ALK transformation signature along the pseudotime trajectory of thymocyte development in mouse scRNA-seq data. The dashed line ("shared ends") marks the last pseudotime point at which all three genotypes contribute cells. Beyond this point, only NPM-ALK$^{Tg/+}$ cells progress along the tumor trajectory. (B) Mean module score of the 35-gene subset of the NPM-ALK transformation signature that is specifically deregulated in the absence of CDK6 and STAT5B. (C) Violin plot of the 95-gene NPM-ALK transformation signature score in tumor cells (ALK^{+}, red) versus CD45+ environmental cells (ALK^{-}, grey) from a human CD45+ sorted ALK^{+}ALCL biopsy analyzed by scRNA-seq; n = 1 ALK^{+}ALCL biopsy, 7554 cells after QC. Horizontal lines indicate median and quartiles (**** P < 0.0001, two-sided Mann-Whitney test). (D) UMAP of single cells from the human ALK^{+} ALCL biopsy. Cells are colored by ALK status: ALK^{+} tumor cells (red) and ALK^{-}*

environmental cells (grey). (E) UMAP, colored by the module score of the 35-gene CDK6/STAT5-dependent subset of the NPM-ALK transformation signature.

Overall, we thank the reviewer for this important comment, and we revised the text of the manuscript better to highlight this argumentation as amended in the newly revised version. We also added it as part of a new main **Fig. 4.**

2) The age range at which the thymuses were analyzed is very broad (7–12.5 weeks). Some NPM-ALKTg/+ mice may have already developed thymoma and may even have died. This is supported by the survival curves (Fig. 1A) and by the literature. Performing the analyses at relatively advanced ages may compromise data interpretation, as some mice may already have developed pathology. In addition, throughout the manuscript, the age of the mice is sometimes reported in days and sometimes in weeks; it would be preferable to use a consistent unit for clarity.

We thank the reviewer for this thoughtful comment. The selected age range was chosen intentionally. On the one hand we had to account for the heterogeneous disease onset in *NPM-ALK*$^{Tg/+}$ mice, as a subset of animals shows early signs of transformation at this stage, whereas others develop disease considerably later. On the other hand our analysis took in parallel also care of the 3R principle, where we had to limit the number of mice for analysis achieving a compromise in analyzed aged cohort groups. This time window of analysis was therefore chosen to capture early transforming events across animals at different stages rather than restricting the analysis to a single chronological time point. We want to point out that our analysis revealed that none of the mice exhibited advanced disease at this stage.

As shown in **Fig. R2A** and **R2B**, the age distribution of the analyzed cohorts does not differ significantly between genotypes. Importantly, when testing whether age influences the relative size of the DN fraction within this range, we observed no age-dependent trend in *NPM-ALK*$^{Tg/+}$ mice.

This difference further supports the interpretation that DN expansion represents an early event during transformation rather than a secondary consequence of disease progression, and that the selected age range does not significantly confound the analysis of the NPM-ALK genotype.

For the display of DN stages, we selected only mice with a percentage of DN below 20% within the major thymus subsets and with a thymus/body weight ratio below 1%. Samples above this threshold are marked with their respective genotype and the annotation "transforming".

We also agree with the reviewer regarding consistency in age reporting and have revised the manuscript to uniformly report mouse age in weeks throughout.

For Reviewers only:

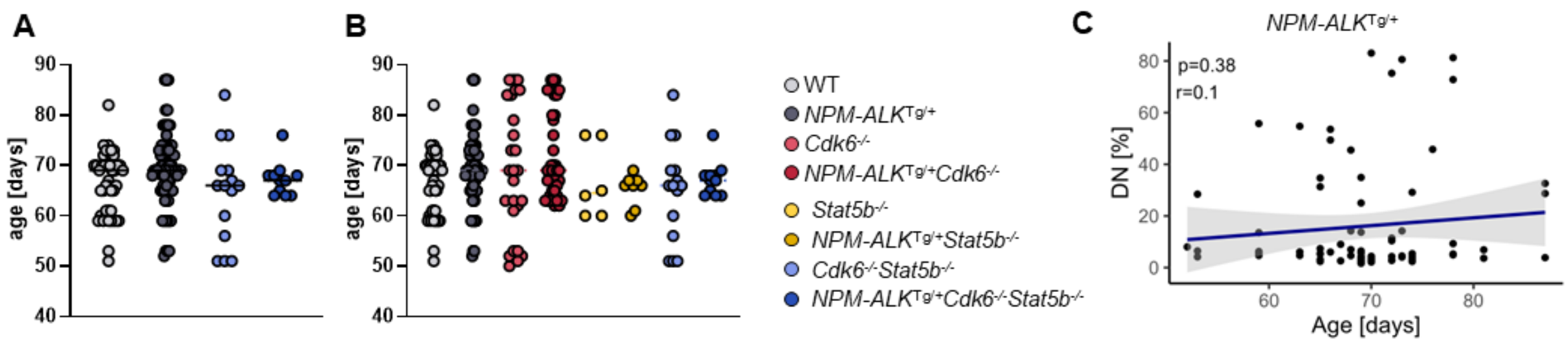

Figure R2: Age distribution of analyzed cohorts and absence of an age effect on DN fraction.

(A) Age at analysis of mice used for subset profiling. Statistical significance was assessed by one-way ANOVA followed by Tukey's multiple comparisons. Line indicates the median. (B) Age at analysis for the full panel of genotypes. Statistical significance was assessed by one-way ANOVA followed by Tukey's multiple comparisons test. Lines indicate the median. (C) Correlation between age at analysis and the percentage of DN thymocytes in NPM-ALK$^{Tg/+}$ mice within the analyzed age range. Each dot represents one animal; the blue line shows the Pearson correlation.

3)It would also be important to clarify at what age the scRNA-seq analysis of the thymus was performed.

We thank the reviewer for this suggestion. The scRNA-seq analysis was performed on FACS-purified thymic DN cells isolated from mice aged 10 weeks, but it was already highlighted in the Figure legend **1D**:

"**(D)** Uniform Manifold Approximation and Projection (UMAP) plot of scRNA-seq data from FACS sorted thymic DN T cells from WT, *NPM-ALK*$^{Tg/+}$ and *NPM-ALK*$^{Tg/+}$*Cdk6*$^{-/-}$*Stat5b*$^{-/-}$ mice (20620 cells from *n* = 3 mice: all female, age 69-72 days)"

and in the Methods section:

"FACS-sorted thymic DN T cells from WT, *NPM-ALK*$^{Tg/+}$ and *NPM-ALK*$^{Tg/+}$Cdk6$^{-/-}$Stat5b$^{-/-}$ mice (n = 3 female mice, aged 69-72 days) were subjected to library preparation for scRNA-seq using technology from 10x Genomics according to the manufacturer's instructions.", where we think that it suffices for detailed description.

4) In Figure 1C, the percentages of DP and DN thymocytes in NPM-ALKTg/+mice show a very wide range, whereas this is not observed in the NPM-ALKTg/+Cdk6-/-Stat5b-/- mice. Notably, DKO mice display DN percentages similar to those of WT mice. Could the authors comment on this point?

We thank the reviewer for this observation, and we discussed it within our consortium. The wide range of DN and DP frequencies observed in *NPM-ALK*$^{Tg/+}$ mice reflect the asynchronous nature of NPM-ALK driven transformation (similarly as described in the answer to question 2 above), with individual animals at the same age displaying varying degrees of early disease progression. Accordingly, some mice already show pronounced DN expansion, whereas others remain closer to a WT-like thymic composition for longer.

In the absence of CDK6 and STAT5B, the thymic cells do not undergo transformation, resulting in a more homogeneous thymocyte distribution that closely resembles WT mice. The absence of variability in the *NPM-ALK*$^{Tg/+}$*Cdk6*$^{-/-}$*Stat5b*$^{-/-}$ cohort therefore supports the conclusion that

DN expansion is specifically associated with NPM-ALK driven transformation and depends on CDK6 and STAT5B.

5) In Figure S1B-C. It would be important to assess NPM–ALK expression levels. Are the levels of NPM-ALK transduction and expression comparable? This analysis should also be performed using an NPM-ALK kinase dead.

We thank the reviewer for this important point. NPM-ALK expression levels were assessed by flow cytometry using the GFP reporter linked to the NPM-ALK construct. While transduction efficiency was slightly lower in the *NPM-ALK*$^{Tg/+}$*Stat5b*$^{-/-}$ condition, comparison of GFP MFI among GFP-positive cells revealed no statistically significant differences between genotypes. The most pronounced comparison (*NPM-ALK*$^{Tg/+}$*Stat5b*$^{-/-}$ vs *NPM-ALK*$^{Tg/+}$*Cdk6*$^{-/-}$*Stat5b*$^{-/-}$) did not reach significance (p=0.13 by One way ANOVA), indicating comparable NPM-ALK expression levels across conditions (**Fig. R3,** now **Fig. S1B-C** in the revised manuscript). Importantly, no single genotype analyzed in the different mouse cohorts showed preferential outgrowth associated with higher NPM-ALK expression.

With regard to the use of a kinase-dead NPM-ALK construct, these experiments were performed in a cytokine and growth factor free methylcellulose system, as described in the Methods section of the originally submitted manuscript. This experimental setup specifically selects for kinase-active NPM-ALK-dependent survival and expansion. Under these conditions, kinase-dead NPM-ALK does not support outgrowth and therefore cannot be meaningfully assessed in this assay.

We identified an incorrect annotation in **Fig. S1B** depicting the colony formation workflow, where NPM-ALK was inadvertently labeled prior to transduction, which we apologize for and correct now in the revised version of this manuscript. NPM-ALK expression is introduced only after the transduction step and this mistake by us could have let to misconception which we hope to now have better outlined. We have corrected the Figure annotation accordingly and clarified this point also in the Figure legend.

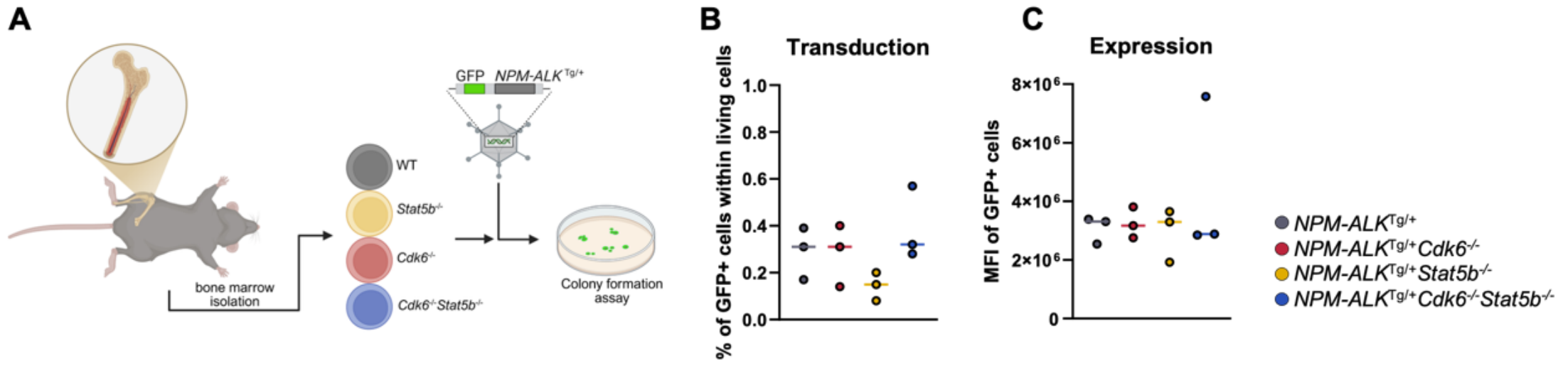

Figure R3: Comparable NPM-ALK transduction and expression across genotypes.

(A) Schematic of the experimental workflow. (B) Transduction efficiency, expressed as the percentage of GFP+ cells within the living cell population (n=3 per genotype). Statistical significance was assessed by one-way ANOVA followed by Tukey's multiple comparisons test. Lines indicate the median. (C) NPM-ALK expression levels, shown as the mean fluorescence intensity (MFI) of GFP within the GFP+ cell population (n =3 per genotype). Statistical significance was assessed by one-way ANOVA followed by Tukey's multiple comparisons test. Lines indicate the median.

6) In the experiments assessing the tumor-initiating capacity of CD26high DN2/3 thymocytes, it is not clear whether CD26 is functionally involved in this process or whether it is used merely as a phenotypic marker. Are CD26high thymocytes more proliferative than their CD26low counterparts?

In our study, CD26 was primarily used as a phenotypic marker to enable the *ex vivo* identification of the tumor cluster population that emerged from our scRNA-seq analysis, where CD26 was among the top differentially expressed markers of the tumor cluster (**Fig. 2A**). The experiments assessing tumor-initiating capacity were therefore designed to test whether the $CD26^{high}$ population identified transcriptionally also exhibits enhanced tumorigenic potential *in vivo*, rather than to directly address a functional role of CD26 itself.

We therefore think that CD26 expression could serve as surrogate marker of an early transforming state, rather than as a demonstrated driver/biomarker of transformation that we do not highlight as such. We have tried to clarify this point now better in the revised manuscript to avoid overinterpretation of CD26 as a functional effector.

7)Figure 2F: The data on WB cells and thymus weight are based on only three mice and show very high variability, which limits the strength of the conclusions.

It would be helpful to clarify whether DN2/3 thymocytes co-express high levels of CD26 along with elevated CDK6 and pSTAT5 upon IL-7 stimulation. ,the pSTAT5 signal appears highly heterogeneous, which should be addressed or discussed in the context of the findings.

We thank the reviewer for raising these important points, but as argued above we need to work according to the 3R principle and our analysis of mice is sometimes limited by numbers. Regarding the white blood cell counts and thymus weight shown in **Fig. 2F**, these data are based on 4 mice in the CD26-high cohort. One *NPM-ALK*$^{Tg/+}$ animal is indicated by an "x" in the plot because its WB cell count exceeded the upper detection limit of the hematology analyzer and could therefore not be quantified precisely. We have amended the Figure legend accordingly. While variability is indeed high in $CD26^{high}$ animals, it is important to note that all $CD26^{low}$ controls consistently showed low WB cell counts with minimal variation. Moreover, all

CD26^{high} animals developed disease with variable phenotypes **(Fig. 2G)**, whereas CD26^{low} mice did not show any significant signs of the disease.

Regarding co-expression of CD26, CDK6, and pySTAT5 under IL7 stimulation, simultaneous assessment of all three markers in a single staining was unfortunately not technically feasible due to fluorochrome and antibody compatibility limitations. However, CDK6 and pSTAT5 were analyzed in the same animals, and we would not expect the short-term IL7 stimulation (20 min) to alter total CDK6 levels, as such short stimulations typically affect only phosphorylation states rather than total protein abundance. To better characterize the heterogeneous pSTAT5 levels, we additionally analyzed pSTAT5 in early transforming thymocytes **(Fig. R4A** now added to **Fig. 2D)**. Importantly, we observe that increasing thymus-to-body-weight ratios are accompanied by elevated CD26 and pSTAT5 signals **(Fig. R4A** now added to **Fig. 2D)**, supporting a potential link between these features during early transformation. In addition, analysis of independent ALCL patient datasets revealed a significant correlation between DPP4 (CD26) and CDK6 and STAT5B expression in two patient datasets **(Fig. R4B** now added as **Fig. S2L)**, providing further clinical relevance.

Importantly, scRNA-seq analysis provides direct evidence for coordinated upregulation of *Cdk6, Dpp4* (CD26), and *Stat5b* during early transformation. Along the tumor trajectory, these genes are upregulated at the DN2–DN3 transition **(Fig. R4C, D** now added as **Fig. S2J, K)**. Moreover, within DN2/DN3 thymocytes, cells expressing higher levels of DPP4 also show elevated CDK6 and STAT5B expression, indicating that these features co-segregate within the same cell population rather than representing independent events **(Fig. R4C, D** now added as **Fig. S2J, K)**.

With respect to the heterogeneous pSTAT5 signal, we agree that heterogeneity is evident, particularly in NPM-ALK samples. We believe this reflects biological heterogeneity during early transformation rather than technical variability. Specifically, a subset of DN2 cells re-expresses CD44, a phenomenon that becomes more pronounced during early transformation and is documented in **Fig. S2B** and **Fig. S3F**. As CD44 upregulation marks a distinct subpopulation, this likely contributes to the observed variability in pSTAT5 signaling.

The manuscript text has been amended for these findings.

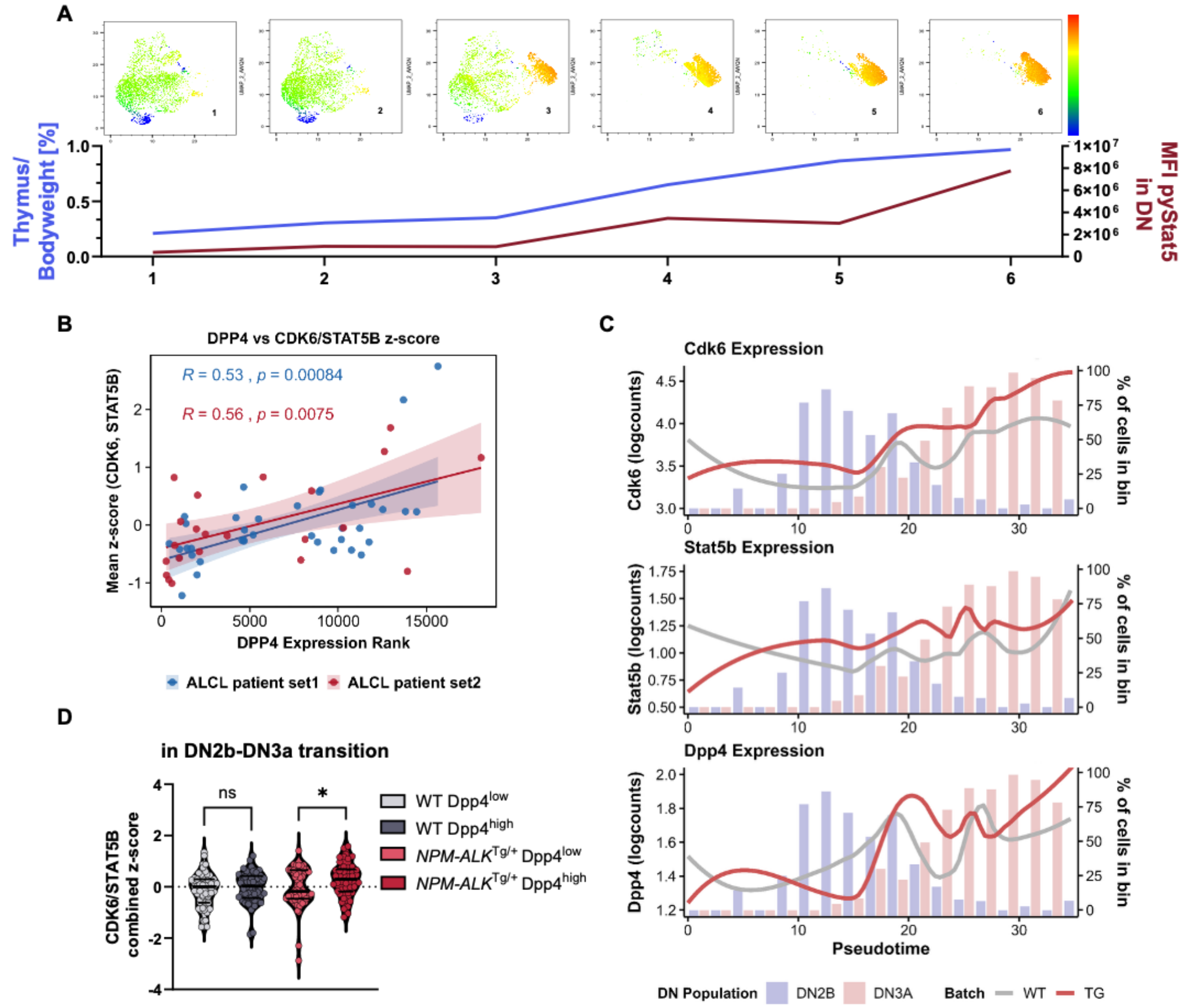

Figure R4: Coordinated upregulation of DPP4 (CD26), CDK6, and STAT5B during early thymic transformation.

(A) Six NPM-ALK$^{Tg/+}$ samples from (B, C), ordered by thymus-weight/body-weight (age sample 1-5 = 87-88 days, sample 6 = 110 days). UMAP plots show CD26 levels; thymus-weight/body-weight and pYSTAT5 MFI in DN thymocytes (red, right axis) is plotted below. (B) Spearman rank correlation of DPP4 expression with CDK6 (left) and STAT5B (right). (C) Gene expression of Cdk6, Stat5b and Dpp4 along pseudotime in WT (grey) and NPM-ALK$^{Tg/+}$ (red) thymocytes derived from scRNA seq. In the background, the transition of DN2b to DN3a cells is displayed. (D) Violin plots comparing CDK6/STAT5B z-score in Dpp4^{high} vs Dpp4^{low} expressing cells (median split of Dpp4). Lines show median and quartile expression. Statistical significance was calculated within each genotype by ordinary one-way ANOVA with Sidak's multiple comparisons test ($p < 0.05$).*

8) In figure 3H there is a decrease in DN1 thymocytes and an increase in DN2 cells in NPM-ALKTg/+Cdk6-/-Stat5b-/- compared to WT and NPM-ALKTg/+ mice. It would be helpful to provide a mechanistic explanation for the reduction in DN1 cells. What are the potential biological consequences of this DN1 decrease for thymocyte development? Clarifying this point would strengthen the interpretation of the data.

We thank the reviewer to point that out and we follow the advice to possibly explain it better mechanistically as follows: The reduction in DN1 thymocytes in DKO mice is likely driven by a combination of impaired survival and premature differentiation. IL-7 signaling is essential for thymocyte survival and development, particularly at early DN stages, where it promotes

lymphocyte expansion as well as proliferation and prevents apoptosis via STAT5 activation[2–5]. It is well established that loss of STAT5 signaling leads to reduced T cell survival, highlighting its critical role downstream of IL7[6,7]. In line with this, we observed reduced IL7R expression in *NPM-ALK*$^{Tg/+}$*Cdk6*$^{-/-}$*Stat5b*$^{-/-}$ DN1 cells likely resulting in diminished survival signals and contributing to the depletion of the DN1 compartment **(Fig. S5C)**. However, as stated before our data clearly show that besides these shifts in these two populations, DKO mice have all 'non-transformed' cell populations.

To further dissect the mechanism and its developmental consequences, we performed a pseudotime trajectory analysis across the DN1-to-DN2 transition. At the signature level, *NPM-ALK*$^{Tg/+}$*Cdk6*$^{-/-}$*Stat5b*$^{-/-}$ cells showed a marked reduction of the WT DN1 gene signature and a concomitant enrichment of the WT DN2a signature compared with WT and *NPM-ALK*$^{Tg/+}$ counterparts **(Fig. R5A)**, confirming the flow-cytometric phenotype of **Fig. 3D** at the transcriptome level.

Beyond impaired survival seen at the DN2 stage, the reduction of the DN1 compartment in NPM-ALK$^{Tg/+}$Cdk6$^{-/-}$Stat5b$^{-/-}$ mice therefore reflects a premature exit from the multipotent progenitor state and accelerated entry into the T-lineage commitment program[8]. Genes characteristic of multipotent and early thymic progenitor genes (*Bcl11a, Kit, Spi1, Tyrobp, Tcrg-c1* and *Mef2c*) were downregulated earlier along pseudotime in *NPM-ALK*$^{Tg/+}$*Cdk6*$^{-/-}$*Stat5b*$^{-/-}$ cells than in WT or *NPM-ALK*$^{Tg/+}$ cells **(Fig. R5B,** upper panel**)**[8]. In parallel, key transcription factors of the T-lineage commitment program (*Bcl11b, Gata3, Notch1, Runx1,* and *Ets1*) were upregulated prematurely in *NPM-ALK*$^{Tg/+}$*Cdk6*$^{-/-}$*Stat5b*$^{-/-}$ cells **(Fig. R5B,** lower panel**)**[8,9]. Notably, β-selection-associated genes including *Rag1* and *Ptcra* were also induced earlier in *NPM-ALK*$^{Tg/+}$*Cdk6*$^{-/-}$*Stat5b*$^{-/-}$ **(Fig. R5B,** right**)**, indicating that the shift affecting the DN1-DN2 transition propagates through subsequent developmental checkpoints, consistent with STAT5-dependent control of TCR locus accessibility[3,10]. Because IL7-dependent signaling is also required for proliferation, differentiation, and β-selection processes in DN thymocytes, perturbations in this pathway likely cooperate with the transcriptional shift to alter developmental timing[3].

These transcriptional shifts have several biological consequences for thymocyte development. The DN1 compartment is highly heterogeneous and contains the earliest thymic progenitors (ETPs), which retain residual multi-lineage potential and serve as the proliferative reservoir for all downstream thymocyte subsets[8]. A shortened DN1 phase therefore reduces the time available for progenitor expansion and lineage diversification, which may influence the timing of subsequent developmental stages. Moreover, premature induction of commitment factors such as Bcl11b is known to irreversibly close alternative lineage options and restrict developmental plasticity[9,11]. Together, these findings indicate that combined loss of CDK6 and

STAT5B disrupts the coordination between progenitor maintenance and T-lineage commitment through both reduced IL7/STAT5-mediated survival signals and premature activation of the commitment program. This likely contributes to the altered developmental progression and the bottleneck at the DN2 stage that underlies the altered DN1:DN2 ratio observed in **Fig. 3D**.

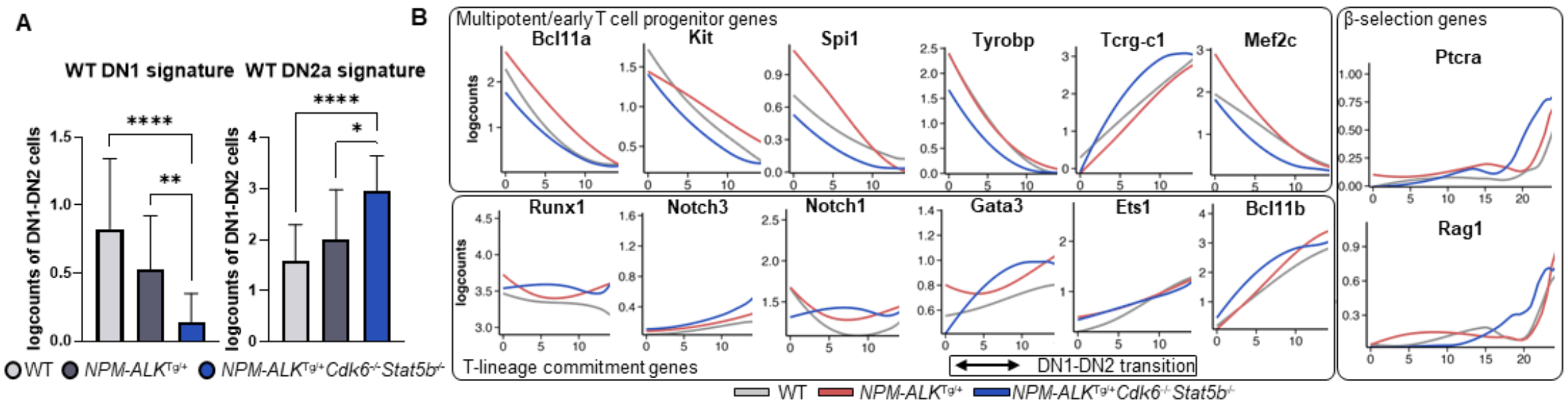

Figure R5: Combined loss of CDK6 and STAT5B accelerates the DN1 to DN2 transition at the transcriptional level.

*(A) Module scores of the WT DN1 signature (left) and the WT DN2a signature (right) in DN1 and DN2 thymocytes. Bars represent mean and error bars standard deviation. Statistical significance was assessed by Kruskal-Wallis test with Dunn's multiple comparisons test (*p < 0.05, **p < 0.01, ****p < 0.0001). (B) Pseudotime trajectory analysis of single cells spanning the DN1 to DN2 transition. Upper panel shows multipotent and early T cell progenitor genes while the lower panel shows T lineage commitment genes. In the right panel, β-selection associated genes are displayed. The double headed arrow marks the DN1 to DN2 transition along the pseudotime.*

<u>9) Figure 4F: To further strengthen the results, the authors should perform co-immunoprecipitation using an anti-STAT5 antibody in addition to the current co-IP with the anti-CDK6 antibody.</u>

We thank the reviewer for this valuable suggestion. We have performed the requested experiment and included the results in the revised manuscript as **Fig. S5I**. Specifically, we conducted co-immunoprecipitation using an anti-STAT5B antibody and were able to confirm the interaction, consistent with our initial findings using the anti-CDK6 antibody.

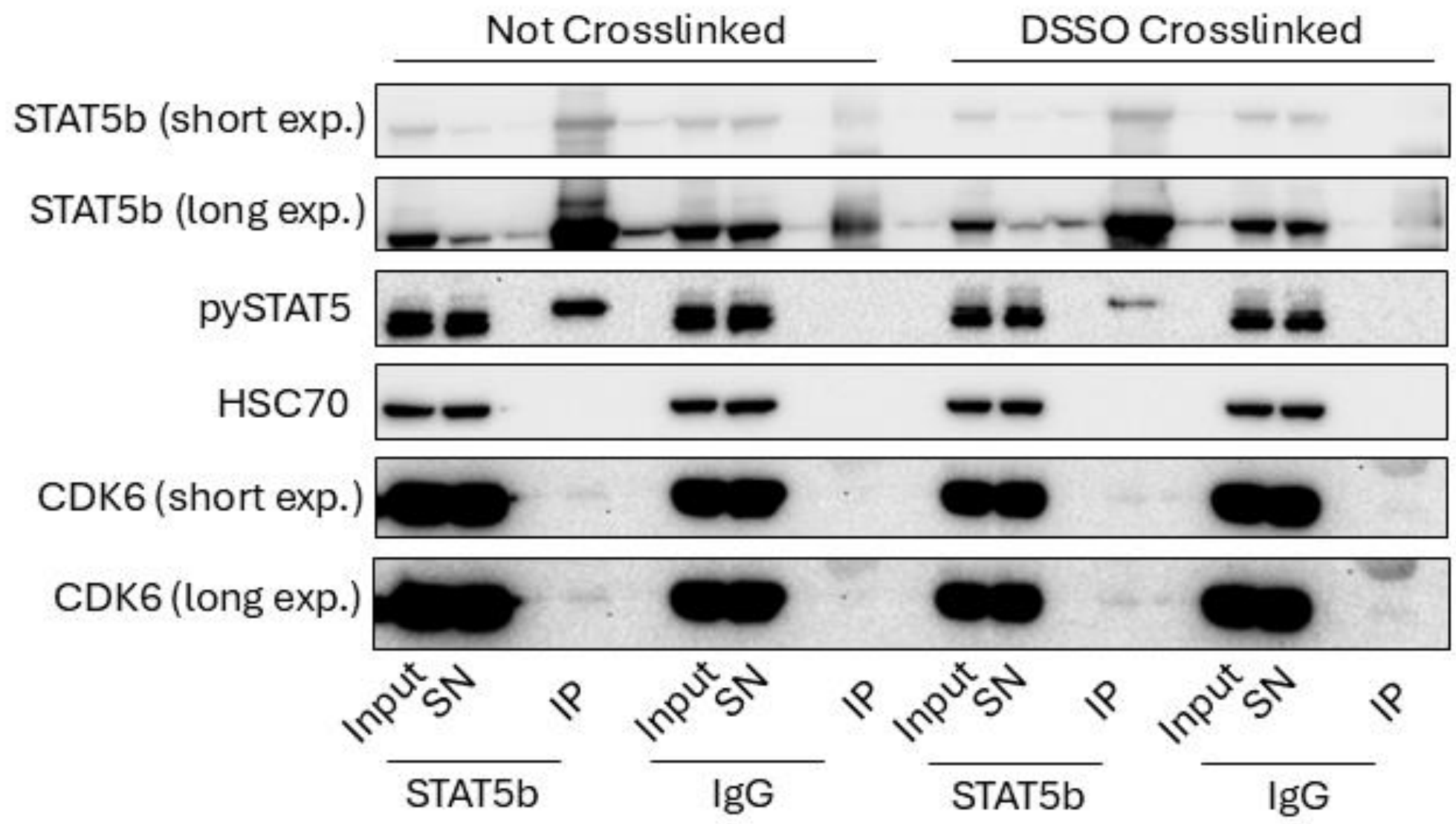

Figure R6: Western blot of STAT5B IP in a human ALCL cell line (SU-DHL 1) +/- crosslinker DSSO. IgG was the negative control and HSC70 the loading control.

The text of the manuscript was amended in line 255.

10) The degradation of CDK6 should be assessed by Western blot, and the effectiveness of CDK6 inhibition should also be confirmed by Western blot analysis, for example, by examining pRB1 or p21/p27 levels.

We again thank the reviewer for careful reading and advice, where we now have also performed these control experiments and added the results as **Fig. S6D, E** to the revised manuscript. The degradation of CDK6 upon treatment with BSJ-03-123 was confirmed as well as the inhibition of CDK6 by Palbociclib, as shown by reduced pRB1.

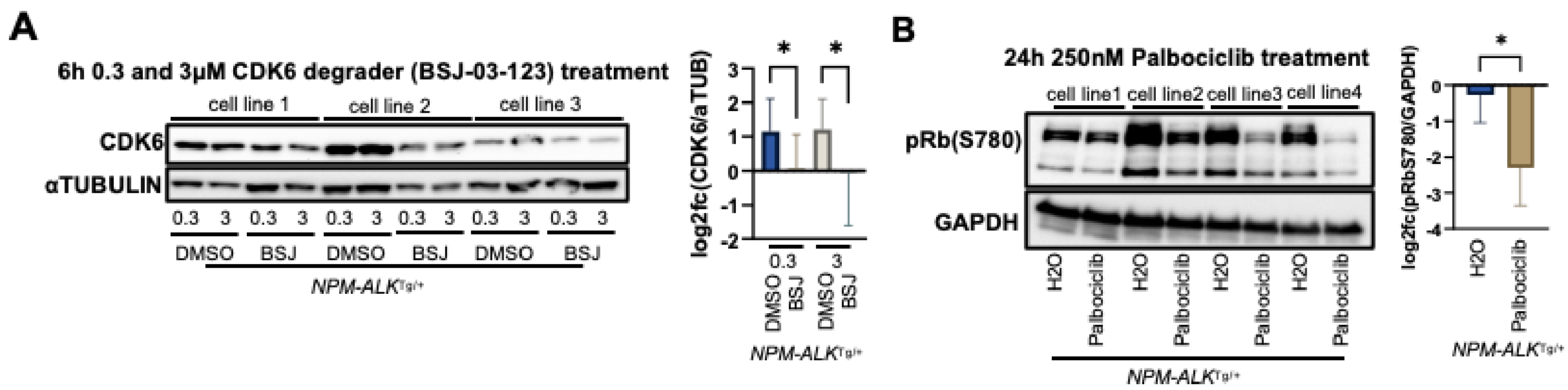

Figure R7: Validation of CDK6 degradation by BSJ-03-123 and CDK6 kinase inhibition by Palbociclib.

(A) Representative Western blot analysis of CDK6 protein levels in cell lines treated for 6 h with either DMSO or the CDK6 degrader BSJ-03-123 at 0.3 µM or 3 µM. αTUBULIN served as loading control. Data are presented as mean with standard deviation. Statistical analysis was performed by ordinary two-way ANOVA with Sidak's multiple comparisons test ($p < 0.05$). (E) Representative Western blot analysis of phosphorylated Rb at Ser780 (pRb(S780)) in murine cell lines. GAPDH served as loading control. Data are presented as mean with standard deviation. Statistical analysis was performed by two tailed paired t test (* $p < 0.05$).*

11) The STAT5 degrader used is not specific for STAT5B. How can the effects observed be distinguished from those resulting from the combined degradation of STAT5A and STAT5B? The authors should provide a comment or discussion on this point.

We agree that the STAT5 degrader used in this study targets both STAT5A and STAT5B and therefore does not allow discrimination between gene product specific effects at the level of protein degradation. However, our mouse models and CRISPR screen highlight a more prominent role for STAT5B in this disease system, which we try to better discuss in the revised version of this manuscript.

Importantly, for ALK$^+$ALCL specifically, STAT5A is often epigenetically silenced through promoter DNA methylation and it can act as a reciprocal tumor suppressor in this disease as originally reported[12], meaning that STAT5B is the functionally available STAT5 gene product in NPM-ALK driven lymphomagenesis. We acknowledge that these data do not formally exclude potential contributions of residual STAT5A in other cellular contexts. However, from a translational perspective, dual STAT5A/STAT5B targeting may in fact be advantageous in settings where both gene products are expressed, as previously demonstrated in ALK$^+$ALCL where combined loss of STAT5A and STAT5B is more deleterious than loss of either isoform alone[13].

12) To further strengthen the synthetic lethality approach in a human context, it would be valuable to test the CDK6 degrader or inhibitor on STAT5B knockout cells, and conversely, assess the effects of STAT5B loss in the presence of CDK6 inhibition.

To answer the reviewer question in more depth, we first determined the degradation efficacy of the CDK6 degrader, as this can be counteracted by CDKN binding to CDK6[14]. SU-DHL-1 cells show virtually no significant degradation of CDK6, which is accompanied by high CDKN levels that likely prevent degradation **(Fig. R8A)**. The scatter plot of CDK6 IP-MS LFQ intensities confirms strong association of CDK6 with CDKN1A, CDKN1B and CDKN2A in both untreated and Palbociclib-treated conditions, consistent with CDKN shielding from PROTAC-induced degradation. In contrast, DEL cells showed a better degradation efficacy **(Fig. R8B)**, which is in line with the absence of CDKN interactors found in the CDK6 IP-MS and no p16 expression is seen in the DEL cell lines.

To test the essentiality of the CDK6 kinase function on apoptosis onset, which is described as one critical mechanism in the DKO cells **(Fig. 5C** and **Fig. S5G, H)**, we treated wildtype (ALK) and STAT5B knockout DEL clones (BKO) with Palbociclib or BSJ-03-123 and analyzed apoptosis onset by flow cytometry. CDK6 degradation showed a significant increase in

apoptosis in STAT5B knockout cells compared to wildtype cells, which was not mimicked by Palbociclib, highlighting a largely kinase-independent role of CDK6 in combination with STAT5B loss to induce apoptosis.

Notably, degradation in DEL cells was less efficient in the BKO background than in WT: BSJ-03-123 reached statistical significance in WT ($P < 0.05$, two-way ANOVA with Sidak's multiple comparisons test) but only trended downward in BKO ($P = 0.16$, ns; **Fig. R8C**). This incomplete and genotype-dependent degradation efficacy is likely a phenomenon specific to the human cell line system and it was not observed in our murine setting. Despite the weaker CDK6 reduction in BKO cells, BSJ-03-123 still produced a highly significant apoptotic response selectively in this genotype ($P = 0.0004$; **Fig. R8D**), whereas WT cells did not respond, suggesting that even partial CDK6 degradation is sufficient to drive the phenotype and that a more complete CDK6 reduction would likely amplify it further. Since pharmacological CDK6 depletion in the human system is incomplete and variable across genotypes, the genetic CRISPR knockout strategy for CDK6 and STAT5B presented in the paper is better suitable to address the question mechanistically **(Fig. 7A-E)**.

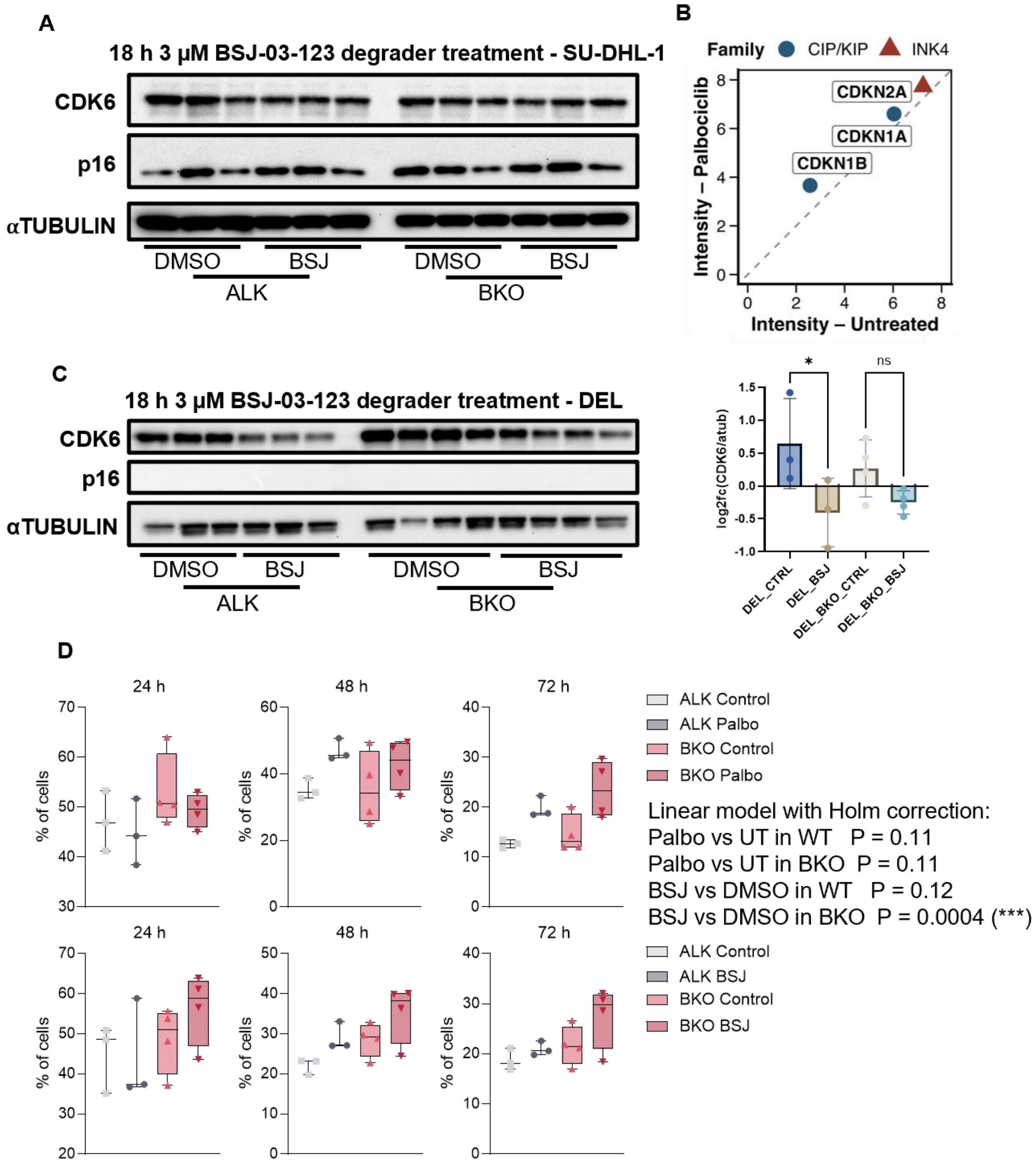

Figure R8: Pharmacological CDK6 inhibition and degradation phenocopy CDK6 loss in a STAT5B dependent manner only partially, in line with incomplete protein reduction.

(A) Western blot of CDK6, p16^{INK4A} and α-tubulin (loading control) in SU-DHL-1 after 18 h of treatment with 3 µM of the BSJ-03-123 CDK6 PROTAC degrader or DMSO vehicle control, in ALK (parental) and BKO (STAT5B knockout) backgrounds. (B) Scatter plot of CDK6 IP-MS LFQ intensities for CDKN1A, CDKN1B (CIP/KIP family) and CDKN2A (INK4 family) in untreated versus palbociclib-treated SU-DHL-1 cells. The dashed diagonal indicates equal intensity. (C) Validation of CDK6 protein levels after BSJ-03-123 treatment in DEL cells. Left: representative Western blot for CDK6 and the loading control α-tubulin in ALK and BKO DEL cells treated for 18 h with DMSO or 3 µM BSJ-03-123. Right: quantification expressed as log2 fold change of CDK6 over α-tubulin. Each dot represents one independently cultured clone replicate; bars show mean with standard deviation. Statistical analysis was performed by ordinary two-way ANOVA comparing BSJ treated samples to their control with Sidak's multiple comparisons test ($P < 0.05$; ns, not significant). (D) Apoptosis assessment by Annexin V/7AAD staining in WT (ALK) and STAT5B knockout (BKO) DEL ALCL cells at 24, 48 and 72 hours. Top row: untreated control versus 200 nM palbociclib (Palbo). Bottom row: vehicle control (DMSO) versus 3 µM BSJ-03-123 (BSJ). Box plots show the apoptotic cell fraction with min to max range and all individual data points; $n = 3$ WT and $n = 4$ BKO replicates per condition. P values are from a linear model fitted across all three timepoints, with replicate as a covariate, and Holm corrected across the four planned contrasts; the control condition was H_2O for the palbociclib arm and DMSO for the BSJ arm (*** $P < 0.001$).*

13) Figure 6AS: lanes in the western blot are missing, and the figure is not clear

We thank the reviewer for this comment and apologize for the lack of clarity in the original **Fig. S6A (**now **Fig. S7A)**. We would like to clarify that the purpose of this Figure is to document the successful generation of STAT5B knockout single cell clones across three human ALCL cell lines (SUPM2, SUDHL1, and DEL) following CRISPR/Cas9 editing.

The apparent absence of bands in several lanes does not reflect missing samples but rather represents the intended biological readout of the experiment, namely the loss of STAT5B protein expression in successfully edited single cell clones. Each lane corresponds to an individual single cell clone that was expanded after FACS sorting of GFP^{+} cells, and αTUBULIN is shown as a loading control. Clones showing strong αTUBULIN signal but absent STAT5B signal were therefore scored as STAT5B knockout clones and selected for downstream experiments.

We have updated **Fig. S7A** accordingly, added clone numbering for each cell line, and clarified in the legend body that it serves as a representative quality control documentation of the knockout strategy rather than a quantitative comparison between samples.

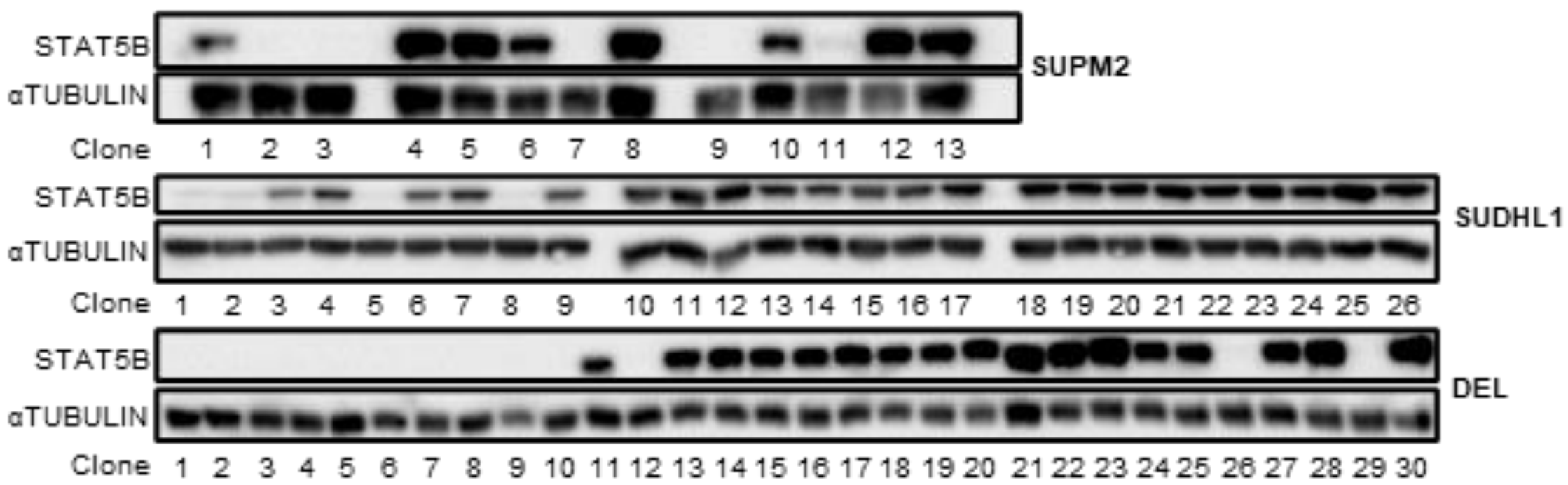

Figure R9: Representative Western blot analysis of STAT5B levels in single cell clones from 3 human ALCL cell lines. Numbers represent tested clones per cell line.

Fig. R9 is now **Fig. S7A** in the revised manuscript.

14) Figure S6C please show the western blot also for SU-DHL1 and SUP-M2 cell lines.

We thank the reviewer for this suggestion. We have now added representative Western blots for SU-DHL-1 and SUP-M2 to **Fig. S7C**, alongside the DEL data. Of note, we were unable to recover SU-DHL-1 DKO clones, consistent with the dependency described in the manuscript.

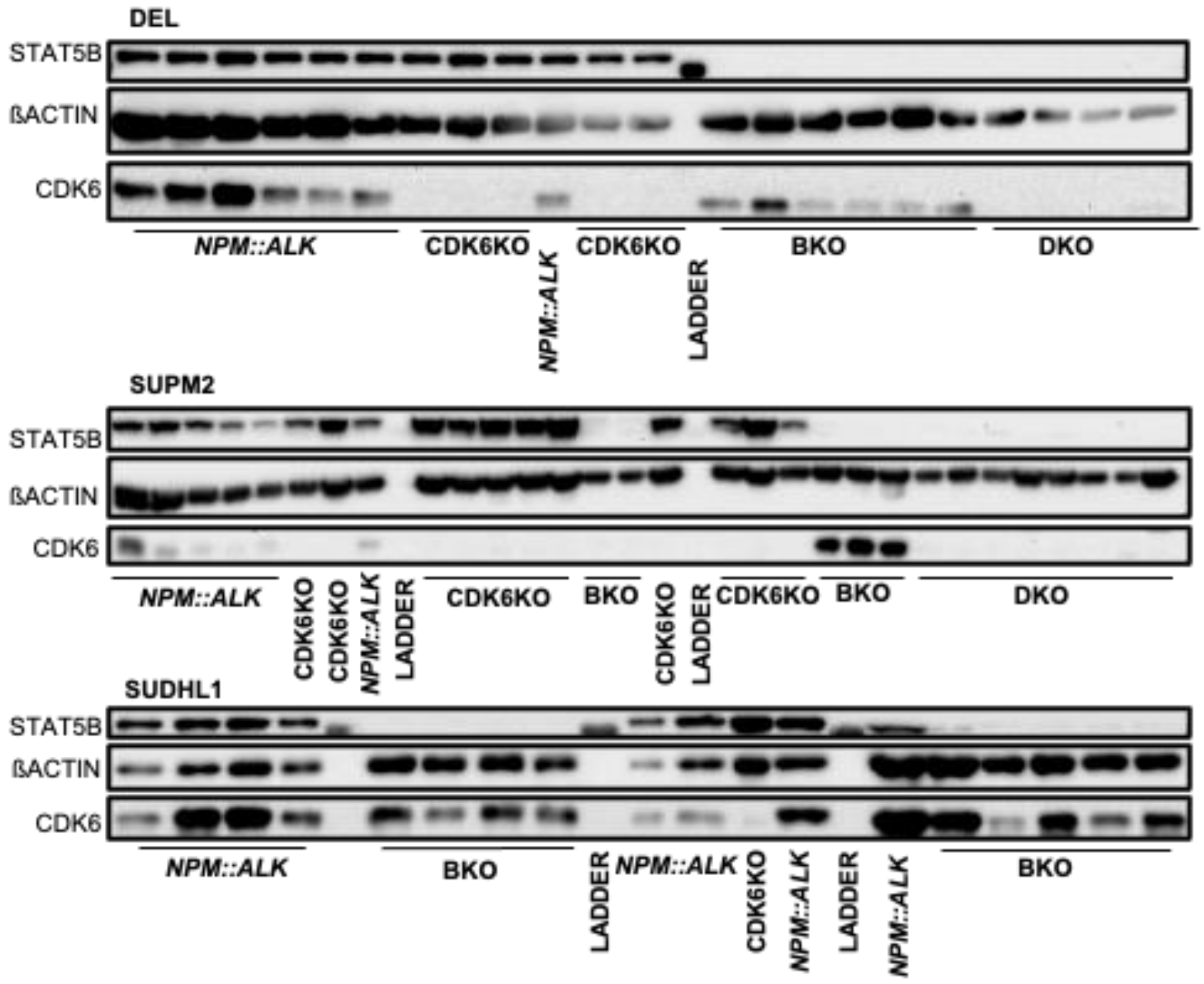

Figure R10: Representative Western blot analysis of CDK6KO and DKO cell lines confirming the loss of CDK6 and/or STAT5B in DEL, SUP-M2 and SU-DHL-1 cell lines.

Reviewer #2 (Remarks to the Author)

Dear Editor,

The manuscript in nice and elegant work to dissect the role CDK6 and STAT5B in NPM-ALK driven lymphoma. The Authors studied the effects of the genetic deletion of CDK6 and STAT5B in NPM-ALK transgenic mice. The absence of both CDK6 and STAT5B had a strong effect on the onset of such lymphoma, basically abrogating new lymphoma formation.

Large part of the work is focused on the development of T cells in the thymus, showing differences in cell maturation of T lymphocytes in the absence of both CDK6 and STAT5B, in particular at the DN2/3 stage. The Authors also studied the consequences of the genetic KO of CDK6 and STAT5B in human ALK+ ALCL lines, showing reduced cell growth and a synthetic lethality. In a limited series of ALK+ ALCL patients they found that a gene signatures potentially correlated with CDK6 and STAT5B could stratify the survival of patients.

The work is technically elegant, sound and well conducted, with the proper controls and experimental plan. The enthusiasm is somewhat diminished by considering that this work is incremental to what has been published by the same group in previous papers. Also, it is primarily focused on mouse T cell development, with little data from human lines or human samples. In it present form, the translatability of the findings to human ALK+ ALCL is unsure. The origin from a putative thymic precursor cell of ALK+ ALCL is still a controversial hypothesis in the field, thus these nice findings on mouse thymocytes might not readily translate into key biological findings in ALCL.

We thank the reviewer for the careful and constructive evaluation of our manuscript, and for the recognition that the work is "technically elegant, sound and well conducted, with the proper controls and experimental plan." We are also grateful for critical points raised, which have helped us sharpen the framing of our findings further and did our best to strengthen the translational value of our study. Below we respond point by point to the three central concerns raised in the Reviewer #2 summary.

1) We respectfully disagree with the characterization of this work as being only “incremental”, but we admit that often we did not present the validation of human ALCL patient samples in the main Figure part body, nor did we bioinformatically have sufficient patient subgroup analysis, which we try now in the revised version to improve to meet the critics. We would, however, also like to clarify the conceptual and mechanistic advances over our previous publications in more depth:

(i) The reviewer is likely referring to our earlier study by Kollmann et al. (2013)[15], which reported a survival benefit upon CDK6 loss in *NPM-ALK*$^{Tg/+}$ mice. However, the underlying transformation mechanism, the cell of origin, the cooperating signaling components, and in particular the cooperative roles of STAT5B were not investigated. Moreover, several key technical advances applied in our study, such as scRNA-seq analysis and modern bioinformatics approaches, were not yet available in 2013 and therefore cannot be considered trivial add-ons. Thus, the present work goes substantially beyond these earlier observations.

(ii) We show that loss of STAT5B alone significantly delays disease onset in *NPM-ALK*$^{Tg/+}$ mice **(Fig. 1A)**, a finding not previously described and consistent with the recent report highlighting the importance of a PDGFRB-STAT5A/B axis in this disease[13].

(iii) Combined deletion of CDK6 and STAT5B fully prevents thymic lymphoma formation across the observation window **(Fig. 1A-C)**. This cooperative requirement cannot be inferred from either single-gene phenotype, and this reveals that CDK6 and STAT5B are not functionally redundant but rather reciprocal acting oncoproteins that compensate for one another upon single-gene loss.

(iv) We defined the molecular basis of this cooperation through (a) integrated CDK6 and STAT5B ChIP-seq **(Fig. 4A, B, D, E; Fig. S4A, B, D** and **Fig. 5D, E)**; (b) coimmunoprecipitation **(Fig. 5F, G** and **Fig. S5I-M)**; and (c) demonstration of reciprocal pathway compensation in single-knockout murine cell lines and ALCL patient samples **(Fig. 6)**.

(v) Through scRNA-seq of pre-tumor thymocytes, trajectory analysis, reference mapping to a published murine thymic atlas, and prospective transplantation of $CD26^+$ DN2/3 cells into NSG mice **(Fig. 1D-I; Fig. 2E-G** and **Fig. 3A-C)**, we localize the tumor-initiating population to the DN2b/DN3a transition. To our knowledge, this is the first prospective experimental identification of a tumor-initiating cell at a single-cell resolution in the NPM-ALK model, offering new insight into an ongoing debate in the field.

(vi) The gene signature derived from the DKO-deregulated transformation program stratifies overall survival in ALCL patients independently of ALK status, bridging our developmental mechanism to a patient-relevant gene signature.

2) Our manuscript first addresses tumor development and, building on that, we sought to identify and validate potential therapeutic interventions. In the second part, we validated our key findings in at least one human ALCL-relevant model cell system, where we want to conclude that human patient sample analysis is often correlative, but mechanistic work requires often still genetic animal model investigation. This is an expensive, time consuming and demanding technical challenge that we tried to elegantly push forward in light of ALCL research for future patient benefit revealing new insights and possible new research angles that could inform innovative therapy like STAT5B and CDK6 combined protein degradation forward in ALCL, possibly relevant in relapsed patients that escape NPM-ALK inhibition. In the revised manuscript, we have substantially expanded the use of human datasets.

Specifically:

(i) We utilized scRNA-seq from a human ALK+ALCL biopsy to validate the importance of DKO deregulated genes within our identified NPM-ALK transformation program **(Fig. 4H-J)**

(ii) STAT5B and H3K27ac ChIP-seq from human ALK^+ALCL cell lines complement our report of direct STAT5B targets in the human disease and are cross-referenced with the murine ChIP-seq data throughout **(Fig. 4A, B, D, E and Fig. S4C, D)**

(iii) We defined the CDK6 interactome in human ALK^+ALCL cell lines by IP-MS **(Fig. 5G** and **Fig. S5 J-M)**.

(iv) In direct response to Reviewer #2's issue (see also below), we expanded our patient cohort for survival analysis from 25 to 74 ALCL patients (51 ALK^+ALCL, 23 ALK^-ALCL), and we tested

the independent prognostic value of our gene signature in one of the largest ALCL gene-expression datasets available to the public research communities **(Fig. 7H, I** and **Fig. S7D, E)**

Taken together, the mechanistic findings derived from our mouse model are extensively cross-validated in human ALK^{+}ALCL cell lines, in patient transcriptomes, in patient ChIP-seq data, and in patient survival outcomes. Thus, we are confident that the translational arc of the work is substantive, but we might have done a mistake to hide human ALCL relevant findings in Supplementary Figures, which we tried to better account for now in the revised manuscript version by adding human relevant data to main figures.

3) Our data, together with an increasing body of independent evidence, converge on an immature thymic-precursor origin for a substantial fraction of ALK+ALCL cases. We summarize this converging evidence below and place our findings in its context. Three independent lines of evidence support an immature thymocyte origin in our model: *NPM-ALK*$^{Tg/+}$ mice display specific expansion of the DN compartment at 8–10 weeks of age, prior to overt thymic disease **(Fig. 1C**). At a time-point at which the thymus/body-weight ratio is indistinguishable from WT, the *NPM-ALK*$^{Tg/+}$ DN compartment already contains tumor-associated cells that were absent in WT and DKO samples **(Fig. 1D, E)**. Unbiased reference mapping to a published murine thymus atlas places this cluster in DN3a (>1,500 cells), γδ T cells (~750 cells) and ISP (~500 cells), with virtually no cells mapping to mature T cell states **(Fig. 1H, I)**. Trajectory analysis independently positions the cluster as a transitory population emerging from DN2–DN3 thymocytes **(Fig. 3A, B)**.

The immature-thymocyte origin model is also supported by analyses of TCR rearrangement in patient material. Iyer et al. (2022)[16] used TCR clonotype patterns as a developmental clock, exploiting the ordered TCRγ, TCRβ, TCRα rearrangement during thymopoiesis. Oligoclonality at a given locus indicates that transformation occurred before clonal fixation at that stage. Across 67 ALCL samples (Crescenzo et al. cohort, predominantly ALK−)[17], they identified oligoclonality of TCRα, TCRβ, and TCRγ, placing ALCL transformation at the earliest immature thymocyte stage, before or at TCRγ rearrangement[15], corresponding to thymocytes around the murine DN2 stage.

Mapping the earlier ALK+ ALCL data of Malcolm et al. (2016)[18] onto this framework is particularly informative. Across 57 ALK+ ALCL tumors, 67% (TCRαβ and TCRγ-only categories) displayed clonal TCRα without a comparable major TCRβ rearrangement, a pattern not normally permitted during αβ thymic development. On Iyer's clock, the four Malcolm categories distribute across immature DN stages: TCR germline (14%) at DN1/ETP, TCRγ-only (11%) at DN2, the dominant TCRαβ category (56%) at the DN3 β-selection checkpoint, and TCRγδ (19%) at γδ lineage branching. This range, DN1 through DN3, corresponds closely to the DN2b/DN3a tumor-initiating and even lineage branching population, we identify in the

NPM-ALK mouse model, a pattern we also recapitulate in cell lines derived from end-stage murine tumors **(Fig. S2G, H)**.

Additional support comes from DNA methylation profiling, which demonstrates that ALK+ ALCL methylation patterns resemble immature thymic stages rather than mature peripheral T cells[19], and from the identification of an early thymic progenitor gene signature in tumor-propagating side-population cells from both ALK^+ and ALK^- ALCL[20]. Turner (2017)[21] has further argued that NPM-ALK signaling can substitute for pre-TCR signaling, allowing thymocytes to bypass β-selection and acquire an apparently mature, activated cell-surface phenotype despite their immature developmental origin, a framework that reconciles the immature origin of ALCL with its mature-looking presentation[22]. Consistent with this principle in a related disease setting, hyperactive STAT5B drives an ETP-ALL phenotype by hijacking TCR-pathway signaling even in the absence of surface TCR, producing cells with activated T-cell features despite their immature developmental origin[23]. This work is directly relevant to the heterogeneous CD4/CD8 patterns we observe in patients with progressed disease[24] and in our mouse model **Fig. 1B**. Supported by this, we want to acknowledge that ALK+ALCL is a heterogeneous disease influenced by clonal progression, age-associated metabolic changes or individual immunity consequences. Thus, a model has always limitations, which is also reflected by a human ALCL patient-derived model, where we want to conclude that no single model is likely to recapitulate every patient subgroup, but our genetically precise mouse model work highlights the vulnerable nodes in ALCL disease initiation and progression not done at such a mechanistic and molecular detail so far.

We have moderated absolute language in the manuscript and now frame our findings as supporting an immature-thymocyte origin complementing recent literature, while noting that further studies are needed to fully understand disease origin in ALCL.

We hope these clarifications address Reviewer #2's concern and that the additional analyses, combined with the responses to the overall reviewer and editorial team member comments, demonstrate that our work substantively advances the mechanistic and translational understanding of CDK6/STAT5B cooperation in ALK^+ALCL.

Major points:

- Gene signature and profiling should be repeated on human CDK6 and STAT5B KO cell lines or human samples with low CDK6 and STAT5B to determine the overlap seen with data obtained in mouse lines.

We thank the reviewer for raising this important point. We would respectfully like to direct the Reviewer #2's attention to **Fig. 6E**, **F**, where we performed this exact analysis on primary human ALCL patient samples. RNA sequencing data from human ALCL patients stratified by

CDK6 and STAT5 expression levels demonstrated that human samples with low CDK6 or low STAT5B expression showed deregulation of similar gene sets as were observed in the mouse lines in the context of *Cdk6* or *Stat5b* knockout (**Fig. 6A**).

- The stratification of ALK+ALCL patients into groups with distinct overall survival based on the identified CDK6/STAT5B transcriptional programs (Fig. 6H) is weak due to the small number of cases analyzed. The Authors should determine directly the expression levels of STAT5B and CDK6 by IHC and use it to stratify with survival in ALCL.

We thank Reviewer #2 for this suggestion and fully agree that direct protein-level validation would be a valuable complement to our transcriptional analysis. However, assembling a tissue cohort of sufficient size for a robust IHC-based survival analysis was not feasible within the time frame of this revision which would require further ethical protocol amendment filing and interaction with clinical groups from various countries to accomplish a more robust insight. The samples used in our analyses already represent a substantial fraction of the material we were able to access via many clinical collaborators that also obtained co-authorships, and we were unable to obtain additional well-annotated slides with linked survival data in the numbers required for a meaningful stratification analysis from our collaborators in given time frame. As an alternative, we have substantially extended our survival analysis based on gene expression data to substantiate the prognostic value of our gene signature and to assess its independent contribution. By integrating data from three independent ALCL cohorts ($n = 74$, 36 events), we demonstrated prognostic value independent of ALK status in multivariate Cox regression (continuous model: $HR = 3.35$, 95% CI: 1.04–10.80, $p = 0.043$; dichotomized model [High vs. Low]: $HR = 3.49$, 95% CI: 1.22–10.03, $p = 0.020$; concordance index ~0.71). We included the Kaplan-Meier with a tercile cut-off as well as a Forest plot of the continuous Cox model in **Fig. 7H, I** and the **Fig. R11C, D** as **Fig. S7D, E.**

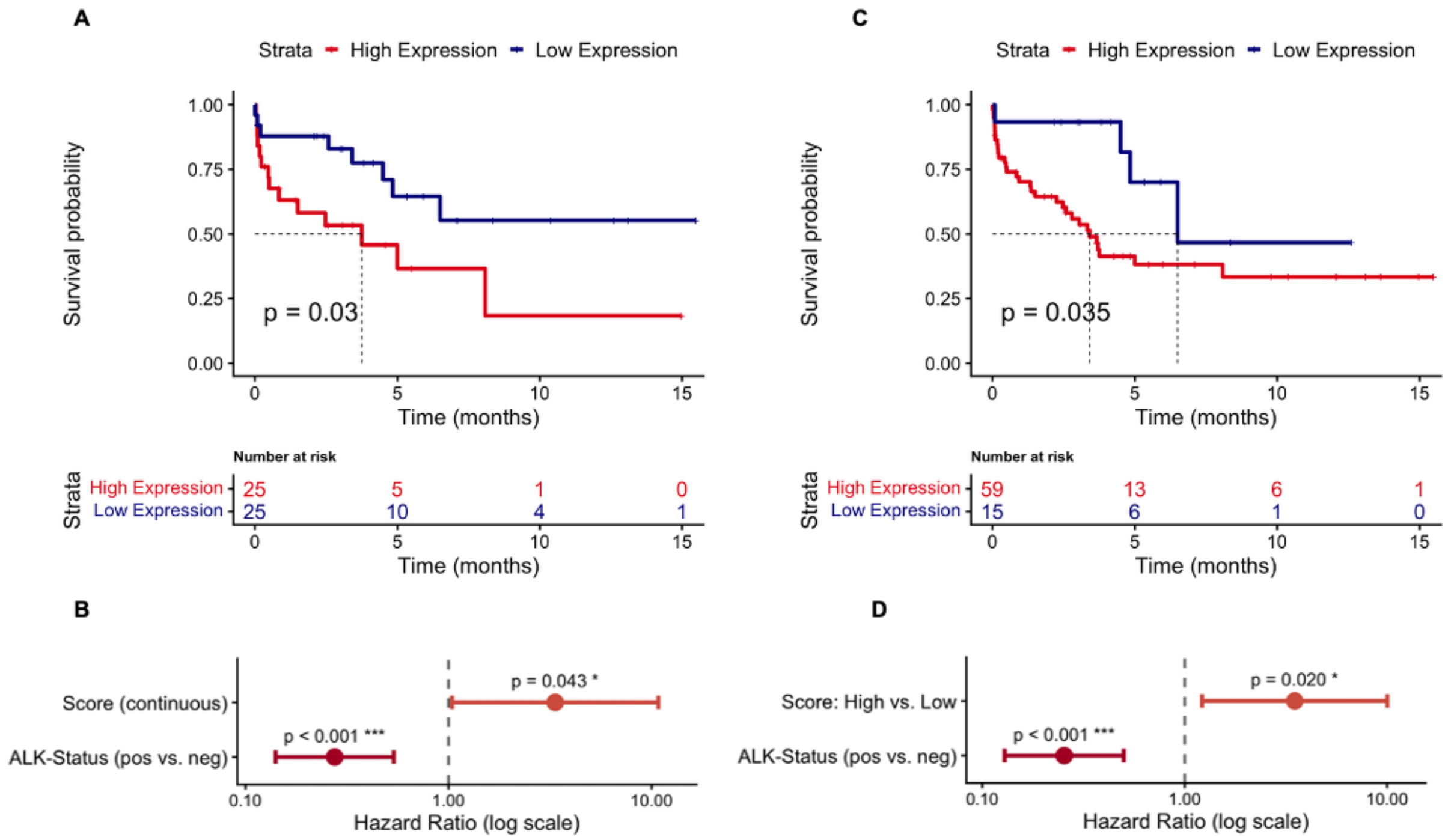

Figure R11: The 60-gene CDK6/STAT5B transcriptional signature stratifies ALCL patients by overall survival independently of ALK status.

*(A) Kaplan–Meier overall survival curves for ALCL patients (n = 74, 36 events; ALK+ = 51, ALK− = 23; three independent cohorts) stratified by high (red, top tercile) versus low (blue, bottom tercile) expression of the 60-gene CDK6/STAT5B signature; patients in the middle tercile were excluded (remaining n = 50). Log-rank p = 0.03. Tick marks indicate censored observations; the number-at-risk table is shown below. (B) Forest plot of the multivariate Cox proportional hazards model in the full cohort of 74 patients, including the signature score as a continuous variable and ALK status (positive vs. negative) as covariates. Points indicate hazard ratios; error bars indicate 95% confidence intervals; the dashed vertical line marks HR = 1. (C) Kaplan–Meier overall survival curves for the same combined cohort (n = 74) stratified by high (red) versus low (blue) signature expression. The optimal cutpoint was determined using the maximally selected rank statistic (maxstat, log-rank scoring, Lau92 p-value approximation). Log-rank p = 0.035. (D) Forest plot of the multivariate Cox model in the combined cohort with the signature dichotomized as high vs. low (HR = 3.49, 95% CI: 1.22–10.03, p = 0.020) and ALK status (positive vs. negative; HR = 0.25, 95% CI: 0.13–0.50, p < 0.001) as covariates; concordance index = 0.71. Significance levels: *p < 0.05, ***p < 0.001.*

<u>- Is the signatures observed in DKO lines specific for ALK+ ALCL or simply a signature deriving from the ablation of these two genes in any T cell lymphoma line?</u>

We thank the reviewer for raising this critical point. To address whether the DKO-derived signature reflects ALK^{+}ALCL-specific biology rather than a generic consequence of dual gene ablation in T cell lymphoma. Thus, we evaluated its prognostic performance in the extended ALCL cohort (n = 74; 51 ALK^{+} and 23 ALK^{-}) described above, and explicitly controlled for ALK status in a multivariate Cox regression analysis to meet statistics requirements for evaluation. Applied as a continuous score, the signature remained an independent predictor of overall survival after adjustment for ALK status (HR > 1, p = 0.043), arguing against it being a mere surrogate for ALK positivity. By extension, this independence from ALK status implies that the signature carries prognostic information that is, in principle, applicable across both ALK^{+} and ALK^{-}ALCL, where we worded the result and discussion section according to Reviewer #2 advice.

To further probe entity specificity, we additionally applied the signature to publicly available transcriptomic datasets of other peripheral T cell lymphoma entities from various mature PTCL entities, where it did not reach statistical significance for outcome stratification. While these analyses are limited by cohort size and heterogeneity and should therefore be interpreted with caution, they are consistent with the signature reflecting a biological program preferentially relevant to ALCL, both ALK+ or ALK-, but not for other PTCL subtype cancers. The data have been added as **Fig. S7F**.

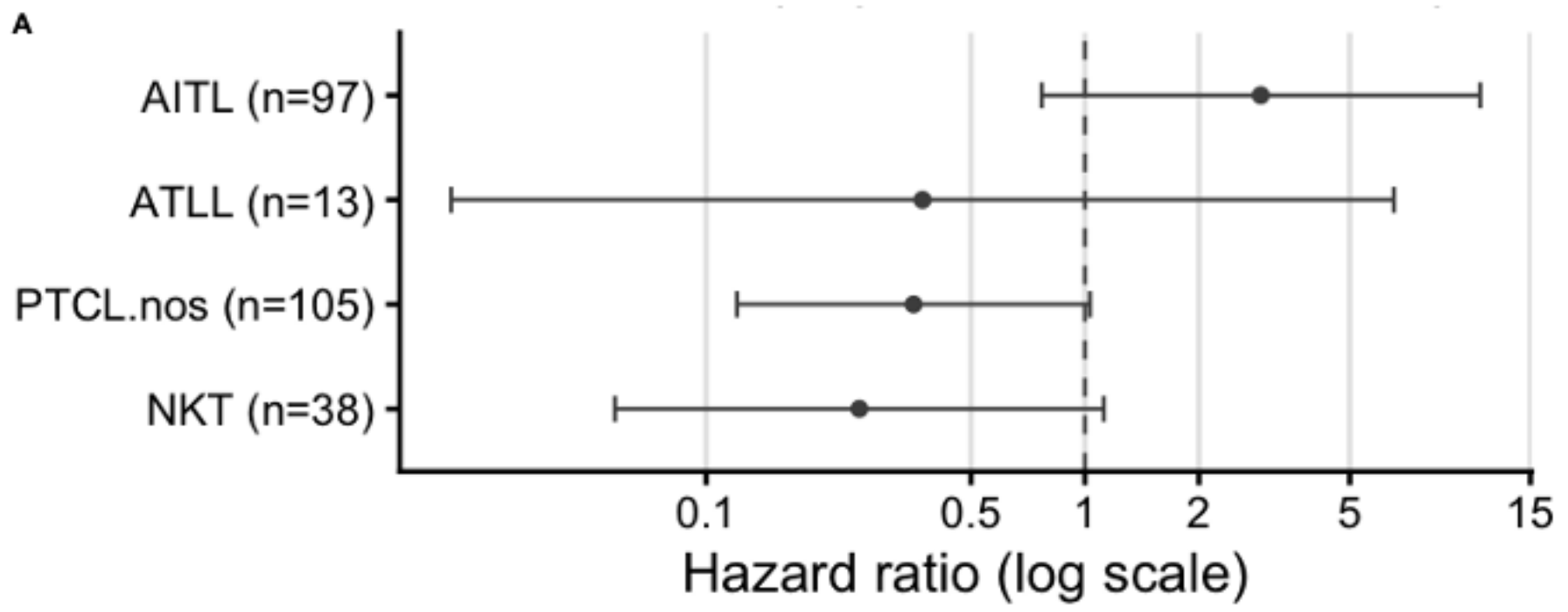

Figure R12: Association of the 60-gene signature score with overall survival across PTCL molecular subtypes.

(A) Forest plot of hazard ratios (HR) from univariate Cox proportional hazards models fitted independently within each subtype, with the per sample standardized gene set score as the sole covariate. Each row corresponds to one PTCL molecular subtype; sample sizes are given in parentheses next to the subtype label. Points indicate HR point estimates and horizontal bars span the 95% Wald confidence interval. The vertical dashed line marks HR = 1 (no association); HR < 1 indicates that higher gene set activity is associated with longer overall survival, while HR > 1 indicates the opposite. The hazard ratio axis is shown on a base 10 logarithmic scale. Subtypes are ordered by HR point estimate from top to bottom.

Reviewer #3 (Remarks to the Author)

This manuscript investigates the earliest oncogenic events in ALK$^+$ anaplastic large cell lymphoma using a genetic model of NPM-ALK mouse model combined with single-cell transcriptomics, chromatin analyses, transplantation, and human validation. CDK6 and STAT5B act as cooperative, non-redundant drivers of transformation specifically at the DN2/3 thymocyte stage, enforcing a tumor-specific developmental trajectory through shared transcriptional and chromatin programs. Dual loss of CDK6 and STAT5B completely blocks tumor initiation, induces apoptosis, and reveals a context-dependent synthetic lethal vulnerability with prognostic relevance in patients. The study is very impactful for the field.

Below, I am outlining a few suggestions that might help clarify aspects of the study:

1) DN2/3 thymocytes are the initiating population; can this be shown independently of scRNA-seq inference and transplantation? I suggest that the group performs limiting-dilution transplantation of purified DN subsets.

We thank the reviewer to acknowledge that we analyzed the earliest oncogenic events in ALK$^+$

anaplastic large cell lymphoma using both genetic mouse model analysis combined with single-cell transcriptomics, chromatin analyses, transplantation, and human validation". We appreciate the reviewer suggestion to perform limiting dilution transplantation experiments as an elegant additional approach to further confirm the DN2/DN3 origin of the disease. However, we feel that such an experiment is beyond the scope of the present study and not feasible within a reasonable timeframe of the revision due to animal ethics filing in Austria. We have reached our limit for ethical animal license numbers and would need to write an entirely new animal license, which we could only carefully do in future work due to our mission to work along the 3R principles. In the bulk transplantation experiment already shown in **Fig. 2E**, NSG recipients were injected with a deliberately low number of cells (80,000) and required an observation period of more than 300 days to develop disease. A limiting dilution assay in wildtype recipients would therefore further prolong both engraftment and disease latency which would go beyond a possible two-year time frame. The first author of the manuscript is a PhD student, and the reviewer can consider the amount of work and money required to conduct the presented work. Thus, at this point we feel that the number of animals and the timeline required are not justified to carry out such details to gain delayed further insights.

Nevertheless, we tried to gather further evidence of tumor initiation based on DN2/DN3. Therefore, as an alternative information, we analyzed along the reviewer comments the cell-cycle stages of WT and *NPM-ALK*$^{Tg/+}$ DN1 to DN4 thymocytes and found that *NPM-ALK*$^{Tg/+}$ cells already display increased proliferation at the DN2 stage, which we highlight now in the revised manuscript version as **Fig. S2F**.

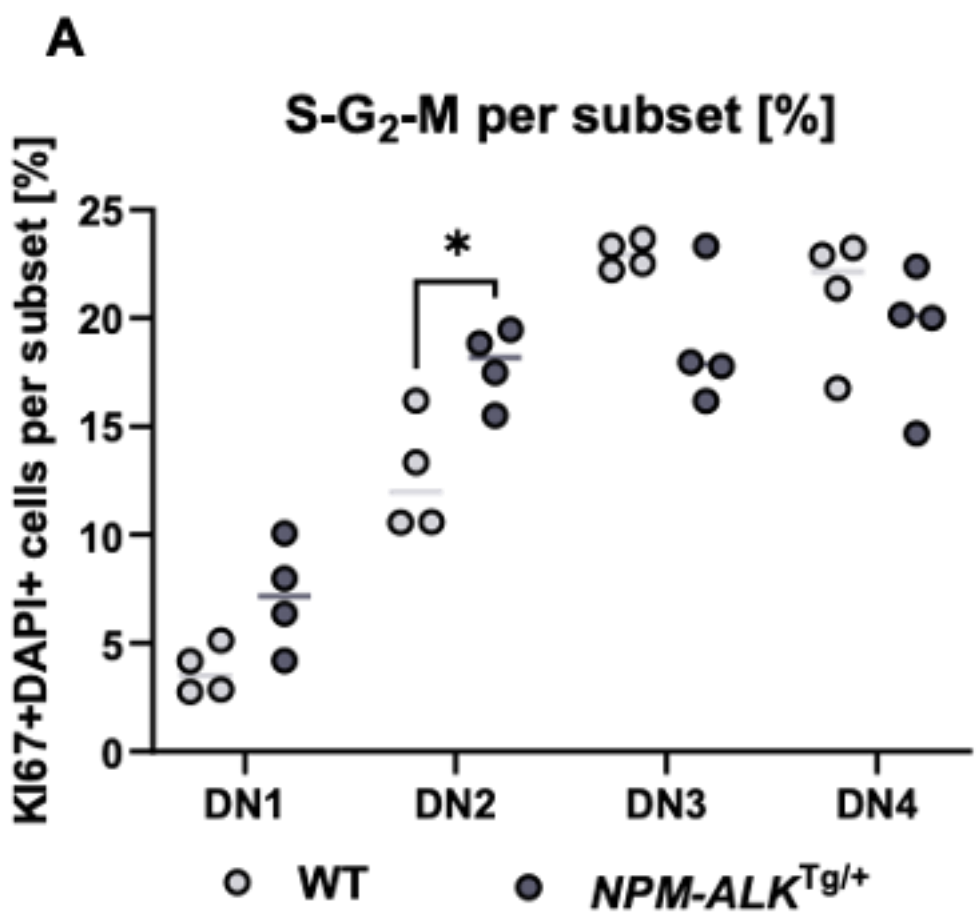

Figure R13: Increased proliferation of NPM-ALK$^{Tg/+}$ *thymocytes at the DN2 stage.*

(A) Frequency of cycling cells (S-G2-M phase, defined as KI67+DAPI+) within each double negative thymocyte subset, assessed by flow cytometry. Each symbol represents an individual mouse and horizontal lines indicate the median. Statistical analysis was performed by ordinary two-way ANOVA with Sidak's multiple comparisons test ($p < 0.05$).*

The Figure was added to the revised manuscript as **Fig. S2F.** The manuscript text was amended accordingly in line 132-134.

2)Hematopoietic-intrinsic effect: To fully exclude niche effects, the authors should ideally perform competitive thymic reconstitution assays. discuss.

We thank the reviewer for this point. We agree that ruling out a contribution of the niche is important, but we feel that with the colony formation assay **(Fig. S1C**, with controls in **Fig. S1A/B)** and the *in vivo* transplantation experiment **(Fig. 2E)** we already have strong evidence demonstrating a hematopoietic-intrinsic phenotype which does not necessarily require a specific niche effect.

While the suggested competitive thymic reconstitution assay in immunocompetent hosts would be a further refinement, such an experiment would require generation and breeding of donor and competitor cohorts across multiple genotypes, lethal irradiation of recipient mice, long-term reconstitution of the hematopoietic and thymic compartments, and prolonged observation for tumor onset, collectively requiring a substantial number of animals and an extended timeline. In addition, it would require the writing and filing for a new animal ethical license that needs approval by the university ethical committee, the ethical government animal law authorities and finally the ministry/university committee's approval, all time consuming and demanding steps in Austria. Last but not least due to the severity grade of the cancer models analyzed and due to the suffering involved in animal experimentation with tumor-bearing mice we have to first perform a post-analysis of performed work in Austria called "Rückblickende Bewertung". We are obliged by EU and national animal law to work along the 3R principles and both the severity grading of the animals and the questionable time it takes to carry out the work with further manipulation of both donor and recipient mice would require strong argumentation that is better suited for future work. Simply, we are not able in the given time frame to file another ethical animal license for uncompleted new work. Therefore, we hope for the understanding of the reviewer that such an experiment is beyond the scope of this revision.

3)Is the kinase activity of CDK6 necessary for the role of CDK6 and cooperativity with STAT5B activity or its transcriptional/scaffolding role? The authors can perform rescue studies using WT or kinase-dead CDK6. Additionally they could perform a comparison of the molecular signatures induced by CDK6 degrader and the CDK4/6 inhibitor.

To dissect the importance of kinase-dependent and kinase-independent functions of CDK6 for ALK+ ALCL development, we made use of *NPM-ALK*$^{Tg/+}$*Cdk6*^{K43M} mice, expressing a catalytically inactive CDK6[25]. As shown in the Kaplan-Meier survival curve **(Fig. R14A)**, *NPM-ALK*$^{Tg/+}$*Cdk6*^{K43M} mice show a significant delay in disease onset compared to

NPM-ALK$^{Tg/+}$ controls with a similar latency as *NPM-ALK*$^{Tg/+}$*Cdk6*$^{-/-}$ animals. This demonstrates a dominant kinase-dependent role of CDK6 for tumor establishment at least in the presence of STAT5B.

Besides the canonical kinase function of CDK6, our previous studies in BCR::ABL1 leukemic cells suggest that CDK6 kinase activity does however not influence its chromatin interaction potential. Kinase-inactive and wildtype CDK6 show overlapping ChIP-seq binding patterns[26,27].

To further characterize the CDK6 interactome of transcriptional regulators in kinase-active and inactive contexts, we treated human ALK^{+} SUDHL and DEL cell lines with Palbociclib and carried out a CDK6 pulldown followed by mass spectrometry. To characterize the core interaction partners of CDK6 we overlapped the results from the two cell lines and found 129 common CDK6 interactors **(Fig. R14B, C** now **Fig. S5J, K)**. STRING network analysis revealed several transcriptional regulators to be present in CDK6-containing complexes. Enrichment analysis highlighted RNA polymerase II preinitiation complex (PIC) assembly, transcription preinitiation complex assembly, protein-DNA complex assembly, and transcription factor TFIID complex as the most significantly enriched terms **(Fig. R14D, E** now as: **Fig. S5L, M and Fig. R14F** now as: **Fig. 5G)**. Palbociclib treatment did not result in major interaction changes of these core interactors which is in line with our previous findings. Thus, we thank the reviewer for this suggestion and our data demonstrates that kinase inactive CDK6 shows not only interaction with similar DNA *loci* as wildtype CDK6, but it also acts in similar transcriptional complexes.

Besides CDK6 kinase-independent interaction potential, the lost kinase activity might lead to similar gene expression patterns and cellular phenotypes as cells lacking CDK6, underscored by the *NPM-ALK*$^{Tg/+}$*Cdk6*^{K43M} mice phenotype.

To test if CDK6 kinase function is essential for apoptosis onset, which is described as one critical mechanism in the *NPM-ALK*$^{Tg/+}$*Cdk6*$^{-/-}$*Stat5b*$^{-/-}$ cells **(Fig. 5C** and **Fig. S5G, H)**, we set out to treat wildtype and STAT5B knockout ALCL cell lines with palbociclib or BSJ-03-123 and to analyze apoptosis onset by flow cytometry. We first determined the degradation efficacy of the CDK6 degrader, as this can be counteracted by CDKN binding to CDK6[14]. SU-DHL-1 cells show virtually no significant degradation of CDK6, which is accompanied by high CDKN levels that likely prevent degradation (**Fig. R15A**). The scatter plot of CDK6 IP-MS LFQ intensities confirms strong association of CDK6 with CDKN1A, CDKN1B and CDKN2A in both untreated and palbociclib-treated conditions, consistent with CDKN shielding from PROTAC-induced degradation (**Fig. R15B**). In contrast, DEL cells showed a better degradation efficacy (**Fig. R15C**), which is in line with the absence of CDKN interactors found in the CDK6 IP-MS and no p16 expression in the DEL cell lines.

CDK6 degradation in DEL cells showed a significant increase in apoptosis in STAT5B knockout cells compared to wildtype cells, which was not mimicked by palbociclib, highlighting a largely kinase-independent role of CDK6 in combination with STAT5B loss to induce apoptosis.

Notably, degradation in DEL cells was less efficient in the BKO background than in WT: BSJ-03-123 reached statistical significance in WT ($P < 0.05$, two-way ANOVA with Sidak's multiple comparisons test) but only trended downward in BKO ($P = 0.16$, ns; **Fig. R15C**). This incomplete and genotype-dependent degradation efficacy is a phenomenon specific to the human cell line system and a similar finding was not observed in our murine setting. Despite the weaker CDK6 reduction in BKO cells, BSJ-03-123 still produced a highly significant apoptotic response selectively in this genotype ($P = 0.0004$; **Fig. R15D**), whereas WT cells did not significantly respond, suggesting that even partial CDK6 degradation is sufficient to drive the phenotype and that a more complete CDK6 reduction would likely amplify it further.

Taken together we observed that while the scaffolding function of CDK6 does not depend on its kinase activity, deleting CDK6 kinase function strongly delays tumor onset in the presence of STAT5B. In the context of STAT5B loss, we have indication that the effects of CDK6 on apoptosis are enhanced upon full CDK6 degradation compared to kinase inhibition alone. We have therefore sufficient experimental evidence to suggest that kinase-dependent and -independent roles of CDK6 synergize to fully prevent tumor development in absence of STAT5B.

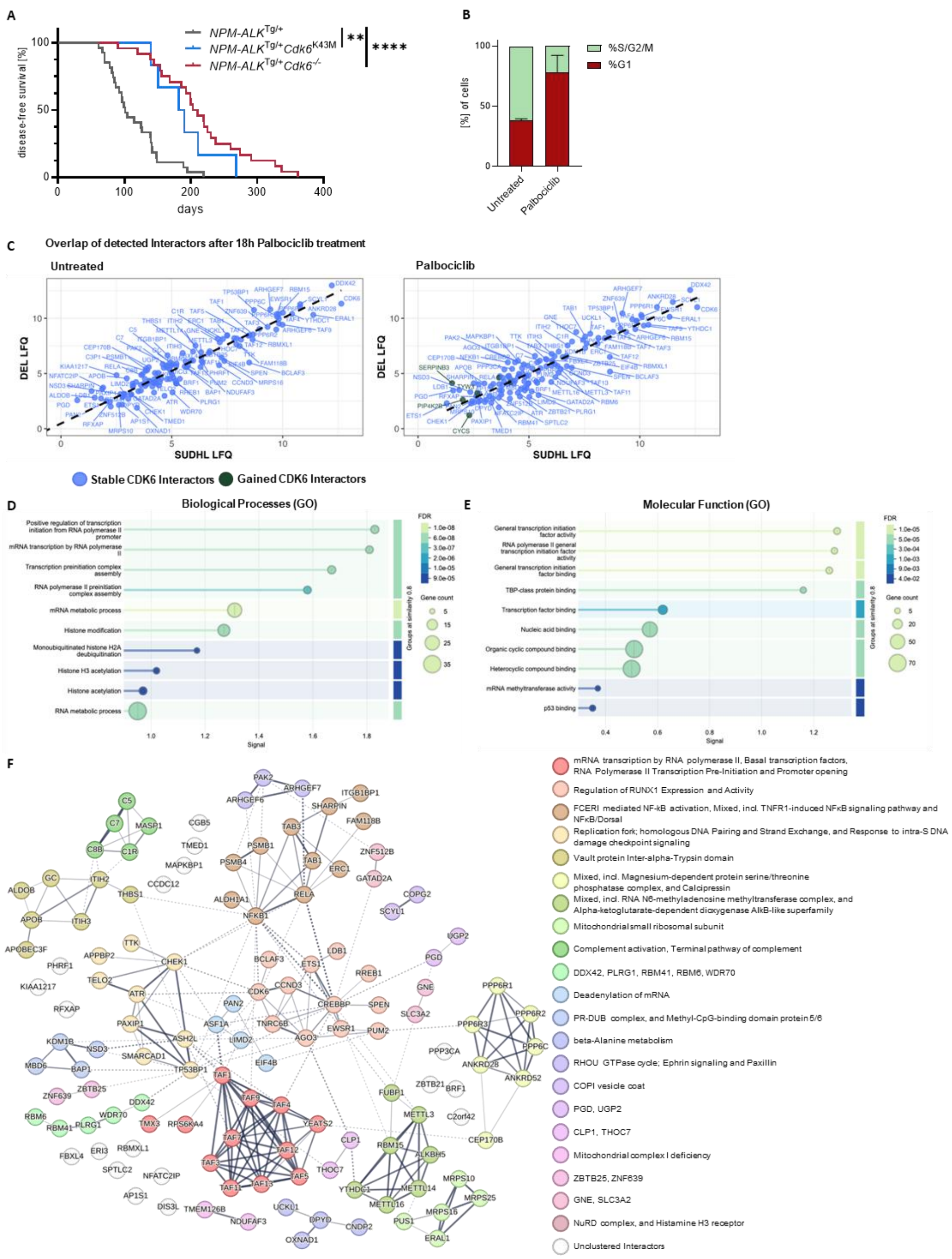

*Figure R14: (A) Kaplan–Meier plot of NPM-ALK$^{Tg/+}$ (grey), NPM-ALK$^{Tg/+}$Cdk6^{K43M} (blue) and NPM-ALK$^{Tg/+}$Cdk6$^{-/-}$ (red) mice. ** $p < 0.01$, **** $p < 0.0001$, log-rank test. (B) Cell cycle distribution of SU-DHL-1 and DEL cells (combined, mean ± SD) ± 18 h palbociclib. (C) IP-MS CDK6 interactors in SUDHL1 vs DEL cells, untreated (left) and palbociclib-treated (right). Stable interactors (n = 129, blue) and gained interactors (n = 6, dark green) are highlighted; dashed lines indicate linear regression fits. (D, E) STRING GO enrichment of the 129 stable interactors: (D) Biological Process, (E) Molecular Function. Dot size = gene count, color = FDR (Benjamini–Hochberg), right-hand bar = redundancy grouping at similarity 0.8, x-axis = STRING enrichment signal. (F) STRING PPI network of the 129 stable interactors (confidence ≥ 0.4), clustered by MCL (inflation = 2). Edge thickness = combined interaction score; solid = within-cluster, dashed = between-cluster. Node color = MCL cluster; functional annotations are listed at right. Unclustered nodes are shown in white.*

We added **Fig. R14B, C** now as **Fig. S5J, K; Fig. R14D, E** now as: **Fig. S5L,M** and **Fig. R14F** now as: **Fig. 5G** to the revised manuscript to account for suggested changes along Reviewer #3's advice**.**

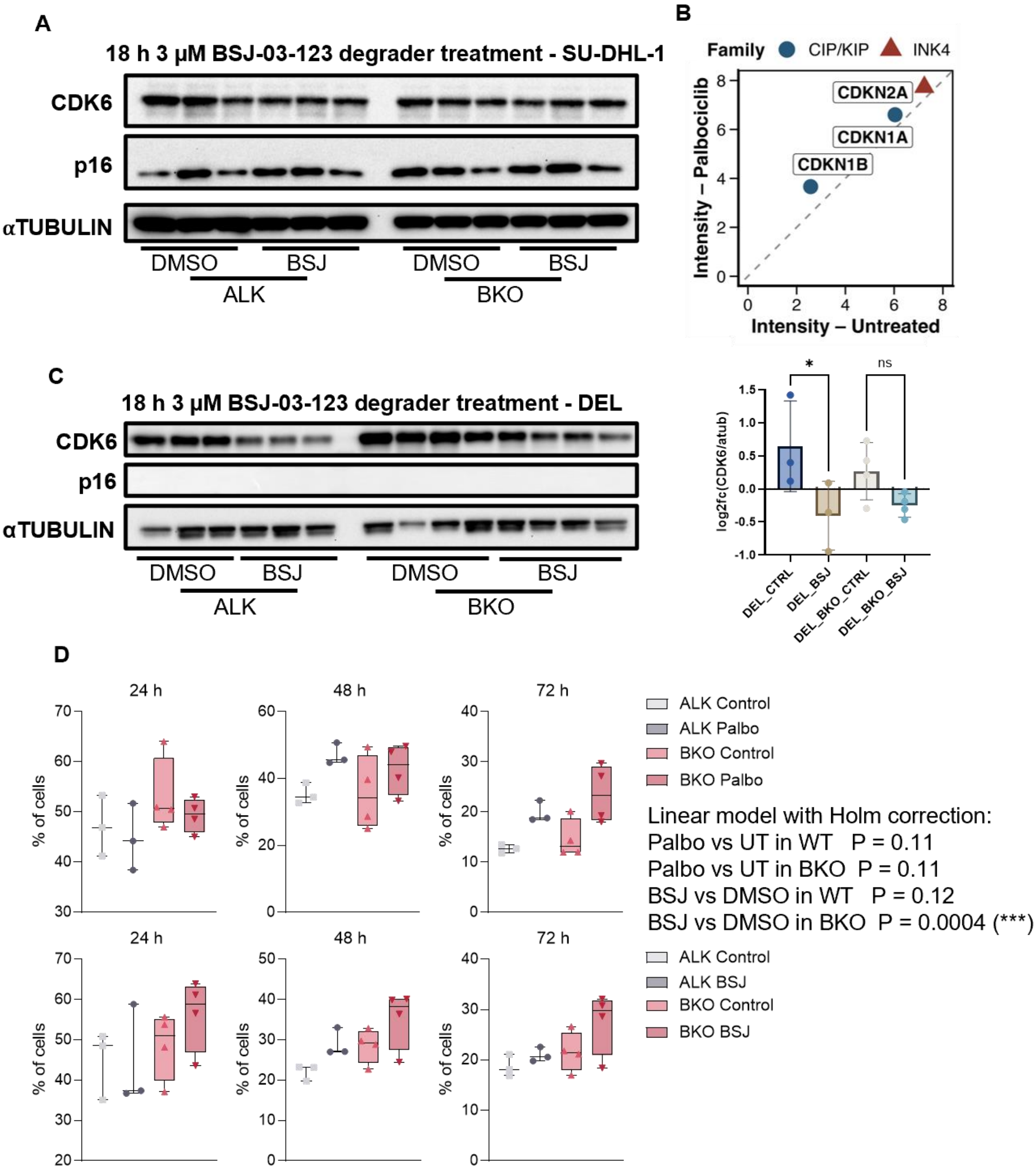

Figure R15: Pharmacological CDK6 inhibition and degradation phenocopy CDK6 loss in a STAT5B dependent manner only partially, in line with incomplete protein reduction.

(A) Western blot of CDK6, p16^{INK4A} and α-tubulin (loading control) in SU-DHL-1 after 18 h of treatment with 3 µM of the BSJ-03-123 CDK6 PROTAC degrader or DMSO vehicle control, in ALK (parental) and BKO (STAT5B knockout) backgrounds. (B) Scatter plot of CDK6 IP-MS LFQ intensities for CDKN1A, CDKN1B (CIP/KIP family) and CDKN2A (INK4 family) in untreated versus palbociclib-treated SU-DHL-1 cells. The dashed diagonal indicates equal intensity. (C) Validation of CDK6 protein levels after BSJ-03-123 treatment in DEL cells. Left: representative Western blot for CDK6 and the loading control α-tubulin in ALK and BKO DEL cells treated for 18 h with DMSO or 3 µM BSJ-03-123. Right: quantification expressed as log2 fold change of CDK6 over α-tubulin. Each dot represents one independently cultured clone replicate; bars show mean with standard deviation. Statistical analysis was performed by ordinary two-way ANOVA comparing BSJ treated samples to their control with Sidak's multiple comparisons test ($P < 0.05$; ns, not significant). (D) Apoptosis assessment by Annexin V/7AAD staining in WT (ALK) and STAT5B knockout (BKO) DEL ALCL cells at 24, 48 and 72 hours. Top row: untreated control versus 200 nM palbociclib (Palbo). Bottom row: vehicle control (DMSO) versus 3 µM BSJ-03-123 (BSJ). Box plots show the apoptotic cell fraction with min to max range and all individual data points; n = 3 WT and n = 4 BKO replicates per condition. P values are from a linear model fitted across all three timepoints,*

*with replicate as a covariate, and Holm corrected across the four planned contrasts; the control condition was H_2O for the palbociclib arm and DMSO for the BSJ arm (*** $P < 0.001$).*

<u>4)Direct regulation of the chromatin effect: can the authors test the effect via ATAC-seq or CUT&RUN/CUT&Tag (e.g. H3K27me3, H3K4me3) in DN cells and cross-comparison to data in human systems?</u>

We thank the reviewer for this suggestion. We address it through an integrative analysis combining transcription factor binding, multiple histone modifications, and cross-species validation in the developmentally relevant cell populations.

We made use of the ImmGen histone modification compendium that represents the most comprehensive chromatin resource available for these specific developmental stages and directly informs on chromatin state, including marks of active transcription, Polycomb-mediated repression, and insulator function at CDK6/STAT5B target loci. Together with our ChIP-seq data and the human H3K27ac analysis[28], this integrative approach captures both regulatory factor binding and chromatin state in the biologically relevant cell populations.

We had previously performed CDK6 and STAT5B ChIP-seq and overlapped these peaks with promotors of both, chromatin organization genes and dynamically regulated "shift genes" identified during the DN2 to DN3 transition, establishing direct binding of both factors at these loci. We now integrated six histone modification profiles (H3K4me3, H3K27ac, H3K4me1, H3K36me3, H3K27me3, and CTCF) from ImmGen DN2a and DN3 pro-T cells (**Fig. R16A**), the precise developmental stages in which the transcriptional shifts occur. CDK6/STAT5B co-bound chromatin organization genes are consistently enriched for activating promoter and enhancer marks (H3K4me3, H3K27ac, H3K4me1) and for the transcriptional elongation mark H3K36me3, defining a chromatin landscape of actively regulated genes. In contrast, at shift gene promoters, CDK6/STAT5B binding is associated with reduced H3K27me3 levels relative to unbound shift genes, consistent with these factors occupying loci that have escaped or been released from Polycomb-mediated repression during the DN2 to DN3 transition. CTCF enrichment is most pronounced at CDK6/STAT5B co-bound chromatin organization gene promoters in DN3, and particularly at STAT5B-bound loci, raising the possibility that higher-order chromatin organization and insulation contribute to the regulation of these targets. Together, these chromatin patterns observed across DN2 and DN3 thymocytes support a model in which CDK6/STAT5B binding occurs within a permissive and architecturally organized chromatin landscape during the relevant developmental window.

Finally, we extended these findings to human systems by integrating H3K27ac ChIP-seq data from primary ALCL patient biopsies and ALK^{+}ALK cell lines[28]. STAT5B/CDK6 co-bound genes

show consistent enrichment for active chromatin marks across multiple human contexts, supporting cross-species conservation of this regulatory mechanism (**Fig. R16B**).

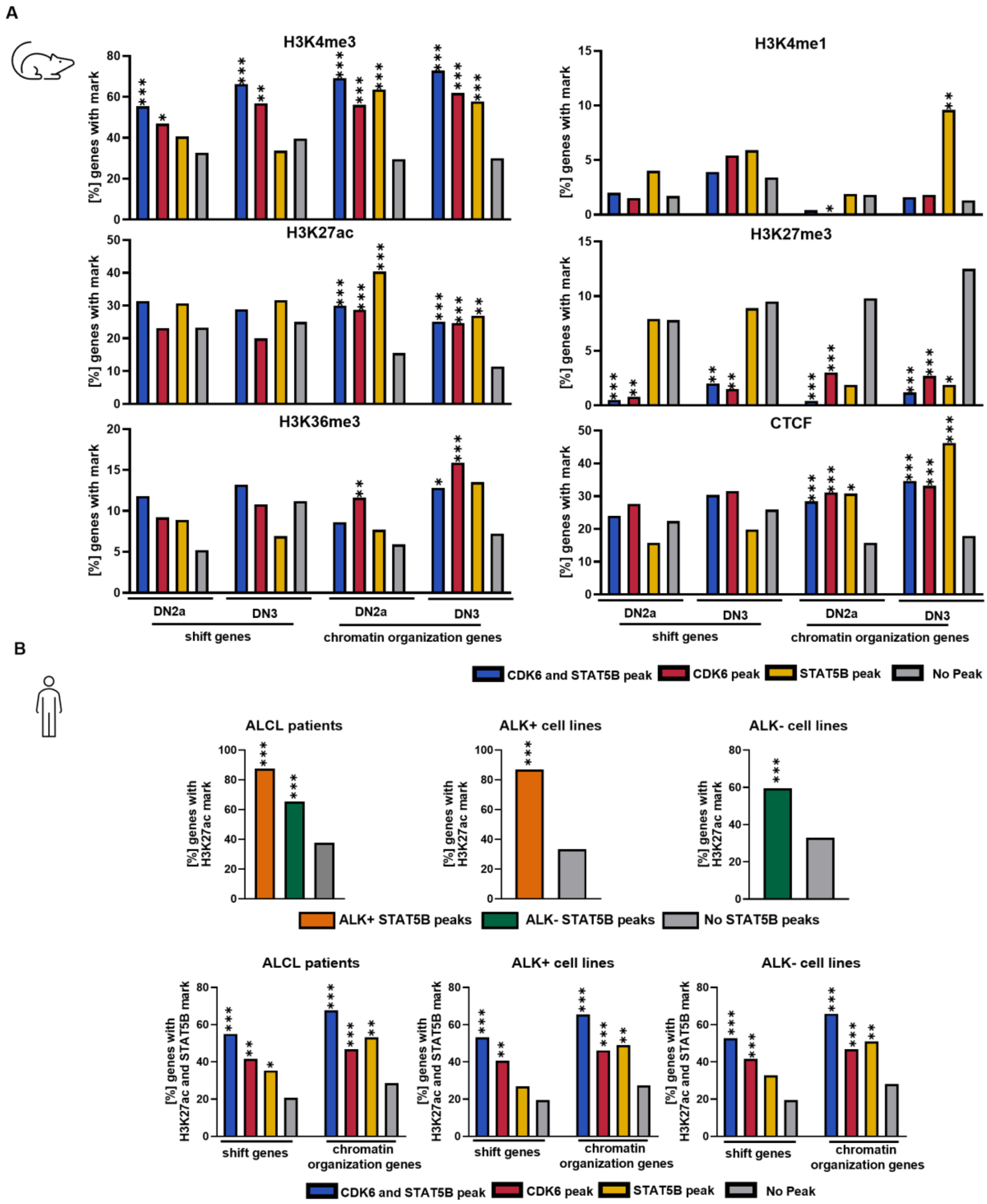

Figure R16: Promoter chromatin mark enrichment at CDK6- and STAT5B-bound genes in murine pro-T cells and human ALCL.

*(A) Bar graphs showing the percentage of murine shift genes (left pair of x-axis groups) and chromatin organization genes (right pair of x-axis groups) carrying the indicated promoter-proximal chromatin mark (H3K4me3, H3K4me1, H3K27ac, H3K27me3, H3K36me3, CTCF) in DN2a and DN3 pro-T cell progenitors from the ImmGen database. Promoter occupancy was defined as any ChIP-seq peak falling within a ±1000 bp window centered on the TSS. Genes are stratified by CDK6 and STAT5B ChIP-seq occupancy at their promoters: co-bound by CDK6 and STAT5B (Both; blue), CDK6-bound only (red), STAT5B-bound only (yellow), or unbound by either factor (No Peak; grey). Asterisks above bars denote statistical significance of a two-sided Fisher's exact test comparing each binding group to the No Peak reference group (*p < 0.05, **p < 0.01, ***p < 0.001, uncorrected).*

*(B) Upper panels: Bar graphs showing the percentage of shift genes and chromatin organization genes with a STAT5B ChIP-seq peak at their promoter (TSS ±1000 bp) in primary ALCL patient samples, ALK$^+$ ALCL cell lines (SU-DHL-1, DEL, SUP-M2, Karpas-299, SR-786), and ALK− ALCL cell lines (Mac-1, Mac-2a), stratified by STAT5B binding group: ALK$^+$ STAT5B peaks (orange), ALK$^-$ STAT5B peaks (green), or no STAT5B peak (grey). Asterisks denote two-sided Fisher's exact test versus the No STAT5B Peak reference (***p < 0.001, uncorrected).*

*(C) Bar graphs showing the percentage of shift genes (left within each panel) and chromatin organization genes (right within each panel) carrying concurrent H3K27ac mark and STAT5B ChIP-seq occupancy at their promoters (TSS ±1000 bp) in primary ALCL patients, ALK$^+$ cell lines, and ALK$^-$ cell lines. Genes are stratified by CDK6 and STAT5B ChIP-seq co-occupancy: co-bound by CDK6 and STAT5B (Both; blue), CDK6-bound only (red), STAT5B-bound only (yellow), or unbound (No Peak; grey). Asterisks indicate two-sided Fisher's exact test versus the No Peak reference group (*p < 0.05, **p < 0.01, ***p < 0.001, uncorrected).*

We added Fig. R16 to the new main **Fig. 4D, E** and to **Fig. S4C, D**.

<u>5)What is the independent prognostic value of the 50-gene signature the authors identified? Can the authors test this using validation in different cohorts if available?</u>

During the course of this revision, we have substantially extended our survival analysis. By integrating data from three independent ALCL cohorts (n = 74, 36 events), we demonstrated a prognostic value of our gene signature independent of ALK status in multivariate Cox regression (continuous model: HR = 3.35, 95% CI: 1.04–10.80, p = 0.043; dichotomized model [High vs. Low]: HR = 3.49, 95% CI: 1.22–10.03, p = 0.020; concordance index ~0.71). We included the Kaplan-Meier with a tercile cut-off as well as a Forest plot of the continuous Cox model in **Fig. 7H, I** and the **Fig. R17C, D** as **Fig. S7D, E**.

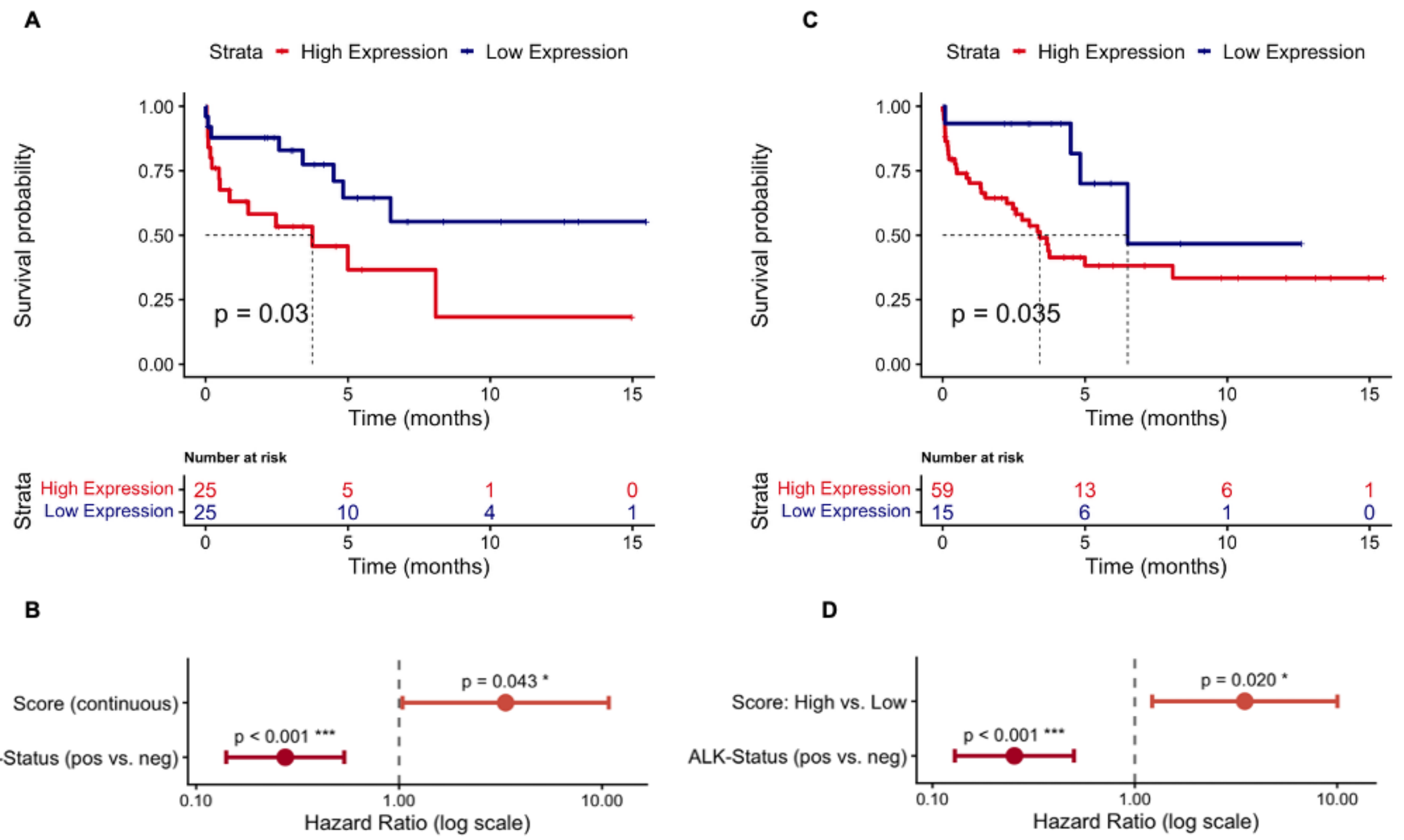

Figure R17: The 60-gene CDK6/STAT5B transcriptional signature stratifies ALCL patients by overall survival independently of ALK status.

(A) Kaplan–Meier overall survival curves for ALCL patients (n = 74, 36 events; ALK+ = 51, ALK− = 23; three independent cohorts) stratified by high (red, top tercile) versus low (blue, bottom tercile) expression of the 52-gene CDK6/STAT5B signature; patients in the middle tercile were excluded (n = 50). Log-rank p = 0.03. Tick marks indicate censored observations; the number-at-risk table is shown below. (B) Forest plot of the multivariate Cox

*proportional hazards model, including the signature score as a continuous variable and ALK status (positive vs. negative) as covariates. Points indicate hazard ratios; error bars indicate 95% confidence intervals; the dashed vertical line marks HR = 1. (C) Kaplan–Meier overall survival curves for the same combined cohort (n = 74) stratified by high (red) versus low (blue) signature expression. The optimal cutpoint was determined using the maximally selected rank statistic (maxstat, log-rank scoring, Lau92 p-value approximation). Log-rank p = 0.035. (D) Forest plot of the multivariate Cox model in the combined cohort with the signature dichotomized as high vs. low (HR = 3.49, 95% CI: 1.22–10.03, p = 0.020) and ALK status (positive vs. negative; HR = 0.25, 95% CI: 0.13–0.50, p < 0.001) as covariates; concordance index = 0.71. Significance levels: *p < 0.05, ***p < 0.001.*

To further probe entity specificity, we additionally applied the signature to publicly available transcriptomic datasets of other peripheral T cell lymphoma entities, where it did not reach statistical significance for outcome stratification. While these analyses are limited by cohort size and heterogeneity and should therefore be interpreted with caution, they are consistent with the signature reflecting a biological program preferentially relevant to ALCL. The data have been added to **Fig. S7F**.

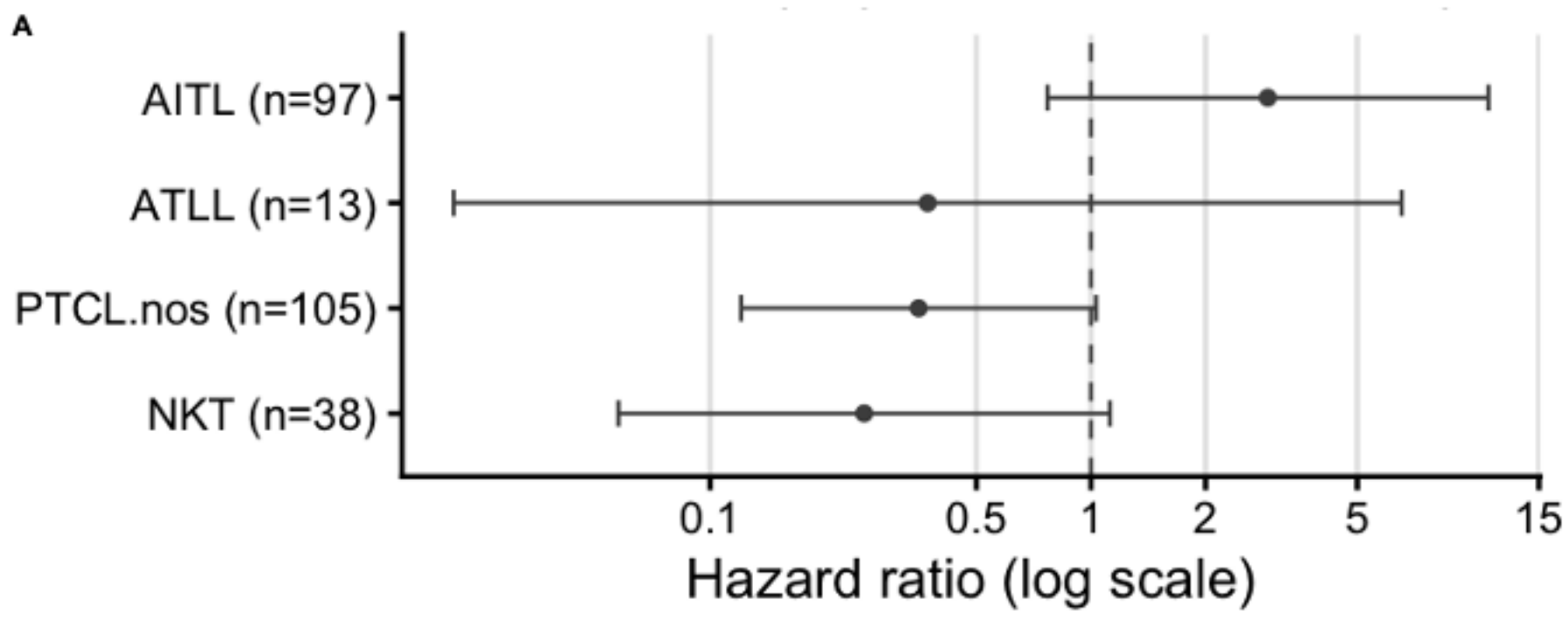

Figure R18: Association of the 60-gene signature score with overall survival across PTCL molecular subtypes.

(A) Forest plot of hazard ratios (HR) from univariate Cox proportional hazards models fitted independently within each subtype, with the per sample standardized gene set score as the sole covariate. Each row corresponds to one PTCL molecular subtype; sample sizes are given in parentheses next to the subtype label. Points indicate HR point estimates and horizontal bars span the 95% Wald confidence interval. The vertical dashed line marks HR = 1 (no association); HR < 1 indicates that higher gene set activity is associated with longer overall survival, while HR > 1 indicates the opposite. The hazard ratio axis is shown on a base 10 logarithmic scale. Subtypes are ordered by HR point estimate from top to bottom.

References

1. Smid, A. I., Garforth, S. J., Obaid, M. S., Bollons, H. R. & James, J. R. Pre-T cell receptor localization and trafficking are independent of its signaling. *The Journal of cell biology* **222**; 10.1083/jcb.202212106 (2023).

2. Akashi, K., Kondo, M., Freeden-Jeffry, U. von, Murray, R. & Weissman, I. L. Bcl-2 rescues T lymphopoiesis in interleukin-7 receptor-deficient mice. *Cell* **89,** 1033–1041; 10.1016/s0092-8674(00)80291-3 (1997).

3. Boudil, A. *et al.* IL-7 coordinates proliferation, differentiation and Tcra recombination during thymocyte β-selection. *Nature immunology* **16,** 397–405; 10.1038/ni.3122 (2015).

4. Hong, C., Luckey, M. A. & Park, J.-H. Intrathymic IL-7: the where, when, and why of IL-7 signaling during T cell development. *Seminars in immunology* **24,** 151–158; 10.1016/j.smim.2012.02.002 (2012).

5. Yao, Z. *et al.* Stat5a/b are essential for normal lymphoid development and differentiation. *Proceedings of the National Academy of Sciences of the United States of America* **103,** 1000–1005; 10.1073/pnas.0507350103 (2006).

6. Villarino, A. *et al.* Signal transducer and activator of transcription 5 (STAT5) paralog dose governs T cell effector and regulatory functions. *eLife* **5**; 10.7554/eLife.08384 (2016).

7. Shin, B., Chang, S. J., MacNabb, B. W. & Rothenberg, E. V. Transcriptional network dynamics in early T cell development. *The Journal of experimental medicine* **221**; 10.1084/jem.20230893 (2024).

8. Kueh, H. Y. *et al.* Asynchronous combinatorial action of four regulatory factors activates Bcl11b for T cell commitment. *Nature immunology* **17,** 956–965; 10.1038/ni.3514 (2016).

9. Ye, S. K. *et al.* The IL-7 receptor controls the accessibility of the TCRgamma locus by Stat5 and histone acetylation. *Immunity* **15,** 813–823; 10.1016/S1074-7613(01)00230-8 (2001).

10. Hosokawa, H. & Rothenberg, E. V. How transcription factors drive choice of the T cell fate. *Nature reviews. Immunology* **21,** 162–176; 10.1038/s41577-020-00426-6 (2021).

11. Zhang, Q., Wang, H. Y., Liu, X. & Wasik, M. A. STAT5A is epigenetically silenced by the tyrosine kinase NPM1-ALK and acts as a tumor suppressor by reciprocally inhibiting NPM1-ALK expression. *Nature medicine* **13,** 1341–1348; 10.1038/nm1659 (2007).

12. Garces de Los Fayos Alonso, I. *et al.* PDGFRβ promotes oncogenic progression via STAT3/STAT5 hyperactivation in anaplastic large cell lymphoma. *Molecular cancer* **21,** 172; 10.1186/s12943-022-01640-7 (2022).

13. Schmalzbauer, B. S. *et al.* CDK6 Degradation Is Counteracted by p16INK4A and p18INK4C in AML. *Cancers* **14**; 10.3390/cancers14061554 (2022).

14. Kollmann, K. *et al.* A kinase-independent function of CDK6 links the cell cycle to tumor angiogenesis. *Cancer cell* **24,** 167–181; 10.1016/j.ccr.2013.07.012 (2013).

15. Iyer, A., Hennessey, D. & Gniadecki, R. Clonotype pattern in T-cell lymphomas map the cell of origin to immature lymphoid precursors. *Blood Advances* **6,** 2334–2345; 10.1182/bloodadvances.2021005884 (2022).

16. Crescenzo, R. *et al.* Convergent Mutations and Kinase Fusions Lead to Oncogenic STAT3 Activation in Anaplastic Large Cell Lymphoma. *Cancer cell* **27,** 744; 10.1016/j.ccell.2015.04.014 (2015).

17. Malcolm, T. I. M. *et al.* Anaplastic large cell lymphoma arises in thymocytes and requires transient TCR expression for thymic egress. *Nature communications* **7,** 10087; 10.1038/ncomms10087 (2016).

18. Hassler, M. R. *et al.* Insights into the Pathogenesis of Anaplastic Large-Cell Lymphoma through Genome-wide DNA Methylation Profiling. *Cell reports* **17,** 596–608; 10.1016/j.celrep.2016.09.018 (2016).

19. Moti, N. *et al.* Anaplastic large cell lymphoma-propagating cells are detectable by side population analysis and possess an expression profile reflective of a primitive origin. *Oncogene* **34,** 1843–1852; 10.1038/onc.2014.112 (2015).

20. Turner, S. An Exploration into the Origins and Pathogenesis of Anaplastic Large Cell Lymphoma, Anaplastic Lymphoma Kinase (ALK)-Positive. *Cancers* **9,** 141; 10.3390/cancers9100141 (2017).

21. Frutos Díaz-Alejo, J. *et al.* Molecular Insights into the Diagnosis of Anaplastic Large Cell Lymphoma: Beyond Morphology and Immunophenotype. *International journal of molecular sciences* **26**; 10.3390/ijms26125871 (2025).

22. Suske, T. *et al.* Hyperactive STAT5 hijacks T cell receptor signaling and drives immature T cell acute lymphoblastic leukemia. *The Journal of clinical investigation* **134**; 10.1172/JCI168536 (2024).

23. Savage, K. J. *et al.* ALK- anaplastic large-cell lymphoma is clinically and immunophenotypically different from both ALK+ ALCL and peripheral T-cell lymphoma, not otherwise specified: report from the International Peripheral T-Cell Lymphoma Project. *Blood* **111,** 5496–5504; 10.1182/blood-2008-01-134270 (2008).

24. Hu, M. G. *et al.* CDK6 kinase activity is required for thymocyte development. *Blood* **117,** 6120–6131; 10.1182/blood-2010-08-300517 (2011).

25. Scheiblecker, L. *et al.* CDK6 kinase inhibition unmasks metabolic dependencies in BCR::ABL1+ leukemia. *Cell death & disease* **16,** 107; 10.1038/s41419-025-07434-1 (2025).

26. Bellutti, F. *et al.* CDK6 Antagonizes p53-Induced Responses during Tumorigenesis. *Cancer discovery* **8,** 884–897; 10.1158/2159-8290.CD-17-0912 (2018).

27. Liang, H.-C. *et al.* Super-enhancer-based identification of a BATF3/IL-2R-module reveals vulnerabilities in anaplastic large cell lymphoma. *Nature communications* **12,** 5577; 10.1038/s41467-021-25379-9 (2021).

Reviewer #1 (Remarks to the Author):

The authors have carefully revised the manuscript and provided detailed responses to my comments. The concerns raised during the previous review round have been satisfactorily addressed, and the additional data and clarifications have significantly strengthened the study.

In my opinion, the manuscript is now suitable for publication and meets the standards of Nature Communications. I therefore support its acceptance for publication.

We thank the reviewer for the positive assessment of our manuscript.

Reviewer #2 (Remarks to the Author):

Dear Editor,

the Authors have satisfactorily revised the manuscript which is now clearly improved. In particular, they have strengthened the conclusions on human ALCL and the potential clinical value of the identified signature.

We thank the reviewer for the favorable evaluation and for recognizing our strengthened human ALCL findings.
